# Fungal endophytes of *Vanilla planifolia* across Réunion Island: isolation, distribution and biotransformation

**DOI:** 10.1186/s12870-015-0522-5

**Published:** 2015-06-14

**Authors:** Shahnoo Khoyratty, Joëlle Dupont, Sandrine Lacoste, Tony Lionel Palama, Young Hae Choi, Hye Kyong Kim, Bertrand Payet, Michel Grisoni, Mireille Fouillaud, Robert Verpoorte, Hippolyte Kodja

**Affiliations:** Université de La Réunion, UMR PVBMT, 15 avenue René Cassin, CS 92003-97744 Saint Denis Cedex 9 La Réunion, France; Natural Products Laboratory, Institute of Biology, Leiden University, Sylviusweg, 72, 2333 BE Leiden, The Netherlands; GIP CYROI, 2 rue Maxime Rivière, 97490 Sainte-Clotilde La Réunion, France; Département Systématique et Evolution, Muséum National d’Histoire Naturelle, UMR OSEB 7205, CP 39, 57 rue Cuvier, 75231 Paris, Cedex 05 France; Institut des Sciences Analytiques (CNRS/ENS Lyon/UCB Lyon1), Centre de RMN à Très Hauts Champs, Université de Lyon, 69100 Villeurbanne, France; Université de La Réunion, LCSNSA EA 2212, 15 avenue René Cassin, CS 92003-97744 Saint Denis Cedex 9 La Réunion, France; Centre de coopération internationale en recherche agronomique pour le développement (CIRAD), Station de Ligne-Paradis & Pôle de protection des plantes, 7 chemin de l’IRAT, 97410 Saint-Pierre La Réunion, France; UMR PVBMT, Faculté des Sciences et Technologies, Université de La Réunion, 15, Avenue René Cassin, BP 7151 Saint-Denis Cédex 09 Ile de la Réunion, France

**Keywords:** Endophytes, Distribution, Diversity, Biotransformation, Vanilla, Interaction

## Abstract

**Background:**

The objective of the work was to characterize fungal endophytes from aerial parts of *Vanilla planifolia*. Also, to establish their biotransformation abilities of flavor-related metabolites. This was done in order to find a potential role of endophytes on vanilla flavors.

**Results:**

Twenty three MOTUs were obtained, representing 6 fungal classes. Fungi from green pods were cultured on mature green pod based media for 30 days followed by ^1^H NMR and HPLC-DAD analysis. All fungi from pods consumed metabolized vanilla flavor phenolics. Though *Fusarium proliferatum* was recovered more often (37.6 % of the isolates), it is *Pestalotiopsis microspora* (3.0 %) that increased the absolute amounts (quantified by ^1^H NMR in μmol/g DW green pods) of vanillin (37.0 × 10^−3^), vanillyl alcohol (100.0 × 10^−3^), vanillic acid (9.2 × 10^−3^) and *p*-hydroxybenzoic acid (87.9 × 10^−3^) by significant amounts.

**Conclusions:**

All plants studied contained endophytic fungi and the isolation of the endophytes was conducted from plant organs at nine sites in Réunion Island including under shade house and undergrowth conditions. Endophytic variation occured between cultivation practices and the type of organ. Given the physical proximity of fungi inside pods, endophytic biotransformation may contribute to the complexity of vanilla flavors.

**Electronic supplementary material:**

The online version of this article (doi:10.1186/s12870-015-0522-5) contains supplementary material, which is available to authorized users.

## Background

The genus Vanilla is a member of the *Orchidaceae* family and comprises of approximately 100 species and among them, *Vanilla planifolia* is the most important source of natural vanilla flavor [1]. Natural vanilla flavor is the number one flavor tonality in the world as it is subtle, but complex [2]. Over 200 compounds have already been isolated and identified from vanilla beans. These compounds vary in concentration depending on the region where the beans are harvested [3]. Four major flavor related components (*p*-hydroxybenzoic acid, *p*-hydroxybenzaldehyde, vanillic acid and vanillin) are used as marker compounds to determine quality and authenticity of vanilla products. For authentic unadulterated vanilla extracts, the ratios between the four components are fixed within a certain range [4]. In Réunion Island, vanilla plants are either cultivated in the undergrowth or in shade houses. Vanilla pods grown in the undergrowth appeared to display substantial qualitative differences of vanillin and vanillic acid contents in comparison to those grown under shade-house conditions. Parameters responsible for such a difference have not been identified yet [5]. The major vanilla flavor constituents are present as glycosides in the pods prior to curing [6]. In order to allow the development of flavor, green pods undergo post-harvest processing. The exact method for post-harvest processing and curing of vanilla pods varies across the region of the world where vanilla is produced.

Endophytic fungi are defined functionally by their occurrence within asymptomatic tissues of plants [7]. In spite of the ubiquitous features, the scale of their diversity, their host range, and geographic distributions much about endophytes is still unknown for many plants including vanilla. Endophytic fungi can either be transmitted vertically or horizontally. Vertical transmission occurs when fungi are transferred from the host to the offspring via host tissues. Horizontal transmission occurs when fungi are transferred to the host via spores e.g. through aerial means. Endophytes can be involved in biomass production and nutrient cycling in the plant [7]. Previously Porras-Alfaro and Bayman [8] isolated non-pathogenic fungi from inside asymptomatic roots of vanilla plants. Mycorrhizal fungi interact symbiotically with roots through an association of the mycelium (typically basidiomycete) while the hyphae form a mass around the rootlets or penetrate root cells. They are absent from the outer root cortex and hence differ from endophytes that are present deeper inside plant tissues. The mycorrhizal fungi *Ceratobasidium* spp., *Thanatephorus* spp. and *Tulasnella* spp. were found to be associated to different species of vanilla Porras-Alfaro and Bayman [8]. Morphological identification followed by elongation factor gene sequence analysis showed that several *Fusarium* spp. are present in vanilla plants in Indonesia [9].

**Fig. 1:**
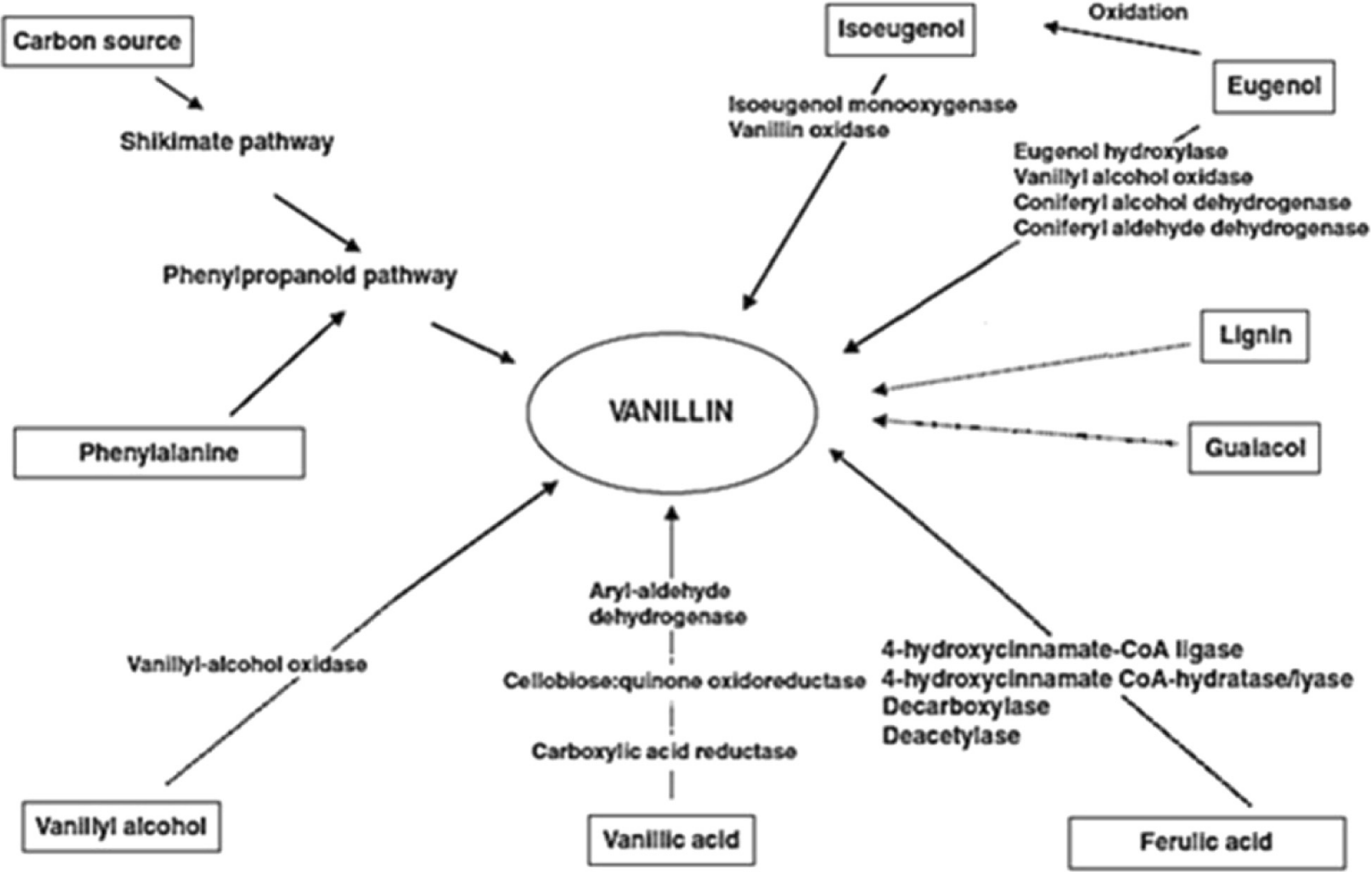
Different microbial routes to vanillin (adapted from [38])

Metabolomics is defined as both the qualitative and quantitative analysis of all primary and secondary metabolites of an organism [10]. Two chemical analysis techniques used in metabolic profiling include ^1^H nuclear magnetic resonance (NMR) spectroscopy and high-performance liquid chromatography (HPLC). For instance, high-performance liquid chromatography - diode array detector (HPLC-DAD) analysis showed the presence of 3,4-O-(*Z*)-dicaffeoylquinic acid and quercetin-7-*O*-glucoside as the main components from *in vitro* microplants of *Hyptis marrubioides* Epling inoculated with bacterial and fungal endophytic isolates [11]. Similarly metabolomic methods such as these can be effective to decipher the potential involvement of endophytic fungi in the production of secondary metabolites. Despite being a simple molecule, natural vanillin biosynthesis from *V. planifolia* plants remains controversial. In fact, there is still some disagreement over the exact cell types that produce vanillin. A possible reason for such controversy stems from the fact that vanillin is a simple structure that lends itself to multiple possible theoretical biosynthetic pathways and due to the general promiscuity of many enzymes of plant phenolic metabolism; it is possible to find evidence to support any of these pathways from *in vitro* biochemical approaches [12]. Hence, the biosynthetic pathway of vanillin still needs full proof on the level of enzymes and genes. Vanillin production from natural sources can either be through the biotransformation of an existing precursor compound or by *de novo* synthesis of a precursor where the organism produces an intermediate in vanillin biosynthesis. Biotransformation of vanillin precursors is not limited to vanilla plants, but can also be achieved with microorganisms (Fig. 1), e.g. fungi.

For instance, *p*-coumaric acid is converted *in vitro* to *p*-hydroxybenzaldehyde by the fungus *Paecilomyces variotii* grown on minimal medium containing basal inorganic salts with *p-coumaric* acid as a sole carbon source [13]. Vanillic acid is formed from vanillin by *Hormodendrum* sp. grown *in vitro* on basal medium [14]. Vanillyl alcohol is made by *Pestalotia palmarum* from ferulic acid grown *in vitro* on synthetic medium supplied with glucose [15]. Furthermore, the simplicity of vanillin structure has led to the use of various precursors in the microbial or enzymatic process of vanillin production: lignin, curcumin, siam benzoin resin, phenolic stilbenes, isoeugenol, eugenol, ferulic acid, aromatic amino acids, and glucose via *de novo* biosynthesis while several fungi have the capacity to metabolize the aforementioned precursors. A similarity can thus be seen between biotransformations in vanilla cells with regards to metabolites related to vanilla flavor, and fungal biotransformations of these compounds. Given such similarities in the biosynthetic pathways of polyphenols in vanilla plants and fungi, it is not surprising that a possible role of microorganisms in vanilla has been investigated before. Roling et al. [16] and Dunphy and Bala [2, 17], for example, studied a possible involvement of microorganisms during the curing of the pods all pointing principally to the occurrence of bacteria and actinomycetes. The current study concerns another aspect though: the possible role of fungal endophytes in the vanilla plant and the green pods in the formation of vanilla flavor related compounds. So far no fungal endophytes have been isolated from aerial parts of the plant.

In this work, endophytes were isolated from organs, across regions of Réunion island and from two cultural practices. Particular emphasis was placed on finding endophyte assemblage in pods, finding probable transmission methods and finding the diversity. The focus was then on establishing a link between endophytes from pods and flavor development. Thus, the biotransformation reactions from fungi were compared. The amounts of biotransformed flavor metabolites and ratios of quality marker metabolites were determined after the biotransformation reactions by the fungi grown on a green pod based media.

## Results and Discussion

Endophytes were first isolated from vanilla in this work and their diversity and distribution were characterized. A series of experiments were then conducted to investigate the potential effects of the presence of endophytes in green pods on flavor development in such pods given that vanilla are prized for flavor. The experiments were based on elucidating the biotransformation abilities of such fungal endophytes. Additionally, a pathogen (Molecular Operational Taxonomic Unit (MOTU) 24 –Fo72 *Fusarium oxysporum* f.sp. *vanillae*) was also isolated.

### Diversity of endophytes

**Fig. 2:**
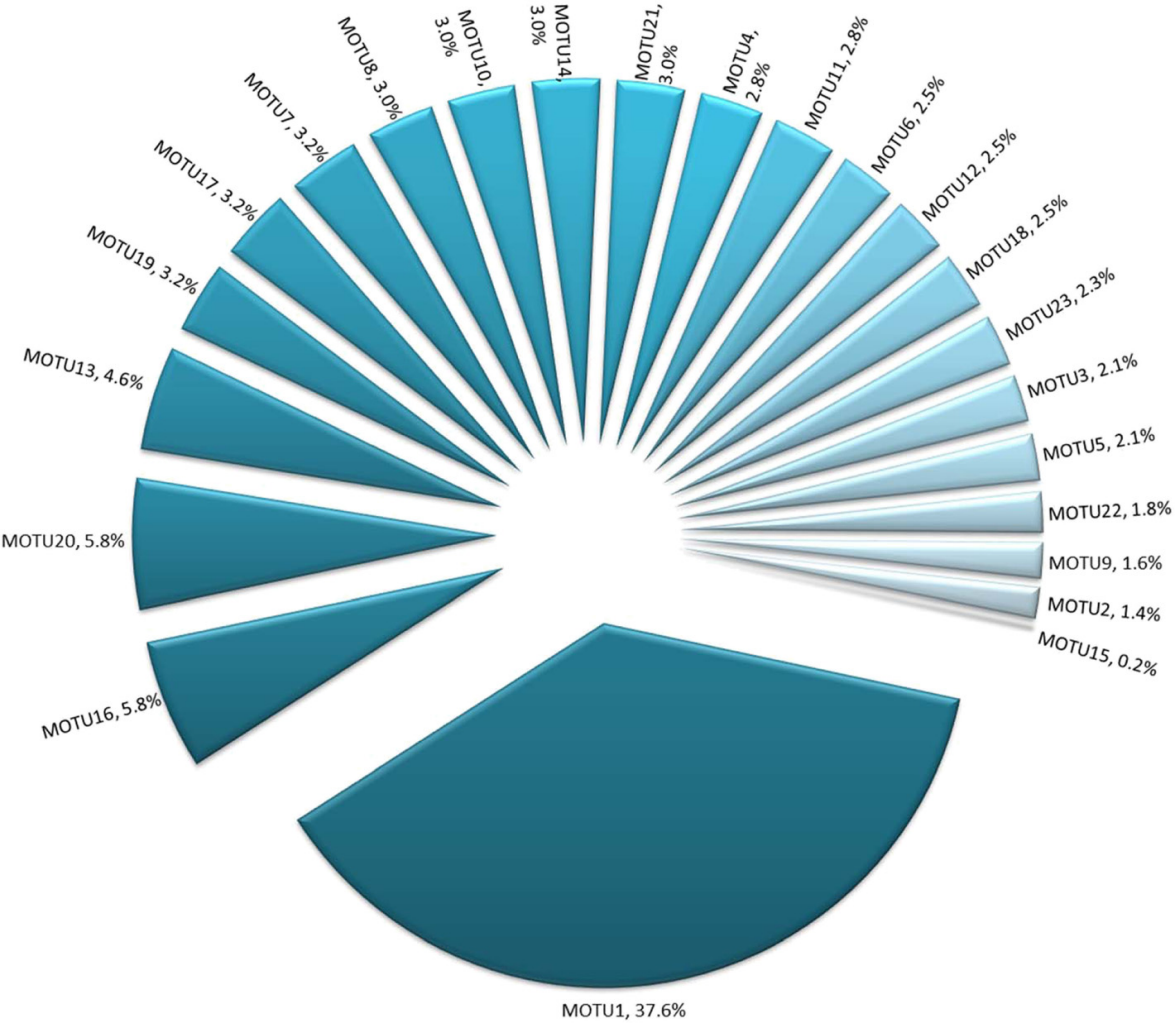
Percentage of each MOTU in relation to the total number of fungi isolated. The following MOTUs are represented in the diagram: MOTU1 *Fusarium proliferatum*, MOTU2 *Fusarium scirpi*, MOTU3 *Fusarium oxysporum*, MOTU4 *Acremonium implicatum*, MOTU5 *Purpureocillium lilacinum*, MOTU6 *Phomopsis phyllanthicola*, MOTU7 *Diaporthe phaseolorum*, MOTU8 *Nemania bipapillata*, MOTU9 *Xylaria* sp., MOTU10 *Pestalotiopsis microspora*, MOTU11 *Colletotrichum gloeosporioides*, MOTU12 *Colletotrichum* sp2, MOTU13 *Nigrospora* sp1, MOTU14 *Nigrospora* sp 2, MOTU15 *Delitschia chaetomioides*, MOTU16 *Botryosphaeria ribis*, MOTU17 *Guignardia mangiferae*, MOTU18 *Mycosphaerella marksii*, MOTU19 *Penicillium citrinum*, MOTU20 *Aspergillus fumigatus*, MOTU21 *Sarcosomataceous* spp., MOTU22 *Perenniporia nanlingensis* and MOTU23 *Cunninghamella blakesleana*

After identifying the isolated fungi (Additional file 1: Table S1), it was found that out of 450 sampled tissues, 220 yielded endophytes (Table 1) from which 434 isolates were recovered (Table 3). Hence, at least 48.9 % of sampled tissues were infected, given that some fungal endophytes may not be cultivable. This low percentage is due to young leaves (1 and 3 weeks old) that were free of endophyte or a low infection level might have hampered isolation of the fungi. Twenty three different MOTUs were isolated from the collected samples, representing six classes (Sordariomycetes, Dothideomycetes, Eurotiomycetes, Pezizomycetes, Agaricomycetes, Zygomycetes; Table 3). *Fusarium proliferatum* (MOTU1) was, by far, the most abundant fungus accounting for 37.6 % of the isolates (Table 3) and the most common fungus, occurring at all the nine sites sampled (Table 2 and Fig. 2).

**Table 1:** Infection frequencies among surveyed organs over the sites sampled and distribution in relation to geographic origin and management types

Sites	Organs	Number of tissues infected over the total number of sampled tissues									
		St. Andre		St. Anne		St Rose	Bois blanc	Takamaka	Mare longue	Basse valle	Total
		Sampled tissues / number of tissues yielding endophytes		Sampled tissues / number of tissues yielding endophytes		Sampled tissues / number of tissues yielding endophytes	Sampled tissues / number of tissues yielding endophytes	Sampled tissues / number of tissues yielding endophytes	Sampled tissues / number of tissues yielding endophytes	Sampled tissues / number of tissues yielding endophytes	Sampled tissues / number of tissues yielding endophytes
Manament type		UG	SH	UG	SH	UG	UG	UG	UG	UG	
Fragments sampled / fragments infected	Ovaries	Not detected	15/11	Not detected	Not detected	Not detected	Not detected	Not detected	Not detected	Not detected	15/11
	Pods	15/9	15/14	15/9	15/14	15/15	15/9	15/11	15/11	15/10	135/102
	Leaf 1	Not detected	15/6	15/0	Not detected	15/0	Not detected	15/0	15/0	Not detected	75/6
	Leaf 3	Not detected	15/9	15/0	Not detected	15/0	Not detected	15/3	15/0	Not detected	75/12
	Leaf 5	Not detected	15/13	15/5	Not detected	15/7	Not detected	15/4	15/0	Not detected	75/29
	Leaf 15	Not detected	15/14	15/11	Not detected	15/8	Not detected	15/13	15/14	Not detected	75/60
Grand total											450/220

Managment type: undergrowth (UG), shade house (SH)

Footnote: The first youngest leaf collected is leaf rank 1 followed by 3, 5 and 15 leaf on the same branch with the 15th being the oldest

**Table 2:** Types and abundance of MOTUs (shown in brackets) in relation to geographic origin, management types and organs

Sites	Isolated MOTUS codes (number of times isolated)										Total
		St. Andre		St Anne		St Rose	Bois Blanc	Takamaka	Mare Longue	Basse Vallee	
	Organs	UC	SH	UG	SH	UG	UG	UG	UG	UG	
MOTU number/(Number of isolates)	Ovaries	Not detected	MOTU2(6) MOTU13(7) MOTU15(1) MOTU16(10) MOTU2O(11)	Not detected	Not detected	Not detected	Not detected	Not detected	Not detected	Not detected	35
	Pods	MOTU1(9)	MOTU1(5) MOTIT(14)	MOTU3(9)	MOTU1(6) MOTU23(1O) MOTUS(9) MOTU11(12) MOTU14(13) MOTU2O(14)	MOTU1(13) MOTU4(12) MOTU8(13) MOTU1O(13) MOTU16(15)	MOTU1(9)	MOTU1(11)	MOTU1(9) MOTU18(11) MOTU6(11)	MOTU1(10)	228
	Leaf 1	Not detected	MOTU1(6)	Not detected	Not detected	Not detected	Not detected	Not detected	Not detected	Not detected	6
	Leaf 3	Not detected	MOTU1(9)	Not detected	Not detected	Not detected	Not detected	MOTU1(3)	Not detected	Not detected	12
	Leaf 5	Not detected	MOTU1(12) MOTU13(13)	MOTU1(5)	Not detected	MOTU1(7)	Not detected	MOTU1(4)	Not detected	Not detected	41
	Leaf 15	Not detected	MOTU1(12) MOTU9(7) MOTU12(11) MOTU19(14)	MOTU1(11)	Not detected	MOTU1(8)	Not detected	MOTU1(7) MOTU21(13)	MOTU1(7) MOTU22(8) MOTU17(14)	Not detected	112
Grand total											434

Managment type: undergrowth (UG), shade house (SH)

Footnote: The first youngest leaf collected is leaf rank 1 followed by 3, 5 and 15 leaf on the same branch with the 15th being the oldest

*Botryosphaeria ribis* (MOTU16) and *Aspergillus fumigatus* (MOTU20) were the second most abundant taxa, each accounting for 5.8 % of the isolates. Both were distributed over two sites, Saint André and Sainte Rose for MOTU16 and Saint André and Sainte Anne for MOTU20 (Table 2). Other endophytes were rarely isolated, each occurring only in one site and one organ with MOTU15, *D. chaetomioides,* being the less abundant at 0.2 % of all isolates (Tables 2 and 3 and Fig. 2). The three mycorrhizal fungi isolated from roots of different species of vanilla by Porras-Alfaro and Bayman [8] are members of the class *Agaricomycetes*. Only one isolated fungus in this work belongs to the class *Agaricomycetes* although that fungus is an endophyte and not a mycorrhiza (Table 3, MOTU22 *P. nanlingensis*). Furthermore, the fungus originated from inside the organ, and hence not through superficial contamination from the root of the plant for instance, given that after surface sterilization, the organ surface was touched onto potato dextrose agar (PDA) media with no fungal growth obtained. Fungal growth was obtained only when the organ was split open and when the interior of the organ exposed to PDA. 16 fungal genera were isolated from *Holcoglossum* plants which, like vanilla, are also members of the family *Orchidaceae* and the fungi belonged to three classes, *Sordariomycetes*, *Dothideomycetes* and *Agaricomycetes* [18]. In comparison, a high number of 21 fungal genera were isolated from vanilla plants in this study representing six classes (Sordariomycetes, Dothideomycetes, Eurotiomycetes, Pezizomycetes, Agaricomycetes, Zygomycetes; Table 3) with *Sordariomycetes* being the dominant class (60 %, 14/23 MOTUs). Which is in line with the fact that endophytic *Sordariomycetes* have a high frequency of occurrence within tropical plants [19].

**Table 3:** List and abundance of molecular operational taxonomic units (MOTUs) from endophytes identified in this study

MOTU number	Fungal species	Class / Order	Abundance (number of isolates)
MOTU1	*Fusarium proliferatum*	Sordariomycetes / Hypocreales	163
MOTU2	*Fusarium scirpi*	Sordariomycetes / Hypocreales	6
MOTU3	*Fusarium oxysporum*	Sordariomycetes / Hypocreales	9
MOTU4	*Acremonium implicatum*	Sordariomycetes / Hypocreales	12
MOTU5	*Purpureocillium lilacinum*	Sordariomycetes / Hypocreales	9
MOTU6	*Phomopsis phyllanthicola*	Sordariomycetes / Diaporthales	11
MOTU7	*Diaporthe phaseolorum*	Sordariomycetes / Diaporthales	14
MOTU8	*Nemania bipapillata*	Sordariomycetes / Xylariales	13
MOTU9	*Xylaria* sp.	Sordariomycetes / Xylariales	7
MOTU10	*Pestalotiopsis microspora*	Sordariomycetes / Xylariales	13
MOTU11	*Colletotrichum gloeosporioides*	Sordariomycetes / Glomerellaceae	12
MOTU12	*Colletotrichum* sp2	Sordariomycetes / Glomerellaceae	11
MOTU13	*Nigrospora* sp1	Sordariomycetes / Trichosphaeriales	20
MOTU14	*Nigrospora* sp 2	Sordariomycetes / Trichosphaeriales	13
MOTU15	*Delitschia chaetomioides*	Dothideomycetes / Pleosporales	1
MOTU16	*Botryosphaeria ribis*	Dothideomycetes / Botryosphaeriales	25
MOTU17	*Guignardia mangiferae*	Dothideomycetes / Botryosphaeriales	14
MOTU18	*Mycosphaerella marksii*	Dothideomycetes / Capnodiales	11
MOTU19	*Penicillium citrinum*	Eurotiomycetes / Eurotiales	14
MOTU20	*Aspergillus fumigatus*	Eurotiomycetes / Eurotiales	25
MOTU21	*Sarcosomataceae* spp.	Pezizomycetes / Pezizales	13
MOTU22	*Perenniporia nanlingensis*	Agaricomycetes / Polyporales	8
MOTU23	*Cunninghamella blakesleana*	Zygomycetes / Mucorales	10
Total			434

**Fig. 3:**
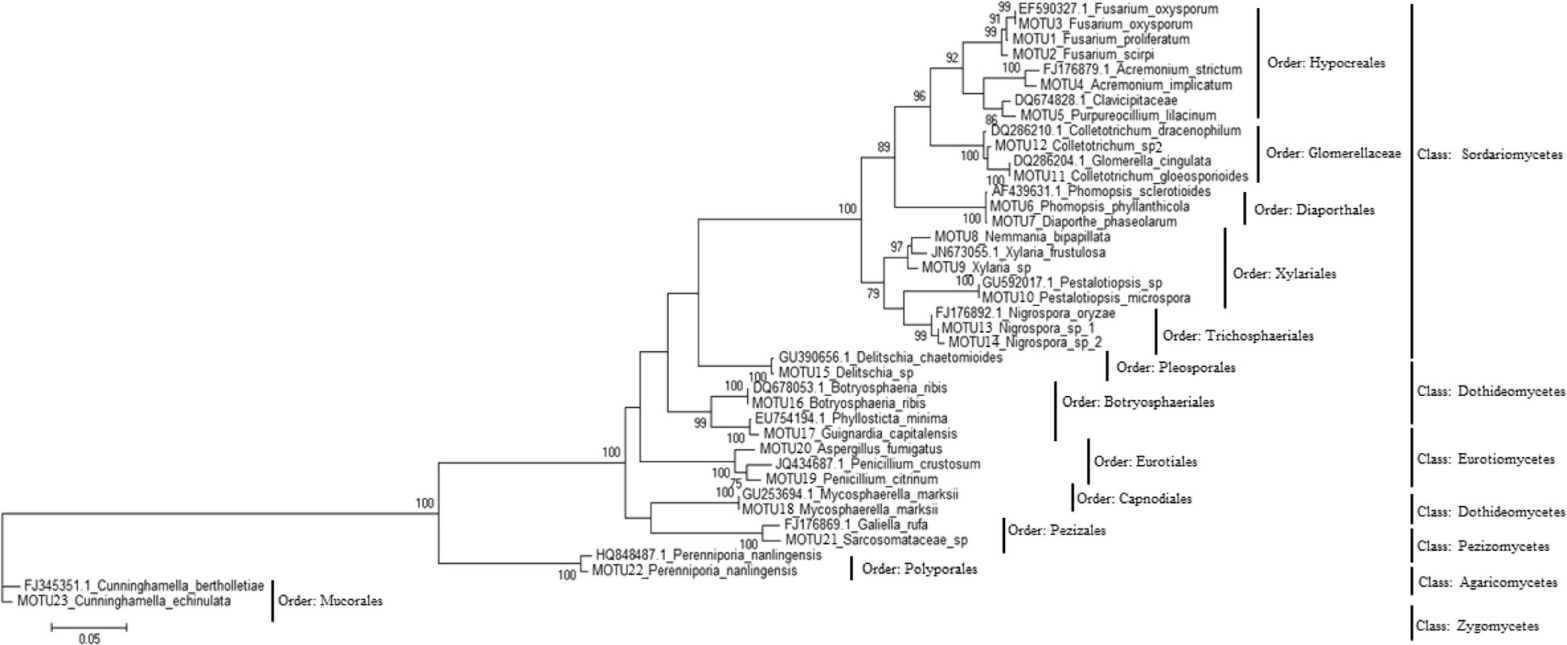
Phylogenetic relationships among the 23 identified MOTUs. The analysis was based on LSU rDNA sequences. In cases where the sequences are from NCBI GenBank, the accession number is shown. The number of bootstrap replications used was 1500

A phylogenetic analysis was performed using Large-subunit ribosomal RNA gene (LSU rDNA) sequences from the 23 MOTUs together with 17 sequences from GenBank (http://www.ncbi.nlm.nih.gov/genbank/). Although *P. microspora* is a member of the order *Xylariales* (a common source of endophytes), MOTU10 formed a monophyletic clade with members of the order *Trichosphaeriales* (MOTU13 *Nigrospora* sp 1 and MOTU14 *Nigrospora* sp 2) but this clade is not supported (Fig. 3).

The majority of the isolated endophytes belonged to the class *Sordariomycetes* (60 %, 14 out of 23 isolated MOTUs) which consist of members of the orders *Hypocreales* (consisting of 199 isolates making 5 MOTUs), *Xylariales* (consisting of 33 isolates making 3 MOTUs), *Diaporthales* (consisting of 25 isolates making 2 MOTUs) and *Glomerellaceae* (consisting of 23 isolates making 2 MOTUs). *Dothideomycetes* and *Eurotiomycetes* were the next most common classes both representing 11.8 and 9 % of all isolated MOTUs respectively. Classes *Pezizomycetes*, *Zygomycetes* and *Agaricomycetes* were rare, with only one MOTU representative of each.

### Mode of transmission

Symbiont transmission perpetuates symbioses through host generations. Horizontally transmitted symbionts are acquired through the environment while vertically transmitted symbionts are often transferred through the female germ line but mixed modes of transmission also exist. In order to establish the method of endophyte transmission in the plant, ovaries which have petals that are closed as well as those with petals that are open were collected under shade-house conditions at St. André. Five MOTUs were isolated (MOTU2 *F. scirpi*, MOTU13 *Nigrospora* sp1, MOTU15 *D. chaetomioides*, MOTU16 *B. ribis* and MOTU20 *A. fumigatus* (Table 2)) from ovaries with opened petals only. Hence, fungi were recovered from ovaries with opened petals only and not from ovaries with closed petals. Ovaries with opened petals are exposed to air whereas those with closed petals are not. Hence fungal endophytes only entered the ovaries through aerial means when the petals are opened. Ovaries of *V. planifolia* seemed endophyte free at emergence. Thus, the five isolated fungal MOTUs from ovaries with opened flowers were most likely transmitted horizontally however endophytes may colonize fruits later in development. The discovery that MOTU1 *F. proliferatum* occurs in pods and leaves opens the way to the idea that some endophytes may issue from other vegetative tissues. Thus further research is required to confirm which event occurred. The possibility for a horizontal transmission of endophytes in vanilla pods would be similar to the case of cacao where fruits are endophyte-free at emergence, but then accumulate diverse endophytes from spore rain in the environment [20]. With ovary maturation, endophyte populations in cranberries vary [21]. Similarly, the fungal MOTUs isolated from *V. planifolia* ovaries with opened petals in the shade house at St. André differed from those identified from 8 months post-pollination pods (MOTU1 *F. proliferatum* and MOTU7 *D. phaseolorum*) at the same location.

### Endophyte isolation from different organs

Leaves of different ranks (15 being the oldest followed by 5, 3 and 1 being the youngest) and pods were sampled equally from plants grown in the undergrowth at St. Anne, St. Rose, Takamaka and Mare Longue.

**Fig. 4:**
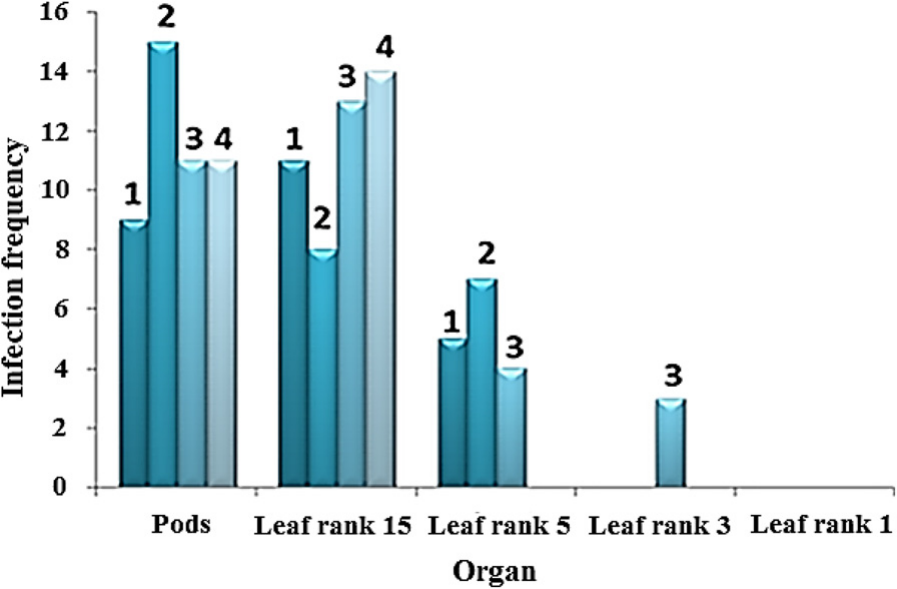
Organ infection frequencies in plants originating from the undergrowth. The first youngest leaf collected is leaf rank 1 followed by 3, 5 and 15 leaf on the same branch with the 15th being the oldest. Infection frequency over a total of 15 organs collected from plants grown in the undergrowth at 1: St. Anne, 2: St. Rose, 3: Takamaka and 4: Mare Longue

Leaf ranks 5, 3 and 1 had lower frequencies of infection across regions at 27, 5 and 0 % respectively. Thus, the frequency of infection was higher in older leaves (rank 15) than in younger ones (rank 1) even when each region is compared (Fig. 4). Despite the similarity between the number of infected samples between the pods and rank 15 leaves, the total number of MOTUs recovered from the infected samples differed for pods (8 different MOTUs isolated) and rank 15 leaves (4 different MOTUs isolated). Furthermore, with the exception of MOTU1 *F. proliferatum*, the isolated MOTUs differed between pods (MOTUs 3 *F. oxysporum*, 4 *A. implicatum*, 6 *P. phyllanthicola*, 8 *N. bipapillata*, 10 *P. microspora*, 16 *B. ribis* and 18 *M. marksii*) and rank 15 leaves (MOTUs 17 *G. mangiferae*, 21 *Sarcosomataceous* spp. and 22 *P. nanlingensis*) (Table 2). The frequency of infection was higher in older leaves (rank 15) than in younger ones (rank 1) also the fungal MOTUs isolated from pods differed from those of leaves of *V. planifolia*.

### Endophyte communities over the Island

Among four regions (St. Anne, St. Rose, Takamaka and Mare Longue) where ranks 1, 3, 5 and 15 leaves as well as 8 months old green pods post pollination were collected from plants in the undergrowth, the region where the endophyte diversity from pods was the highest is St. Rose (MOTUs 1 *F. proliferatum*, 4 *A. implicatum*, 8 *N. bipapillata*, 10 *P. microspora* and 16 *B. ribis*) followed by Mare Longue (MOTUs 1 *F. proliferatum*, 18 *M. marksii* and 6 *P. phyllanthicola*) (Table 2). With the exception of MOTU1, the fungi isolated from pods from St. Rose (MOTUs 4, 8, 10 and 16) and Mare Longue (MOTUs 18, 6) differed. This preliminary result suggests that, even within the same organ (pods) different fungi are present depending on the region where the pod originates. In order to further confirm this preliminary result, the sampling must be amplified followed by next-generation sequencing (NGS) and the application of rarefaction curves to show that the number of MOTUs isolated reached a plateau at each site. However, such is not the aim of the current work.

### Endophyte assemblage in pods in relation to cultivation practices

In order to assess the distribution of endophytes in pods from plants grown under shade house conditions relative to those plants grown in the undergrowth, fungal endophytes were isolated from pods originating from St. André and St. Anne under shade house conditions and from the undergrowth. Pods collected from shade houses contained a more diverse endophyte assemblage than pods collected in the undergrowth from the same sites (Table 2). As such, 6 MOTUs (MOTU1 *F. proliferatum*, MOTU5 *P. lilacinum*, MOTU11 *C. gloeosporioides*, MOTU14 *Nigrospora* sp 2, MOTU20 *A. fumigatus* and MOTU23 *C. blakesleana*) and 1 MOTU (MOTU7 *D. phaseolorum*) were isolated additionally from pods originating from shade houses at St. Anne and St. André respectively when compared to their counterparts from the undergrowth. Fungal MOTUs isolated from pods from the undergrowth at St. André and St. Anne includes two *Fusarium* spp.: MOTU1 *F. proliferatum* and MOTU3 *F. oxysporum* respectively. The percentage of infection of pods was higher in shade houses (93 %) than in the undergrowth (60 %). The frequency of infected pods was higher for plants from shade houses than those from the undergrowth at both St. André and St. Anne. Being an epiphyte, *V. planifolia* acquires moisture from the air through its aerial roots. As a consequence of this adaptation, shade houses are fitted with micro-sprinklers that keep the plant environment humid (80 % relative humidity (RH), 12 h). Given that a shade house is a closed system, the humidity in the air remains high for a prolonged period of time. The high humidity could explain the higher frequency of infection of pods in shade houses compared to those that originate from the undergrowth (70 % RH, fluctuating over the day) given that a high level of wetness of an organ may favor spore germination and survival of fungi and would thus increase endophyte colonization [22]. Fungal endophytes do not elicit an immune response in a plant which is why plants do not show any symptoms. However, higher humidity promotes a higher inoculum level increasing the chances of infection.

### Identifying flavor related metabolites

After isolating and characterizing the endophytes from vanilla, a series of experiments were conducted to elucidate the biotransformation abilities of selected fungal endophytes. There are numerous examples where cultural conditions in which fungi are placed affect their biotransformation reactions. For example, the amount of the bioactive secondary metabolite arundifungin produced by the endophytic fungus *Arthrinium* isolated from plant roots of *Apiospora montagnei* Sacc. changes depending on the time of incubation, temperature and pH of the culture medium [23]. As a consequence, the media on which the fungi were cultured in the laboratory was made to be the closest to that of the conditions in the green pod where they were isolated. Thus, to investigate the potential changes that fungal endophytes produce on flavor related metabolites in green vanilla pods, experiments were conducted where fungi isolated from mature green pods (8 months after pollination) were cultured on a medium composed of lyophilized and autoclaved green pod material. The lyophilized green pod material was the only source of available nutrients for fungi to grow in the experiments. Consequently any fungal growth is due to the ingredients of the green pods, which includes various primary metabolites, including sugars and amino acids as well as the various phenolics including vanillin glycoside. Endophytic fungi *D. phaseolorum* (MOTU7), *P. microspora* (MOTU10), *F. oxysporum* (MOTU24), *Nigrospora* sp (MOTU13), *N. bipapillata* (MOTU8), *M. marksii* (MOTU18), *A. implicatum* (MOTU4), *B. ribis* (MOTU16 - 61G1 isolated at St. Rose, Reunion Island), *C. gloeosporioides* (MOTU11), *F. proliferatum* (MOTU1), *B. ribis* (MOTU16 – 25 isolated at St. Anne, Reunion Island), *P. phyllanthicola* (MOTU6) and a pathogen *F. oxysporum* (MOTU24) were used for such experiments. Preliminary experiments were carried out to find the growth rate on 8 month old pod based media of the fungi selected for this work. It was found that on average the fungi covered a 90 mm petri dish in 30 days. Hence, 30 days of culture was the time retained for the experiments. After 30 days of culture, the medium was analyzed through proton nuclear magnetic resonance (^1^H NMR) so as to assess the biotransformation reactions performed by the fungi onto metabolites related to flavors in vanilla.

**Fig. 5:**
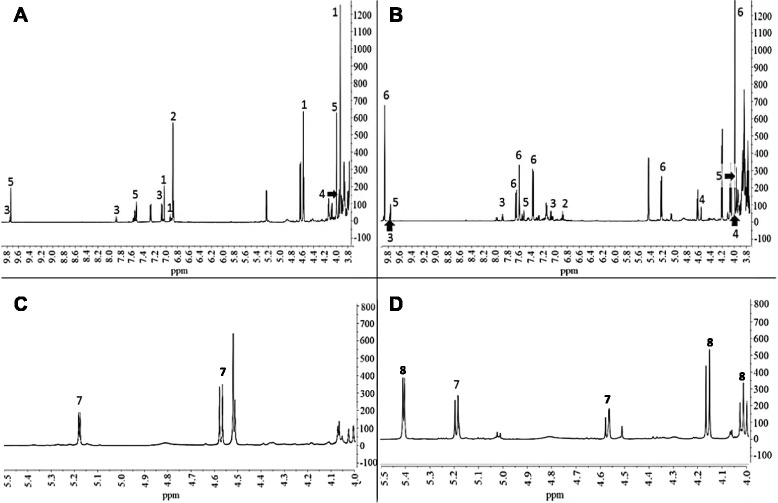
^1^H NMR spectra (methanol- *d*4-KH_2_PO_4_ in D_2_O, pH 6.0 extract) of medium on which *P. microspora* was cultured (Medium 1) and of the control. (**a**) spectrum of Medium 1 in the range δ 3.7–9.9. (**b**) spectrum of the control media in the range δ 3.7–9.9. (**c**) spectrum of Medium 1 in the range δ 4.1–5.5 (carbohydrates range). (**d**) spectrum of the control medium in the range δ 4.1–5.5 (carbohydrates). The assigned peaks are as follows, 1: vanillyl alcohol; 2: *p*-hydroxybenzoic acid; 3: *p*-hydroxybenzaldehyde; 4: vanillic acid; 5: vanillin; 6: glucovanillin; 7: glucose and 8: sucrose

In all the experiments conducted, the same biotransformation medium containing grind green pod material was used. However, different fungi were cultured on this common media. Before any further investigations can be pursued into the biotransformation abilities of the fungi with respect to flavor compounds, it is essential to confirm the identity of the metabolites that had been biotransformed and the new products that are formed. In order to identify the products in the medium after 30 days of fungal growth, two approaches were adopted. The first approach consisted in identifying flavor related metabolites and sugars present in green pods by comparison of the NMR spectra of medium extracts against the NMR spectra of reference compounds. In this way in the control medium made of green pods as well as in the spent medium, 8 molecules of interest were identified (vanillyl alcohol, *p*-hydroxybenzoic acid, *p*-hydroxybenzaldehyde, vanillic acid, vanillin, glucovanillin, glucose and sucrose) (Fig. 5 (a, b, c and d) which shows the ^1^H NMR spectra of the medium on which *P. microspora*, as an example, was cultured, as well as the control).

**Fig. 6:**
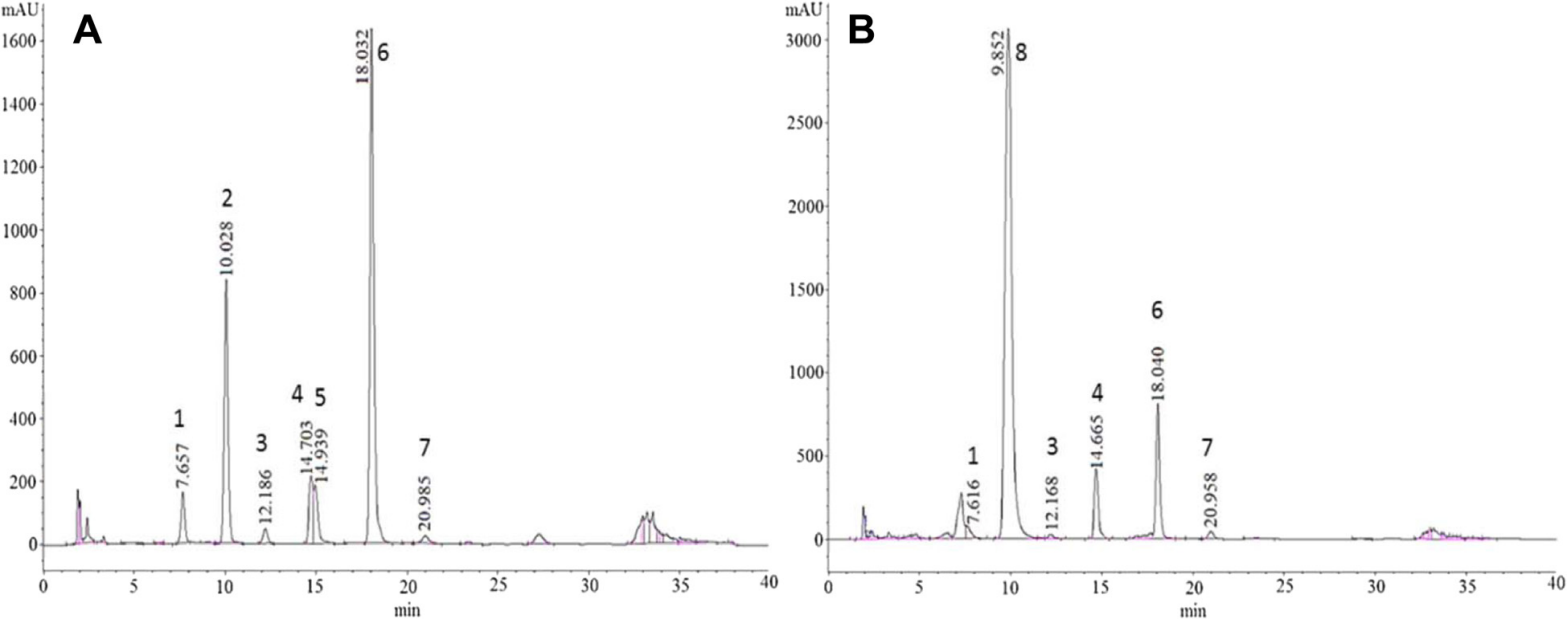
HPLC profile with a retention time range of 0 to 40 min. (**a**) spectra of medium on which *P. microspora* was cultured (Medium MOTU10); (**b**) spectra of the control medium. The assigned peaks are as follows, 1: *p*-hydroxybenzyl alcohol; 2: vanillyl alcohol; 3: *p*-hydroxybenzoic acid; 4: *p*-hydroxybenzaldehyde; 5: vanillic acid; 6: vanillin; 7: *p*-coumaric acid (detected in HPLC only) and 8: glucovanillin. The retention times are shown next to each peak

The second approach was to perform an HPLC analysis on the same samples so as to confirm the identity of the compounds found in the ^1^H NMR analysis (Fig. 6 (a and b)).

Additionally, *p*-coumaric acid was identified by HPLC, but not by ^1^H NMR due to the higher sensitivity of HPLC to detect compounds present at a lower concentration of detection than NMR can detect (*p*-coumaric acid concentration: 0.214 mmol/L of medium in the control and 0.156 mmol/L of medium in the spent medium on which *P. microspora* was cultured). Based on the peak heights, sucrose disappeared completely from the medium while the level of glucose increased (Fig. 5 (c and d)).

### Comparing the biotransformation reactions from fungi

**Fig. 7:**
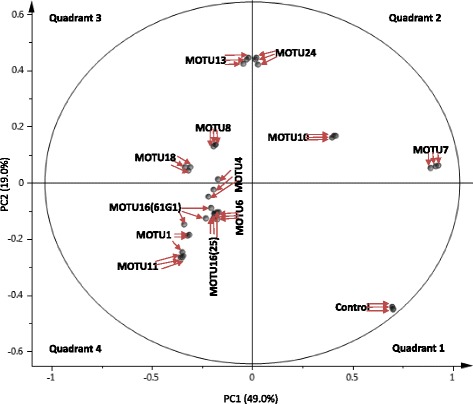
Scatter Score Plot of principal component (PC) 1 and 2 of the principal component analysis (PCA) results obtained from ^1^H NMR spectral data of the pod based media on which fungi were cultured and scaled to Pareto distribution. Twelve fungi were used for culture, additionally a control was included: MOTU1: *Fusarium proliferatum*, MOTU4: *Acremonium implicatum*, MOTU6: *Phomopsis phyllanthicola*, MOTU7: *Diaporthe phaseolorum*, MOTU8: *Nemania bipapillata*, MOTU10: *Pestalotiopsis microspora*, MOTU11: *Colletotrichum gloeosporioides*, MOTU13: *Nigrospora* sp, MOTU16-25: *Botryosphaeria ribis*, MOTU16-61G1: *Botryosphaeria ribis*, MOTU18: *Mycosphaerella marksii* and MOTU24: *Fusarium oxysporum*.)

The scatter score plot of the principal component analysis (PCA) of the ^1^H NMR spectral data (data matrix, Additional file 2: Table S3) of the pod based media shows that metabolites present from the control medium (green pod media only without any fungal culture initiated) places it alone in quadrant 1 relative to the experimental samples. This implies that there were significant differences between metabolites present in the control compared to the experiments where 12 fungi were cultured individually on the same media made of green pod material (Fig. 7).

^1^H NMR spectral data from the media on which *D. phaseolorum* (MOTU7), *P. microspora* (MOTU10) and *F. oxysporum* (MOTU24) were cultured, shows that they cluster together in quadrant 2. Whereas the spectral data for the media on which *Nigrospora* sp. (MOTU13), *N. bipapillata* (MOTU8) and *M. marksii* (MOTU18) were cultured, shows that they cluster together in quadrant 3. Finally the spectral data for the media on which *A. implicatum* (MOTU4), *B. ribis* (MOTU16 - 61G1), *C. gloeosporioides* (MOTU11), *F. proliferatum* (MOTU1), *B. ribis* (MOTU16 - 25) and *P. phyllanthicola* (MOTU6) were cultured, shows that they cluster together in quadrant 4. The different fungi did not cluster based on their order or genus, for instance despite belonging to the same genus and based on metabolite composition medium on which *F. oxysporum* (MOTU24) was cultured occurs in quadrant 2 whereas medium on which *F. proliferatum* (MOTU1) was cultured occurs in quadrant 4. This is so despite quadrants 2 and 4 being antagonistic in terms of metabolites i.e. metabolites which are present in a higher concentration in quadrant 2 would be in a lower concentration in quadrant 4 and vice versa. The results thus show that it is not possible to predict the biotransformation abilities of the fungus based on the order and genus that they belonged to and that such empirical data from experiments are important to decipher the connection between specific endophytes and flavor development in vanilla pods. This thus renders building a hypothesis from literature for potential fungal endophytes with specific effects on flavor compounds difficult. Furthermore, *F. oxysporum* (MOTU24) is a pathogen [24] which was introduced in this work so as to compare the differences in biotransformation abilities of a pathogen from vanilla compared to endophytes from the same plant and on the same green pod based medium.

**Fig. 8:**
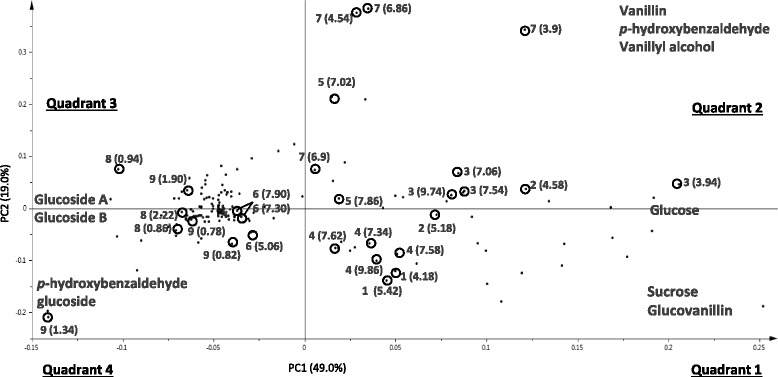
Scatter Loading Plot of principal component (PC) one and two of the principal component analysis (PCA) results obtained from ^1^H NMR spectral data of the pod based media on which fungi were cultured and scaled to Pareto distribution. Chemical shifts identified for each metabolite are shown in bracket in the figure; the chemical shifts from literature (all from [10] except those for Vanillyl alcohol which is from [26]) are shown in brackets in this figure legend, as follows, next to the numbers (one to nine) that identify the metabolites in the above figure: 1 – Sucrose (Chemical shifts: δ 5.4, δ 4.17), 2 – Glucose (Chemical shifts: δ 5.18, δ 4.57), 3 – Vanillin (Chemical shifts: δ 9.73, δ 7.52, δ 7.49, δ 7.04, δ 3.93), 4 – Glucovanillin (Chemical shifts: δ 9.82, δ 7.62, δ 7.57, δ 7.34, δ 5.19, δ 3.95), 5 - *p*-hydroxybenzaldehyde (Chemical shifts: δ 9.75, δ 7.85, δ 7.04), 6 - *p*-hydroxybenzaldehyde glucoside (Chemical shifts: δ 9.84, δ 7.94, δ 7.29, δ 5.01), 7 - Vanillyl alcohol (Chemical shifts: δ 3.75, δ 4.38, δ 5.02, δ 6.71, δ 6.72. δ 6.89, δ 8.79), 8 - bis[4-(β-D-glucopyranosyloxy)-benzyl]-2-isopropyltartrate (Glucoside A) (Chemical shifts: δ 2.20, δ 0.92, δ 0.86) and 9 - bis[4-(β-D-glucopyranosyloxy)-benzyl]-2-(2-butyl)tartrate (Glucoside B) (Chemical shifts: δ 1.90, δ 1.35, δ 1.10, δ 0.84, δ 0.77). Quadrants were labelled with metabolite(s) that predominate in amount in each

It is essential to know which metabolites contribute significantly to separate the fungi in the PCA score plots as well as finding the relationship between such metabolites. This would then form an indirect method of assessing the differences in biotransformation abilities of the fungi, in terms of metabolites converted and products formed. Particularly it is necessary to find whether such metabolites that demarcate the fungi on the PCA score plot in Fig. 7 are flavor related molecules in vanilla. In order to elucidate the identity of such molecules, a scatter loading plot was constructed based on the PCA score plot results from the ^1^H NMR analysis of medium on which endophytic fungi as well as a pathogen (MOTU24 *F. oxysporum*) was cultured (Fig. 8).

In terms of events, the fungal endophytes isolated from mature green pods are present before the time of pod curing. The very presence of such endophytes in green pods implies that the fungi are all able to feed onto pod material while being unaffected by the antimicrobial properties of *V. planifolia* [25]. The metabolites vanillin, *p*-hydroxybenzaldehyde and vanillyl alcohol contribute to the final dark pod vanilla flavor. The scatter loading plot in Fig. 8 shows that all three metabolites occur in quadrant 2 only. Hence media on which fungi *D. phaseolorum* (MOTU7), *P. microspora* (MOTU10) and *F. oxysporum* (MOTU24) were cultured also clustered in quadrant 2 on the PCA (Fig. 7). The three aforementioned fungi are thus associated with a higher concentration of vanillin, *p*-hydroxybenzaldehyde and vanillyl alcohol essential to vanilla flavor compared to the control and to all other fungi tested in this part of the work. Although within the green pod, the three aforementioned molecules do not occur freely and rather occur in glycosylated form, it is possible to imagine a situation where the fungi would affect the relative ratios at which the three flavor related molecules would occur in the pod prior to curing. However, flavor related phenolics occur in glycosylated form in the mature green pods in order to render them less toxic. Despite the results here do not show this, it is possible that the biotransformed molecules would be again glycosylated by the green pod living plant material but this time, the ratio at which such molecules occur in the green pod would differ due to the biotransformation intervention of the fungi. However, this later hypothesis can be checked with further research. If quadrant 2 harbors those fungi that could potentially have an effect on vanilla flavor (Fig. 7), the antagonistic fungi to those in quadrant 2 (based on the final metabolites present in the medium after the experiment) are those in quadrant 4 i.e. *A. implicatum* (MOTU4), *B. ribis* (MOTU16 - 61G1), *C. gloeosporioides* (MOTU11), *F. proliferatum* (MOTU1), *B. ribis* (MOTU16 - 25) and *P. phyllanthicola* (MOTU6)*.* Given that the aforementioned fungi are antagonistic to those (*D. phaseolorum* (MOTU7), *P. microspora* (MOTU10) and *F. oxysporum* (MOTU24)) that influence the quality of the green pods with regards to vanilla flavor, it is possible that controlling fungi present in quadrant 4 (Fig. 7) through the application of targeted fungicides might improve the quality of the green pods. Further research in this direction may be conducted. In extreme cases of infection, the pathogenic fungus *F. oxysporum* (MOTU24) kills vanilla plants. However, the results in the PCA score plot (Fig. 7) show that the medium on which this fungus was grown occurs in quadrant 2 in Fig. 7, which is the quadrant associated with a major amount of three vanilla flavor related compounds in the experimental medium (Fig. 8). This implies that *F. oxysporum* (MOTU24) has the ability to influence the amount of flavor present in green pods. This would then affect the amount of flavor metabolites in the mature green pods that would be available for post-harvest curing. It is to be noted that the fungal endophytes used in this work were isolated from such mature green pods.

Fungi need a carbon source to grow and develop and can use various sources. However, they vary in their ability to utilize different carbon sources and thus show some form of adaptation to their environment. For instance, different fungal taxa target different carbon sources [26]. However, not all fungi tested consume the same type and amount of carbon sources. For instance, quadrant 3 on the scatter loading plot (Fig. 8) consists of those fungi that consume sucrose and glucose as carbon sources. Such fungi include *Nigrospora* sp. (MOTU13 *N. bipapillata* (MOTU8) and *M. marksii* (MOTU18) according to the scatter score plot in Fig. 7. Additionally quadrant 4 on the scatter loading plot (Fig. 8) is characterized by those fungi that consumed vanillin as a carbon source and, according to the scatter score plot (Fig. 7), include fungi *A. implicatum* (MOTU4), *B. ribis* (MOTU16 - 61G1), *C. gloeosporioides* (MOTU11), *F. proliferatum* (MOTU1), *B. ribis* (MOTU16 - 25) and *P. phyllanthicola* (MOTU6).

Le Comité d’experts FAO/OMS sur les additifs alimentaires [27] states that the organoleptic property of vanillyl alcohol is defined as balsamic, vanilla-like flavor. Although vanillyl alcohol is not the only molecule in vanilla to have an organoleptic balsamic flavor, an increase in its amount would certainly contribute to this flavor. Furthermore, gas chromatography-olfactometry (GC-O) analysis shows that although vanillyl alcohol is present in pods at a much lower concentration than vanillin, its contribution to aroma is as intense [28]. According to Ranadive [4], the Bourbon type vanilla, which includes pods from Réunion Island, is characterized by an intense balsamic flavor. The presence of high amounts of vanillyl alcohol in the media (Fig. 7 and 8) on which *D. phaseolorum* (MOTU7), *P. microspora* (MOTU10) and *F. oxysporum* (MOTU24) were cultured is hence of interest with regards to vanilla flavor. This may then contribute to the intense balsamic flavor which is characteristic of Bourbon type vanilla.

**Fig. 9:**
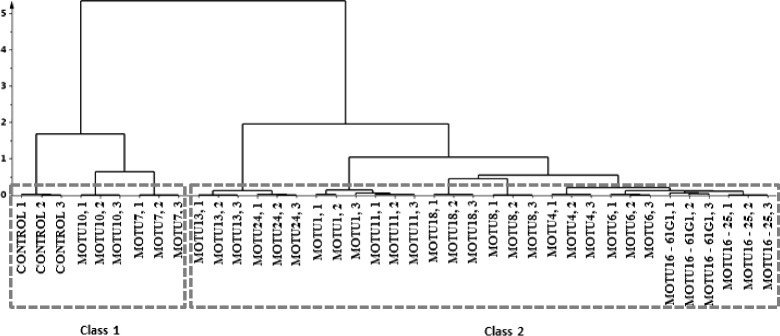
Dendrogram showing the clustering in the ^1^H NMR spectral data of the green pod based media on which fungi were cultured. Twelve fungi were used for culture, additionally a control was included: **Class 1** - CONTROL, 1, 2, 3: Control; MOTU10, 1, 2, 3: *Pestalotiopsis microspore*; MOTU7, 1, 2, 3: *Diaporthe phaseolorum*; **Class 2 -** MOTU13, 1, 2, 3: *Nigrospora* sp.; MOTU24, 1, 2, 3: *Fusarium oxysporum*; MOTU1, 1, 2, 3: *Fusarium proliferatum*; MOTU11, 1, 2, 3: *Colletotrichum gloeosporioides*; MOTU18, 1, 2, 3: *Mycosphaerella marksii*; MOTU8, 1, 2, 3: *Nemania bipapillata*; MOTU4, 1, 2, 3: *Acremonium implicatum*; MOTU6, 1, 2, 3: *Phomopsis phyllanthicola*; MOTU16 - 61G1, 1, 2, 3: *Botryosphaeria ribis*; MOTU16 – 25, 1, 2, 3: *Botryosphaeria ribis*

Given that ^1^H NMR analysis of the media on which the fungi tested in this work did not cluster based on the fungal order or genus, it is possible to generate a dendrogram to find the clustering in terms of the metabolites in the medium after the experiments and hence based on the biotransformative abilities of the fungi tested (Fig. 9).

The dendrogram shows 2 classes with the control medium clustering in class 1 together with the medium on which *P. microspora* (MOTU10) and *D. phaseolorum* (MOTU7) were cultured but not *F. oxysporum* (MOTU24) which occurs in class 2. This is so, despite the fact that the metabolites in the medium on which *P. microspora* (MOTU10), *D. phaseolorum* (MOTU7) and *F. oxysporum* (MOTU24) were sufficiently similar for them to occur in the same quadrant 2 in the principal component analysis (PCA) (Fig. 7). The reason for this is that spent media on which the pathogen *F. oxysporum* (MOTU24) was cultured occurs close to *Nigrospora* sp. in Fig. 7 i.e. the pathogen occurs close to the borders of quadrant 2 and quadrant 3. The dendrogram (Fig. 9) suggests that the biotransformation reactions of *P. microspora* (MOTU10) and of *D. phaseolorum* (MOTU7) (found in class 1) are sufficiently different from those of other fungi tested in this work (found in another class 2) with regards to vanilla aroma that further research on their actions on flavor is necessary such as the effects of different incubation times as well as characterizing the volatiles produced by the fungi.

In order to validate the dendrogram model, a permutation test (Additional file 1: Figure S1) and the *p-value* of the Analysis of variance from cross-validated residuals (P CV-ANOVA) (Additional file 1: Table S2) were calculated. The supervised dendrogram model generated (Fig. 9) with 39 samples (13 parameters with 3 replicates each) was validated through a permutation test that calculates the goodness of fit and the predictive ability of the model, represented by R2 and Q2, respectively. R2 describes how well the data in the training set are mathematically reproducible and its value can vary from 0 to 1, where 1 means a model has a perfect fit. The R2Y value obtained was 0.999 implying a high reproducibility. If Q2 has a value of more than 0.5, the model is considered to have good predictability and if it is higher than 0.9 and less than 1.0, then the model is considered to have an excellent predictability. The Q2Y value obtained was 0.976 hence the model has an excellent predictability. With a minimum of 5 partial least squares (PLS) components, the model generally has excellent reproducibility. 20 components were used hence the model has excellent reproducibility. The regression of Q2 lines intersect at below zero (Y-value = −2.43) with all Q2 values of permuted Y vectors being lower than the original which also confirms the validity of the model. Also, given the P-value is less than 0.05 (value is 1.41 × 10^−4^ in Additional file 1: Table S2), the dendrogram class clustering model is significant.

### Amounts of biotransformed flavor metabolites by fungi grown on the same media

Catabolism of the compounds present in the same green pod based media by each fungus alters the amounts of flavor related metabolites present in the medium, in which the mature green pod material was the only nutrient for the fungus. In order to quantify the bioconversion by a selection of the tested fungi, calculations on the ^1^H NMR analysis of metabolites from the media (Table 4) were performed.

**Table 4:** Average amounts with standard deviation of different forms of vanillin and related compounds in media as well as carbohydrates on which *Pestalotipsis microspora* (Medium MOTU10); *Diaporthe phaseolorum* (Medium MOTU7); *Fusarium oxysporum* (Medium MOTU24); *Nemania bipapillata* (Medium MOTU8); *Fusarium proliferatum* (Medium MOTU1) were cultured for 30 days

Quadrant in PCA in which media occur	1	2	2	2	3	4
Metabolite/ Medium	Control	MOTU10	MOTU7	MOTU24	MOTU8	MOTU1
Vanillin	(17.0 ± 0.6) X 10^−3^	(37.0 ± 1.61) X 10^−3^	(56.0 ± 11.2) X 10^−3^	(1.9 ± 0.5) X 10^−3^	(1.4 ± 2.4) X 10^−3^	0
Glucovanillin	(127.0 ± 2.1) X 10^−3^	0	0	(0.9 ± 0.2) X 10^−3^	0	0
*p*-hydroxybenzaldehyde	(6.2 ± 0.7) × 10^−3^	(3.9 ± 0.5) × 10^−3^	(9.0 ± 1.7) × 10^−3^	(1.2 ± 0.3) × 10^−3^	0	0
Vanillyl alcohol	(15.5 ± 7.0) × 10^−3^	(100.0 ± 6.3) × 10^−3^	(38.6 ± 6.6) × 10^−3^	(11.7 ± 2.6) × 10^−3^	(7.7 ± 5.5) × 10^−3^	(3.1 ± 1.2) × 10^−3^
Vanillic acid	0	(9.2 ± 6.1) × 10^−3^	(7.1 ± 2.7) X 10^−3^	(1.0 ± 0.2) × 10^−3^	(1.9 ± 1.5) × 10^−3^	(2.9 ± 2.6) × 10^−3^
*p*-hydroxybenzoic acid	(7.4 ± 0.9) × 10^−3^	(87.9 ± 12.7) × 10^−3^	(16.5 ± 4.3) × 10^−3^	(13.3 ± 2.5) × 10^−3^	(17.1 ± 12.1) × 10^−3^	(1.0 ± 1.7) × 10^−3^
Sucrose	350.5 ± 82.7	0	0	0	0	0
Glucose	54.3 ± 7.3	143.5 ± 8.7	0	6.6 ± 2.1	23.1 ± 19.5	43.1 ± 17.6
Fructose	0	118.9 ± 2.5	279.9 ± 75.3	0	0	0

The quadrant in which each medium occur in the Principal Component Analysis (PCA) (Fig. 7 and Fig. 8) is also indicated below. The quantification was performed through ^1^H NMR (Micromoles per Gram DW green pods, n = 3 biological replicates)

Six media, including the control, were chosen on which the fungi MOTU10 *P. microspora*, MOTU7 *D. phaseolorum,* MOTU24 *F. oxysporum*, MOTU8 *N. bipapillata*, MOTU1 *F. proliferatum* were cultured. The choice was made based on the PCA score plot results (Fig. 7) where at least one representative media from each quadrant was chosen, in order to have a quantitative overview of metabolites that characterize each quadrant. However, all media in quadrant 2 (Fig. 7) were selected given that media in this quadrant were clustered together based on the presence of high amounts of vanilla flavor related metabolites according to the scatter loading plot (Fig. 8). The quadrant from which the chosen media belongs to on the PCA score plot is indicated in Table 4 (Quadrant in PCA in which media occurs: 1, 2, 3 and 4). Quantification on ^1^H NMR data was performed for nine metabolites: six compounds related to vanilla flavor that were identified by ^1^H NMR (Vanillin, Glucovanillin, *p*-hydroxybenzaldehyde, Vanillyl alcohol, Vanillic acid, *p*-hydroxybenzoic acid) as well as three carbohydrates (Sucrose, Glucose and Fructose) all of which separate the samples onto the different quadrants on the PCA score plot (Fig. 7).

The amounts of the selected nine metabolites present in the medium differed depending on the fungus that was grown on that medium (Table 4). There was no fructose in the spent media on which *F. oxysporum* (Medium MOTU24) and *F. proliferatum* (Medium MOTU1) were cultured. It is possible that the fructose was consumed by the fungi given that *Fusarium* spp., such as *F. oxysporum* f.sp. cubense, consume fructose [29]. However, the amount of fructose in the spent Medium MOTU7 on which *D. phaseolorum* was cultured is almost that of the amount of sucrose in the control Medium. Given that sucrose was completely consumed, the fructose may thus have come from the hydrolysis of sucrose. Depending on the length of time of culture, the fungi would deplete the media completely of sugars as was the case in this work. The amounts of the flavor related metabolites vanillin, vanillyl alcohol, vanillic acid and *p*-hydroxybenzoic acid increased in the medium on which *P. microspora* (Medium MOTU10) was cultured compared to the control. Whereas in Medium MOTU7 (cultured with *D. phaseolorum*), the amounts of vanillin, vanillic acid, *p*-hydroxybenzoic acid, but not vanillyl alcohol, were higher than in the control medium. The amounts of vanillin in Medium MOTU24 (on which the vanilla pathogen *F. oxysporum* was cultured) as well as that in Medium MOTU8 (on which *N. bipapillata* was cultured) were lower than that of the control. Both Media MOTU8 and MOTU1 (on which *F. proliferatum* was cultured) had lower amounts of flavor related metabolites compared to the control with the exception of vanillic acid and *p*-hydroxybenzoic acid which were higher. Overall, the quantity of flavor related metabolites in the medium tend to decrease from medium that are placed in quadrant 2 (highest quantity, Table 4), moving to lower amounts in quadrant 3 and quadrant 4 (lowest quantity, Table 4). Medium MOTU7 shows the highest vanillin amount whereas Medium MOTU10 shows the highest vanillyl alcohol amount. Hence, in Medium MOTU10, most of the vanillin glucoside was converted into vanillyl alcohol, by *P. microspora*, which increased more than 6 fold relative to the control. Vanillin increased more than two fold. But the overall picture is that in the mass balance only a small amount of about 10 % vanillin is lost through the bioconversion by *P. microspora*, whereas with *F. proliferatum* (Medium MOTU1) a 100 % loss of vanillin was observed. Sucrose was completely consumed by all fungi tested whereas glucose amount increased in Medium MOTU10. Overall, from the ^1^H NMR analysis, it was observed that all fungi tested catabolized either all or almost all (as in the medium MOTU24) glucovanillin present in the media without an increase in vanillin content i.e. the amount of vanillin in the experiments were lower than that in the control except in the case of MOTU10 *P. microspora* and of MOTU7 *D. phaseolorum*, where the medium showed an increase in vanillin amount in the experimental medium compared to the amount present in the control.

As a consequence, not all fungi contribute equally to a change in vanilla flavor in tests conducted under laboratory conditions. It is thus conceivable that some fungi would improve the aromatic quality of the pods while others would decrease. Moreover, the different fungi tested would interact together in a concerted manner in the pod. However, the latter situation is beyond the scope of the experiments carried out in this work but could prove as a potential future work conducted on aseptic living green pods and thus possibly by-pass the time consuming curing process. The experiments conducted here concern dead aseptic green pod material.

### Ratios of quality marker metabolites after biotransformation

In view of evaluating the quality of the material obtained after *P. microspora* (Medium MOTU10); *D. phaseolorum* (Medium MOTU7); *F. oxysporum* (Medium MOTU24); *N. bipapillata* (Medium MOTU8) and MOTU1 *F. proliferatum* (Medium MOTU1) cultures, a comparison is necessary to reference parameters that define quality in cured vanilla pods, the final product of vanilla production. A method of evaluating quality involves a calculation based on the ratios in which the four quality markers (*p*-hydroxybenzoic acid (*p*-HB acid), p-hydroxybenzaldehyde (*p*-HBAld), vanillic acid and vanillin) occur in cured pods [4]. Relative to the ratios in the control Medium, not all fungal biotransformations produced the same ratios R1 (Vanillin/p-HBAld), R2 (Vanillic acid/p-HBAld), R3 (p-HB acid/p-HBAld), R4 (Vanillin/Vanillic acid) and R5 (Vanillin/p-HB acid) (Table 5) where p-HBAld is *p*-hydroxybenzaldehyde and p-HB acid is *p*-hydroxybenzoic acid. Medium MOTU10 had the closest ratio R1 to the reference, Medium MOTU7 and 3 to that of R2 and Medium MOTU7 to that of R4. However the ratios R3 and R5 for Medium MOTU10, MOTU7, MOTU24, MOTU8 and MOTU1 are well outside the acceptable reference range for good quality cured beans.

**Table 5:** Ratios of various vanilla compounds related to quality in the control and in the spent medium on which MOTU10 *Pestalotipsis microspora*; MOTU7 *Diaporthe phaseolorum*; MOTU24 *Fusarium oxysporum*; MOTU8 *Nemania bipapillata* and MOTU1 *Fusarium proliferatum* were cultured for 30 days

Sample	R1 = Vanillin/p-HBAld	R2 = Vanillic acid/p-HBAld	R3 = p-HB acid/p-HBAld	R4 = Vanillin/Vanillic acid	R5 = Vanillin/p-HB acid
Control	2.73	-	1.19	-	2.30
Medium MOTU10	9.54	2.37	22.82	4.02	0.42
Medium MOTU7	6.24	0.78	1.83	7.98	3.42
Medium MOTU24	1.57	0.83	11.07	1.89	0.14
Medium MOTU8	-	-	-	0.74	0.08
Medium MOTU1	-	-	-	-	-
Reference ratios (cured pods, [4])	10–20	0.53–1.5	0.15–0.35	12–29	40–110

The ratios associated to quality in dark pods are indicated where p-HBAld: *p*-hydroxybenzaldehyde; p-HB acid: *p*-hydroxybenzoic acid. The values from Medium MOTU10, MOTU7, MOTU24, MOTU8 and MOTU1 which are either within the range of closest to the range of the references are underlined and in bold

At this stage it is not yet possible to state how the knowledge on the endophytes bioconversion abilities can be applied to improve the curing process but it is possible to speculate one potential application. The fungi tested had an effect on the ratios of different flavor related compounds as they occur in the green pod based media. The results suggest potential for further work of varying different parameters e.g. incubation time, relative humidity and temperature and to then re-assess the reference ratios as in Table 5. This approach could potentially give a fungus or a group of fungi which, either on their own or in conjunction, could convert green pod material into a high quality dark pod flavor containing material. This process can then significantly decrease costs and the time for the final product to be ready by by-passing the curing process. It is also essential to isolate endophytes after scalding and during the curing process and to find their biotransformation abilities.

## Conclusion

The most important results of this study are: [7] Fungal endophytes are present inside *V. planifolia* plants and pods, [22]. The species of fungal endophytes varies depending on the geographical region of isolation, [31]. The method of cultivation of the plants affect the type of endophyte present, as in the case of plants grown under shade house conditions where more endophytes were isolated. Out of twelve isolated fungal endophytes and one pathogen from mature pods which were studied for their ability to *in vitro* convert vanilla flavor related metabolites in green pod material, only *P. microspora* and *D. phaseolorum* caused the formation of a significant additional vanillin and some other related compounds from glucovanillin. *Pestalotiopsis microspora* increased vanillyl alcohol levels the most among all tested fungi in green pod material which may be of importance to Bourbon vanilla.

From these studies it is clear that endophytes might play a major role for the quality of cured vanilla beans. But what we report here should be considered as the top of the iceberg. Much more studies are needed. The localization of vanillin glucosides in the pods is well known; as such conducting further studies to localize the endophytes within pods are also important. The present study is focused on the potential effect of endophytes in the living plant, but further research may investigate the effects of endophytes after harvesting, in the period before scalding, after scalding, etc. with regards to which endophytes are still present and to find their bioconversion capabilities. Also in that context, time and the physical-chemical environment (temperature, pH, water content, etc.) are important variables that need to be explored. Given that all fungi consumed glucovanillin from the medium after 30 days of culture, it is possible that for a much shorter time of culture intermediate bioconversion products would be obtained. Thus the effects of different cultural time frames on biotransformation products can be further investigated. The challenge will be to further increase our understanding of the role of endophytes on vanilla flavor and translate this knowledge to improve cultivation and processing methods.

## Materials and Methods

### Sample collections

To characterize the mode of transmission of the fungi, 15 ovaries with both closed and opened flowers were collected at St. André. To assess fungal diversity and distribution *in planta* and across regions, 15 leaves at ranks 1 (the youngest in the sample set), 3, 5, 10 and 15 (the oldest in the sample set) and 8 months old pods (post pollination) on the plant, were harvested from 7 vanilla producing regions across Réunion Island (St. André, St. Anne, St. Rose, Bois Blanc, Takamaka, Mare longue and Basse vallée). One fragment was retained per pod and per leaf. To gain insight into endosymbiont community differences in undergrowth and shade house grown plants, pods and leaves material collections were made at St. André and St. Anne including plants grown under both conditions. For greater statistical precision, a sample size of fifteen replicates was taken per assessed parameter.

### Fungal endophyte and pathogen isolation

Within 3 h after harvest, collected organs were first washed under running tap water for 15 min. The washed organs were then dipped for 10 s in 95 % alcohol and thoroughly flamed on all sides for 3 s. The latter step was adapted from a protocol of endophyte isolation in *Theobroma cacao* pods by Crozier et al. [30]. To find if the surface of the organ was thoroughly sterilized and that any fungi obtained afterwards originate from inside the organ, the sterilized organ was then touched onto the surface of sterile potato dextrose agar (PDA, Conda-Pronadisa, Madrid, Spain) using sterile forceps. The organ was then cut into small cylindrical pieces and placed onto water agar (15 g Plant Agar, Duchefa Biochemie, Haarlem, The Netherlands, in 1 L distilled water) and incubated at 25 °C. Petri dishes were checked regularly for growth of fungi for up to 4 weeks. Fungi that grew from the sterilized organ pieces were transferred on PDA, taking actively growing hyphal tips. Single-spore cultures were prepared for sporulating fungi to ensure the purity of the fungus. A strongly diluted spore suspension was prepared and smeared on a malt agar plate to allow single colonies to develop. Isolates have been deposited at the Fungal Culture Collection of the Muséum National d’Histoire Naturelle (Paris). In the case of the pathogen *Fusarium oxysporum* f.sp. *vanillae*, the same procedure was adopted except that isolation was carried from an infected root organ.

### DNA extraction, PCR and sequencing

Fungi were grown on Malt Extract Agar (MA) for 4 weeks. Morphotypes were categorized based on macroscopic and microscopic cultural characteristics. Fungal mycelium was collected from representatives of each morphotype grown on MA and genomic deoxyribonucleic acid (DNA) was extracted using the QIAGEN DNeasy Plant Mini Kit (Qiagen, Hilden, Germany) as per manufacturer’s instructions. Polymerase chain reaction (PCR) was performed to amplify ribosomal deoxyribonucleic acid (rDNA) regions in a total volume of 25 μl, with 12.5 μl (50–100 ng) of DNA template, 0.625 units of Taq DNA polymerase (Q-BIOgene, Illkirch, France), 5 μl of PCR buffer, 0.5 μl of 25 mM dNTPs (Eurogentec, Seraing, Belgium) and 1 μl of each 10 μM primer per reaction tube. The primer sets ITS 4/ITS 5 [31] and LROR/LR6 (Vilgalys, Duke University, Durham, North Carolina, United States) were used to amplify respectively ITS rDNA and the 5’ end of the 28S rDNA from a set of fungi. The elongation factor (EF) oligonucleotide primer set EF-1H and EF-2 T from O’Donnell et al. [32] was used to amplify a 700 bp portion of the EF-1α gene from *Fusarium* spp. Amplifications were performed on a BioRad DNA engine thermal cycler with the following parameters: a 4 min step at 94 °C, followed by 30 cycles (10 cycles for β-tubulin primer sets) of 30 s at 94 °C, 30 s at an annealing temperature of either 55 °C (for ITS4/ITS5, EF-1H/EF-2 T and β-tubulin primer sets) or 50 °C (for LROR/LR6 primers) and 40 s at 72 °C and then a final 10 min extension step at 72 °C. Additionally, for β-tubulin primer sets only: Denaturation at 94 °C (15 s), annealing at 45–65 °C (30 s), extension at 72 °C (48 s for first round and an additional 3 s for every additional round, for a total of 20 rounds) followed by 72 °C for 6 min. PCR products were purified and then sequenced by the Genoscope (Évry, France), on both strands to confirm the accuracy of each sequence. The DNA sequences were assembled using CodonCode Aligner v. 3.7.1. (CodonCode Corporation), checked by visual inspection and edited if necessary. Sequences were deposited in GenBank (http://www.ncbi.nlm.nih.gov/genbank/).

### Taxonomic composition and phylogenetic analysis of endophytes

Fungal MOTUs (Molecular Organizational Taxonomic Units) were identified using internal transcribed spacer (ITS) sequences. In cases where a more detailed identification at the species level was not possible with ITS sequences, elongation factor (EF-1α) sequences (*Fusarium* spp. isolates), β-tubulin (*Aspergillus niger*) and 28S sequences were used with the BLAST option at http://blast.ncbi.nlm.nih.gov/Blast.cgi. The best hits were carefully examined to attribute species names. Large-subunit ribosomal RNA gene (LSU rDNA) sequences have been used for phylogenetic analysis. Sequences were then aligned using ClustalW on MEGA software version 5.10 [33]. Alignments included reference sequences from NCBI (www.ncbi.nlm.nih.gov) and the final alignments were edited manually. Using the maximum likelihood tree construction method with the Tamura-Nei model on MEGA, a phylogenetic tree was then constructed from 28S sequences. The number of bootstrap replications used was 1500. The tree was rooted with *Cunninghamella blakesleeana* (MOTU23).

### Cultivation of endophytes and one pathogen for metabolomics analysis

Twelve isolated fungi and one pathogen were cultured each one separately but on the same media type made from mature green pods in order to allow comparison of results. The control was green pod medium only without any fungal culture initiated. In order to make a homogenous media preparation, all media required for the experiments in this work were made on the same day and mixed thoroughly before being autoclaved and distributed in petri-dishes. Three experiments were run as replicates per fungi. 8 months post-pollination pods were freeze-dried and crushed into a fine powder. Fifteen grams of dried powdered pod material were added to 15 g of agar (Duchefa Biochemie, Haarlem, The Netherlands) in 1 L of distilled water. The cultures were incubated at 28 ± 1 °C for 30 days at 45 % relative humidity. The fungi were then scrapped off the surface of the media, and the remaining media were freeze-dried.

### ^1^H NMR procedures and quantification

A ^1^H NMR analysis was performed onto the dried material. The pod-based media was freeze-dried and ground into a fine powder. To 50 mg of the material, 1.5 mL of KH_2_PO_4_ buffer (pH 6.0) in D_2_O containing 0.05 % trimethylsilylpropionic acid sodium salt (TMSP, w/w) and methanol-*d*_*4*_ (1:1) was added. The mixture was vortexed at room temperature for 1 min, ultrasonicated for 15 min, and centrifuged at 13,000 rpm for 10 min. An aliquot of 0.8 mL was used for NMR analysis. ^1^H NMR spectra were recorded at 25 °C on a 600 MHz Bruker AV 600 spectrometer equipped with cryo-probe operating at a proton NMR frequency of 600.13 MHz. CD_3_OD-d_4_ was used as the internal lock. Each ^1^H NMR spectrum consisted of 64 scans requiring 5 min and 26 s acquisition time. All NMR parameters were the same as those used by Kim et al. [34]. The resulting spectra were manually phased and baseline-corrected and calibrated to TMSP at 0.0 ppm, using MestReNova software (v. 8.0.2, Mestrelab Research S.L). Compounds were identified based on results of previous studies or from reference compound measurements [10, 35, 36]. The ^1^H NMR spectra were automatically reduced to ASCII file. Spectral intensities were scaled to total intensity and reduced to integrated regions of equal width (0.04) corresponding to the region of δ 0.30–10.02. The regions of δ 4.70–5.00 and δ 3.28–3.40 were excluded from the analysis because of the residual signal of H_2_O and CD_3_OD, respectively. Bucketing was performed by MestReNova software with scaling on total intensity. A principal component analysis (PCA) and dendrogram were constructed with SIMCA-P software (v. 13.0, Umetrics, Umeå, Sweden) using scaling based on Pareto method.

The amounts of metabolites were calculated using the ^1^H NMR equation as in Turczan and Medwick [37] as follows: nx = ny (Ix / Iy) (Ny / Nx) where nx : Number of moles of unknown; ny : Number of moles of TMSP; Nx : Number of protons of unknown; Ny : Number of protons of TMSP; Ix : Integration of peak of unknown and Iy : Integration of peak of TMSP (9 protons, 2.90 × 10^−4^ mol). The assigned chemical shifts of the single peaks integrated for quantification by ^1^H NMR for each metabolite are vanillin – δ 9.73 (s), glucovanillin – δ 9.82 (s), *p*-hydroxybenzaldehyde – δ 9.75 (s), vanillyl alcohol – δ 3.87 (s), vanillic acid – δ 3.89 (s), *p*-hydroxybenzoic acid – δ 6.90 (d, *J* = 8.8 Hz), sucrose – δ 5.40 (d, *J* = 3.6 Hz), glucose (two peaks integrated) – δ 4.63 (d, *J* = 8.0 Hz), δ 5.18 (d, J = 3.7 Hz) and fructose - δ4.03 (H-8, dd, J = 12.9,1.0 Hz) [10, 36, 37].

### HPLC-DAD procedures

Freeze-dried pod-based media (50 mg) was transferred into a 2 ml microtube. A volume of 1.5 ml of MeOH-Water (1:1) was added to the samples. The mixture was vortexed at room temperature for 1 min, ultrasonicated for 20 min and centrifuged at 13,000 rpm for 10 min. The supernantant was then analyzed with HPLC. The HPLC system employed was an Agilent CPL 1100 series (Massy, France) equipped with LC Chemstation software, degasser G1322A, binary pump G1312A, autosampler G1313A, thermostated column oven G1316A and diode array detection system G1315B to monitor at all wavelengths from 200 to 400 nm. For the column, a LiChrospher 100 RP-18 (250 × 4.6 mm i.d., s-5, 5 μm) (Merck, Darmstadt, Germany), protected with a guard column LichroCART 4–4 (Merck), was used at 35 °C. Gradient elution was performed with solution A, composed of 90 % water at 0.1 % acetic acid (pH 3.3) and 10 % methanol, and solution B, comprising 70 % methanol, delivered at a flow rate of 1.0 mL/min as follows: initially 70 % of solution A up to 15 min; 67 % A as from 29 min; 0 % A as from 30 min up to 34.9 min and finally 100 % A as from 35 to 40 min. The injection volume for the extract was 30 μl. The wavelength of detection was set at 280 nm. A library with the high-performance liquid chromatography (HPLC) retention times and ultraviolet diode array detector (UV-DAD) spectra was made with 8 compounds (*p*-hydroxybenzyl alcohol, *p*-hydroxybenzoic acid, *p*-hydroxybenzaldehyde, vanillyl alcohol, vanillin, vanillic acid, glucovanillin and *p*-coumaric acid).

### Availability of supporting data

For each MOTU, the 28S ribosomal RNA sequences was submitted to the GenBank Repository. GenBank assigned an accession number for each sequence. The accession number and the corresponding MOTU is shown as follows: MOTU1: KR349521, MOTU2: KR349522, MOTU3: KR349523, MOTU4: KR349524, MOTU5: KR349525, MOTU6: KR349526, MOTU7: KR349527, MOTU8: KR349528, MOTU9: KR349529, MOTU10: KR349530, MOTU11: KR349531, MOTU12: KR349532, MOTU13: KR349533, MOTU14: KR349534, MOTU15: KR349535, MOTU16: KR349536, MOTU17: KR349537, MOTU18: KR349538, MOTU19: KR349539, MOTU20: KR349540, MOTU21: KR349541, MOTU22: KR349542, MOTU23: KR349543. The phylogenetic data was deposited in the Dryad Digital Repository and available at: http://dx.doi.org/10.5061/dryad.doi:10.5061/dryad.m5c7m

## Additional files

Additional file 1: Table S1. Identification of endophytes MOTUs based on NCBI BLAST of 28S rDNA, ITS rDNA, EF-1α or β-tubulin sequences. **Figure S1.** Response of permutation test. Correlation of Y-permutated vs. Y-original permutation test of the partial least squares discriminant analysis (PLS-DA) model of results obtained from ^1^H NMR spectral data of the pod based media on which fungi were cultured and scaled to Pareto distribution. The number of permutations used was 20 and the number of components was 20. The R2 value was 0.999 and the Q2 value was 0.976. Additional file 2: Table S2. Statistical validations on SIMCA-P for the PLS-DA model generated. The P-value from the CV-ANOVA is shown and tests the significance of the PLS-DA model.

## Abbreviations

NMR: Nuclear magnetic resonance
^1^H NMR: Proton nuclear magnetic resonance
HPLC: High-performance liquid chromatography
HPLC-DAD: High-performance liquid chromatography - diode array detector
MOTU: Molecular operational taxonomic unit
LSU rDNA: Large-subunit ribosomal RNA gene
NGS: Next-generation sequencing
RH: Relative humidity
PDA: Potato dextrose agar
PCA: Principal component analysis
GC-O: Gas chromatography-olfactometry
P CV-ANOVA: *p-value* of the Analysis of variance from cross-validated residuals
PLS: Partial least squares
p-HBAld: p-hydroxybenzaldehyde
p-HB acid: p-hydroxybenzoic acid
PCR: Polymerase chain reaction
DNA: Deoxyribonucleic acid
rDNA: Ribosomal deoxyribonucleic acid
ITS: Internal transcribed spacer
