# Supplementary material for: Fungal endophytes of Vanilla planifolia across Réunion Island: isolation, distribution and biotransformation
Source: BMC Plant Biol. 2015 Jun 14;15:142. doi: 10.1186/s12870-015-0522-5 (PMC4465486; doi:10.1186/s12870-015-0522-5)
Supplement: Additional file 1: Table S1. — Identification of endophytes MOTUs based on NCBI BLAST of 28S rDNA, ITS rDNA, EF-1α or β-tubulin sequences. Figure S1. Response of permutation test. Correlation of Y-permutated vs. Y-original permutation test of the partial least squares discriminant analysis (PLS-DA) model of results obtained from 1H NMR spectral data of the pod based media on which fungi were cultured and scaled to Pareto distribution. The number of permutations used was 20 and the number of components was 20. The R2 value was 0.999 and the Q2 value was 0.976. [file 12870_2015_522_MOESM1_ESM.docx]

**Table S1**

Identification of endophytes MOTUs based on NCBI BLAST of 28S, ITS rDNA, EF-1α or β-tubulin sequences.

| **MOTU number** | **Isolate**  **number** | **DNA region** | **Blast best match : Genbank accession number and ID** | **Class**  **Order** |
| --- | --- | --- | --- | --- |
| MOTU 1 | 9B (LCP5974) | EF-1α | >gi\|149798252\|gb\|EF453149.1\| Gibberella intermedia strain NRRL 43666 (Fusarium proliferatum) | Sordariomycetes  Hyprocreales |
| MOTU 2 | 82D1 | ITS | >gi\|262476602\|gb\|GQ505743.1\| Fusarium scirpi strain NRRL 36478 | Sordariomycetes  Hypocreales |
| MOTU 3 | 28 | EF-1α | >gi\|306412978\|gb\|HM347120.1\| Fusarium oxysporum strain NRRL 26360 | Sordariomycetes  Hypocreales |
| MOTU 4 | 29A (LCP5979) | ITS | >gi\|316980277\|emb\|FN706553.1\| Acremonium implicatum MUCL 1412 | Sordariomycetes  Hypocreales |
| MOTU 5 | 3C1B (LCP5984) | 28S | >gi\|523713894\|gb\|KC157757.1\| Purpureocillium lilacinum strain M4076 | Sordariomycetes  Hypocreales |
| MOTU 6 | 55E (LCP5980) | ITS | >gi\|215490348\|gb\|FJ441623.1\| Phomopsis phyllanthicola strain msy55 | Sordariomycetes  Diaporthales |
| MOTU 7 | 5 (LCP5978) | ITS | >gi\|283856804\|gb\|GU066686.1\| Diaporthe phaseolorum isolate 123AC/T (Phomopsis sp) | Sordariomycetes  Diaporthales |
| MOTU 8 | 61B (LCP5982) | ITS | >gi\|44893890\|gb\|AY541610.1\| Nemania bipapillata strain CL8 | Sordariomycetes  Xylariales |
| MOTU 9 | 9B | ITS | >gi\|387773616\|gb\|JQ846066.1\| Xylaria sp. 5485 | Sordariomycetes  Xylariales |
| MOTU 10 | 61F (LCP5983)  55A (LCP6051) | ITS | >gi\|21310048\|gb\|AF377292.1\| Pestalotiopsis microspora strain CBS364.54 | Sordariomycetes  Xylariales |
| MOTU 11 | 69D (LCP5988) | ITS | >gi\|169135011\|gb\|EU482214.1\| Colletotrichum gloeosporioides ICMP 17323 | Sordariomycetes  Glomerellaceae |
| MOTU 12 | 39 | ITS | >gi\|82799468\|gb\|DQ286216.1\| Colletotrichum sp. | Sordariomycetes  Glomerellaceae |
| MOTU 13 | S101Z1 (LCP5987)  42a,b,c | ITS | >gi\|317383391\|gb\|HQ631070.1\| Nigrospora sp. TMS-2011 voucher SC9d1p7-1 | Sordariomycetes  Trichosphaeriales |
| MOTU 14 | 69H (LCP5985)  S104Z1 | ITS | >gi\|383842765\|gb\|JQ316443.1\| Fungal endophyte isolate EL-10 Australia | Sordariomycetes  Trichosphaeriales |
| MOTU 15 | S104Z1 Bis | 28S | >gi\|290889519\|gb\|GU390656.1\| Delitschia chaetomioides strain SMH 3253.2 | Dothideomycetes  Pleosporales |
| MOTU 16 | 25 (LCP6048)  61d,e,g, S104Z1ter | ITS | >gi\|34328662\|gb\|AY236935.1\| Botryosphaeria ribis isolate CMW7772 | Dothideomycetes  Botryosphaeriales |
| MOTU 17 | 51B (LCP5998) | ITS | >gi\|330369659\|gb\|JF261465.1\| Guignardia mangiferae strain CPC18848 (CBS128856 T) | Dothideomycetes  Botryosphaeriales |
| MOTU 18 | 33B (LCP5989) | ITS | >gi\|262386897\|gb\|GQ852747.1\| Mycosphaerella marksii strain CPC:13273 | Dothideomycetes  Capnodiales |
| MOTU 19 | 8B2 (LCP6049) | ITS | >gi\|310769695\|gb\|GU944569.1\| Penicillium citrinum strain CBS 13945 | Eurotiomycetes  Eurotiales |
| MOTU 20 | S102Z1 (LCP6050) | β-tub | >gi\|110743536\|dbj\|AB248059.1\| Aspergillus fumigatus strain: IAM 13869 | Eurotiomycetes  Eurotiales |
| MOTU 21 | 74A (LCP5981) | ITS | >gi\|22023786\|gb\|AF485074.1\| Sarcosomataceous endophyte E99297 strain E99297 | Pezizomycetes  Pezizales |
| MOTU 22 | 51A (LCP5976) | 28S | >gi\|353703687\|gb\|HQ848487.1\| Perenniporia nanlingensis voucher Cui 7589 | Agaricomycetes  Polyporales |
| MOTU 23 | 3C1A (LCP5977) | ITS | >gi\|15147895\|gb\|AF254932.1\| Cunninghamella blakesleana strain CBS 133.27 | Zygomycetes  Mucorales |

**Table S2**

Statistical validations on SIMCA-P for the PLS-DA model generated. The P-value from the CV-ANOVA is shown and tests the significance of the PLS-DA model.

| **MVDA method** | **R^2^Y** | **Q^2^Y** | **P CV-ANOVA** |
| --- | --- | --- | --- |
| PLS-DA | 1.00 | 0.99 | 1.41 x 10^-4^ |

.
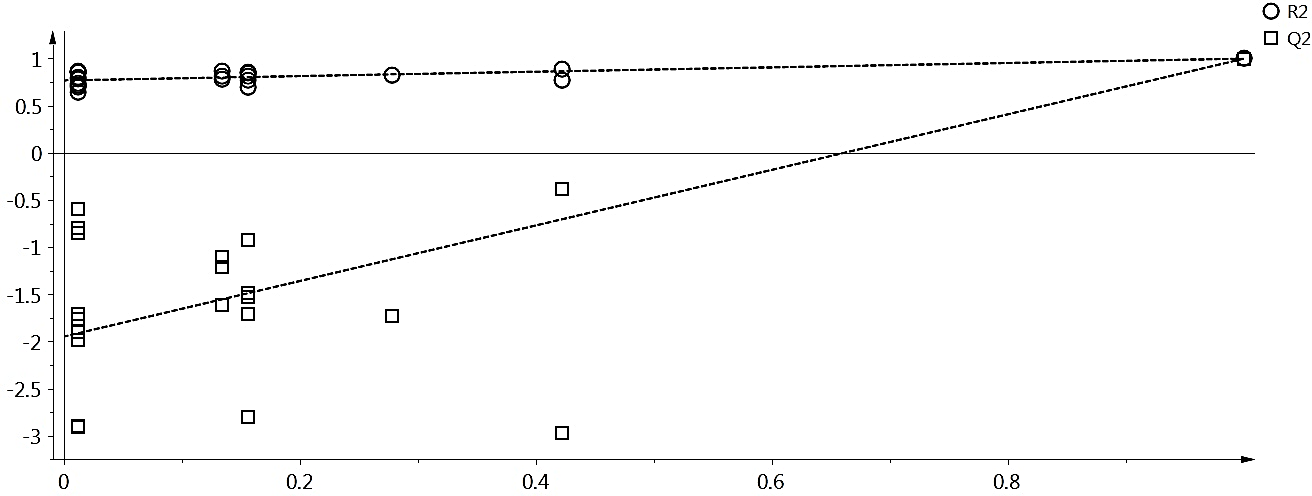


**Fig.S1.** Response of permutation test. Correlation of Y-permutated vs. Y-original permutation test of the partial least squares discriminant analysis (PLS-DA) model of results obtained from ^1^H NMR spectral data of the pod based media on which fungi were cultured and scaled to Pareto distribution. The number of permutations used was 20 and the number of components was 20. The R2 value was 0.999 and the Q2 value was 0.976.
