# Supplementary material for: Fungal endophytes of Vanilla planifolia across Réunion Island: isolation, distribution and biotransformation
Source: BMC Plant Biol. 2015 Jun 14;15:142. doi: 10.1186/s12870-015-0522-5 (PMC4465486; doi:10.1186/s12870-015-0522-5)
Supplement: Additional file 2: Table S2. — Statistical validations on SIMCA-P for the PLS-DA model generated. The P-value from the CV-ANOVA is shown and tests the significance of the PLS-DA model. [file 12870_2015_522_MOESM2_ESM.pdf]

Table S3

Data sheet for MVA of 1H NMR spectral data of pod based media on which fungi were cultured

CHEMICAL SHIFT	ppm	ppm	ppm	ppm	ppm
**FUNGI**	**0.38**	**0.42**	**0.46**	**0.5**	**0.54**
**Nigrospora sp 1**	0.002791	0.002796	0.002796	0.002813	0.00282669
**Nigrospora sp 2**	0.002911	0.002923	0.002929	0.002951	0.00295838
**Nigrospora sp 3**	0.002796	0.002801	0.002809	0.002825	0.00282707
**Nemania bipapillata 1**	0.003103	0.003131	0.003158	0.003209	0.00326274
**Nemania bipapillata 2**	0.00329	0.003331	0.00337	0.003416	0.00348151
**Nemania bipapillata 3**	0.003214	0.003253	0.003293	0.003343	0.00340973
**Fusarium proliferatum 1**	0.003702	0.003724	0.003765	0.003802	0.00385146
**Fusarium proliferatum 2**	0.003321	0.003349	0.003379	0.003419	0.00345465
**Fusarium proliferatum 3**	0.003663	0.00369	0.003717	0.003762	0.00379911
**Fusarium oxysporum f.sp. vanillae 1**	0.002726	0.002725	0.002741	0.002753	0.00276981
**Fusarium oxysporum f.sp. vanillae 2**	0.002704	0.002723	0.002726	0.002741	0.00276621
**Fusarium oxysporum f.sp. vanillae 3**	0.002765	0.002757	0.002775	0.002795	0.00281079
**Phomopsis phyllanticola 1**	0.003159	0.003181	0.003191	0.003213	0.0032383
**Phomopsis phyllanticola 2**	0.003125	0.003151	0.00316	0.003197	0.00322309
**Phomopsis phyllanticola 3**	0.003058	0.003078	0.003102	0.003119	0.00314323
**Colletotrichum gloeosporioides 1**	0.003626	0.003653	0.003685	0.003714	0.00375669
**Colletotrichum gloeosporioides 2**	0.003573	0.003608	0.00364	0.003673	0.00372428
**Colletotrichum gloeosporioides 3**	0.003485	0.00352	0.003561	0.003588	0.00362613
**Acremonium implicatum 1**	0.003116	0.003137	0.003152	0.003166	0.00318484
**Acremonium implicatum 2**	0.002979	0.002992	0.003012	0.003037	0.00305308
**Acremonium implicatum 3**	0.002972	0.002979	0.002997	0.003011	0.00302769
**Diaporthe phaseolorum 1**	0.001859	0.001862	0.001875	0.001875	0.00187813
**Diaporthe phaseolorum 2**	0.001924	0.001922	0.001926	0.00193	0.00193634
**Diaporthe phaseolorum 3**	0.001879	0.001885	0.001889	0.001898	0.00190199
**Pestalotiopsis microspora 1**	0.002307	0.002314	0.002327	0.002328	0.00233542
**Pestalotiopsis microspora 2**	0.002263	0.002279	0.002285	0.002288	0.00228496
**Pestalotiopsis microspora 3**	0.002368	0.002379	0.00238	0.002387	0.00239317
**Mycosphaerella marksii 1**	0.003395	0.003409	0.003424	0.003435	0.00345555
**Mycosphaerella marksii 2**	0.003646	0.003669	0.003691	0.003711	0.00373277
**Mycosphaerella marksii 3**	0.003388	0.003396	0.003409	0.003424	0.00343583
**Botryosphaeria ribis_61G1 1**	0.00336	0.003374	0.003394	0.003411	0.00343656

**Botryosphaeria ribis_61G1 2**	0.003588	0.003614	0.003635	0.003653	0.00370164
**Botryosphaeria ribis_61G1 3**	0.003568	0.003586	0.00362	0.003659	0.00369762
**Botryosphaeria ribis_25 1**	0.003187	0.003206	0.003226	0.003234	0.00325964
**Botryosphaeria ribis_25 2**	0.003209	0.003219	0.003241	0.00326	0.00328974
**Botryosphaeria ribis_25 3**	0.00324	0.00326	0.003281	0.003306	0.00333286
**Control 1**	0.001998	0.002005	0.001999	0.002008	0.00201333
**Control 2**	0.002027	0.002043	0.002041	0.002051	0.00205309
**Control 3**	0.002082	0.002077	0.002089	0.002099	0.00209782

ppm 0.58	ppm 0.62	ppm 0.66	ppm 0.7	ppm 0.74	ppm 0.78	ppm 0.82	ppm 0.86	ppm 0.9
0.002843	0.00287	0.002891	0.00292	0.002958	0.003051	0.003201	0.003667	0.008643
0.002978	0.003009	0.003041	0.003089	0.003135	0.003244	0.003431	0.003982	0.008765
0.002849	0.002883	0.002901	0.002938	0.002982	0.003085	0.003245	0.003747	0.008733
0.00335	0.003423	0.003563	0.003632	0.003851	0.004142	0.00478	0.005795	0.011964
0.003579	0.003656	0.003815	0.003912	0.004156	0.004476	0.005134	0.006226	0.012303
0.003499	0.003585	0.003735	0.00384	0.004076	0.004396	0.005064	0.00617	0.012348
0.00391	0.003965	0.004052	0.00417	0.004297	0.004513	0.004966	0.006578	0.011175
0.003508	0.003557	0.003632	0.003734	0.003854	0.004031	0.004468	0.00602	0.011738
0.003846	0.003907	0.003988	0.00409	0.004211	0.004414	0.004835	0.006346	0.0108
0.002799	0.002819	0.002857	0.002903	0.00295	0.003079	0.00336	0.003965	0.009608
0.002772	0.002804	0.002838	0.00289	0.002946	0.003069	0.003341	0.003921	0.009717
0.002832	0.002865	0.002899	0.002942	0.003002	0.003136	0.003423	0.004044	0.009789
0.003272	0.003316	0.003367	0.003433	0.003506	0.003668	0.003999	0.004626	0.010051
0.003273	0.00331	0.003367	0.003427	0.003527	0.003692	0.004066	0.004788	0.010759
0.003179	0.003225	0.003269	0.003331	0.003413	0.00357	0.003915	0.004595	0.01041
0.003806	0.003857	0.00393	0.004032	0.004143	0.004346	0.004753	0.00581	0.009543
0.003762	0.003822	0.003898	0.003987	0.004113	0.004301	0.004723	0.005808	0.009997
0.003677	0.003729	0.003801	0.003909	0.004024	0.004206	0.004596	0.005536	0.009611
0.003205	0.003245	0.003284	0.003339	0.003399	0.003552	0.003853	0.004646	0.011509
0.003086	0.003127	0.003181	0.003248	0.003317	0.003475	0.003833	0.004818	0.012119
0.003051	0.00309	0.003134	0.003194	0.003262	0.003407	0.003732	0.004633	0.011701
0.001885	0.001889	0.001897	0.001908	0.001931	0.001993	0.00207	0.002411	0.005834
0.001942	0.001952	0.001959	0.001975	0.001996	0.002052	0.002134	0.002494	0.005814
0.001905	0.001905	0.001919	0.001935	0.001953	0.002002	0.00208	0.002406	0.005803
0.002348	0.00235	0.002369	0.002395	0.002429	0.002496	0.002603	0.003026	0.007207
0.002303	0.002309	0.002318	0.002345	0.002373	0.002437	0.002551	0.002966	0.007238
0.002406	0.002419	0.002433	0.00246	0.002495	0.002578	0.002716	0.003191	0.007277
0.003466	0.003501	0.003529	0.00358	0.003641	0.003783	0.004012	0.004757	0.011092
0.003771	0.003807	0.003847	0.003913	0.004006	0.00417	0.004459	0.005346	0.011533
0.003449	0.003472	0.003498	0.003541	0.003589	0.00371	0.00391	0.004575	0.010677
0.003472	0.003507	0.003546	0.003619	0.003689	0.003822	0.00409	0.004746	0.009998

0.003739	0.003788	0.003838	0.003938	0.004035	0.004203	0.00454	0.005419	0.010527
0.003747	0.003807	0.003871	0.003982	0.004099	0.004293	0.004679	0.005654	0.011596
0.0033	0.003351	0.003369	0.003437	0.003509	0.003649	0.003924	0.004637	0.010616
0.003309	0.003372	0.003393	0.003458	0.003534	0.003668	0.003942	0.004663	0.010661
0.003371	0.003423	0.003456	0.00353	0.0036	0.003749	0.004041	0.004819	0.010884
0.002021	0.002029	0.002047	0.002095	0.002135	0.002296	0.004858	0.003562	0.005101
0.002063	0.002074	0.00209	0.002159	0.002197	0.00239	0.004986	0.003642	0.005173
0.00211	0.002124	0.002141	0.002208	0.002247	0.002453	0.005024	0.003696	0.005132

ppm 0.94	ppm 0.98	ppm 1.02	ppm 1.06	ppm 1.1	ppm 1.14	ppm 1.18	ppm 1.22	ppm 1.26
0.012797	0.007904	0.003567	0.003002	0.003581	0.004557	0.004818	0.003814	0.005872
0.012519	0.007827	0.003569	0.003054	0.003632	0.004575	0.004878	0.003892	0.005961
0.012781	0.007891	0.003507	0.00298	0.003568	0.004571	0.004833	0.00381	0.005908
0.015481	0.012501	0.007303	0.005929	0.005918	0.008191	0.006597	0.00949	0.012571
0.015446	0.012408	0.00706	0.005912	0.006014	0.008232	0.006674	0.009605	0.012495
0.015736	0.012637	0.007243	0.005945	0.006009	0.00828	0.00672	0.009672	0.012653
0.009607	0.00464	0.006103	0.005219	0.005597	0.005054	0.005886	0.006833	0.009682
0.013698	0.004743	0.005802	0.005212	0.005412	0.004991	0.005871	0.007146	0.010344
0.009586	0.004536	0.005919	0.005114	0.005446	0.004936	0.005709	0.006598	0.009166
0.013584	0.008894	0.003108	0.002959	0.003488	0.007049	0.005073	0.00534	0.006109
0.013839	0.008872	0.003396	0.002956	0.003495	0.00712	0.005226	0.005355	0.006063
0.013806	0.008625	0.003481	0.002982	0.003562	0.007111	0.005262	0.005398	0.006027
0.014864	0.006521	0.005655	0.003354	0.003526	0.003681	0.004141	0.004889	0.007315
0.015642	0.006637	0.005653	0.003428	0.00361	0.003813	0.004355	0.005286	0.008266
0.015307	0.006609	0.005701	0.003421	0.003567	0.00375	0.00426	0.005148	0.008358
0.009038	0.005903	0.004914	0.004836	0.005014	0.005408	0.006418	0.0084	0.014882
0.009841	0.005868	0.004865	0.004806	0.004986	0.005381	0.006394	0.008373	0.015139
0.009531	0.005546	0.004702	0.004667	0.004845	0.005219	0.006176	0.007896	0.013593
0.017189	0.007914	0.006472	0.003548	0.003591	0.003777	0.004196	0.004746	0.008346
0.017599	0.007756	0.00627	0.003523	0.003599	0.003844	0.004393	0.00534	0.010436
0.017439	0.00783	0.006492	0.003545	0.003578	0.003787	0.004299	0.005127	0.009574
0.007897	0.006514	0.002321	0.002329	0.002136	0.002073	0.002214	0.002512	0.003939
0.007839	0.006441	0.002374	0.002362	0.002183	0.002121	0.002259	0.002559	0.003992
0.007857	0.006514	0.002362	0.002353	0.002155	0.002089	0.002224	0.002508	0.003921
0.010062	0.007975	0.002791	0.002769	0.002735	0.002965	0.00306	0.003851	0.00505
0.01015	0.00818	0.00286	0.002762	0.002739	0.00296	0.003071	0.003896	0.005118
0.010011	0.007838	0.002765	0.002771	0.002768	0.002995	0.003094	0.003879	0.005066
0.01567	0.010508	0.003421	0.003437	0.003484	0.00362	0.003852	0.004305	0.006816
0.015661	0.009895	0.003123	0.003464	0.003602	0.003781	0.004062	0.004573	0.007128
0.015088	0.010128	0.003403	0.003467	0.003488	0.003603	0.003825	0.004243	0.006743
0.014522	0.006555	0.005985	0.003627	0.003685	0.003565	0.004131	0.004604	0.006703

0.013403	0.005337	0.006742	0.003981	0.004201	0.00429	0.005061	0.00619	0.010313
0.015412	0.006566	0.006174	0.003884	0.004047	0.004107	0.004927	0.005898	0.009563
0.015675	0.005083	0.007885	0.003215	0.003362	0.003459	0.004078	0.005937	0.008082
0.015852	0.004974	0.008041	0.003238	0.003406	0.003507	0.00414	0.006054	0.008404
0.015611	0.005031	0.007821	0.00324	0.003422	0.003539	0.004203	0.006119	0.008481
0.006035	0.00559	0.002364	0.002228	0.0023	0.002468	0.002385	0.002583	0.003792
0.006049	0.005564	0.002287	0.002224	0.002312	0.002482	0.002404	0.002619	0.004004
0.006032	0.005556	0.002301	0.002262	0.002355	0.002528	0.002444	0.002669	0.003977

ppm 1.3	ppm 1.34	ppm 1.38	ppm 1.42	ppm 1.46	ppm 1.5	ppm 1.54	ppm 1.58	ppm 1.62
0.005521	0.004368	0.003739	0.003671	0.003663	0.004423	0.003786	0.003607	0.003528
0.00558	0.004406	0.003762	0.003691	0.003714	0.004447	0.003818	0.003646	0.003582
0.005526	0.004357	0.003718	0.003649	0.003672	0.004445	0.003788	0.003601	0.003522
0.009271	0.009248	0.005422	0.005937	0.006374	0.005952	0.005675	0.005418	0.005475
0.009174	0.00903	0.005286	0.005859	0.006316	0.005851	0.005613	0.005379	0.005391
0.009277	0.009135	0.005334	0.005895	0.006332	0.005884	0.005617	0.005383	0.00542
0.023016	0.023199	0.009379	0.003881	0.00341	0.003764	0.003754	0.005123	0.005031
0.034841	0.044957	0.016192	0.00525	0.003598	0.003853	0.003754	0.005449	0.006555
0.022665	0.023577	0.009391	0.003867	0.003396	0.003727	0.003699	0.004994	0.005028
0.005615	0.004656	0.003545	0.003468	0.003721	0.004594	0.003173	0.003316	0.003334
0.00569	0.004728	0.003567	0.003454	0.003587	0.004653	0.003205	0.003314	0.003318
0.005599	0.004636	0.003541	0.003473	0.003609	0.00468	0.003254	0.003343	0.003344
0.013856	0.012877	0.006222	0.004204	0.003757	0.004636	0.004035	0.004034	0.004228
0.018299	0.018105	0.007933	0.004464	0.003724	0.004564	0.003971	0.004212	0.004605
0.017646	0.018151	0.008253	0.004671	0.003805	0.004596	0.004017	0.004238	0.00458
0.03171	0.031946	0.016726	0.006953	0.004201	0.004008	0.004075	0.005338	0.005054
0.035166	0.036831	0.01787	0.006841	0.003981	0.003869	0.003981	0.005428	0.005295
0.033039	0.035152	0.016668	0.006423	0.003858	0.003782	0.003803	0.005183	0.005108
0.012092	0.01131	0.006933	0.005108	0.004295	0.005082	0.00455	0.004358	0.004311
0.020435	0.021268	0.010988	0.005961	0.004249	0.004819	0.004435	0.004568	0.004715
0.01698	0.017195	0.009527	0.005786	0.004365	0.004964	0.004547	0.004466	0.004517
0.004081	0.003277	0.002471	0.00245	0.002835	0.00273	0.002249	0.002542	0.002374
0.004169	0.003378	0.002538	0.002495	0.002868	0.002753	0.002281	0.002584	0.002407
0.004104	0.003312	0.002496	0.002458	0.00282	0.002755	0.002257	0.002561	0.002389
0.004992	0.003857	0.003229	0.003342	0.003572	0.003454	0.002953	0.00312	0.003144
0.005263	0.004138	0.003347	0.003408	0.003541	0.003538	0.002989	0.003141	0.003162
0.004988	0.003855	0.003238	0.00334	0.003564	0.003471	0.002968	0.003145	0.003161
0.006538	0.005195	0.00414	0.004198	0.004579	0.004797	0.003783	0.00417	0.003925
0.006561	0.005181	0.004201	0.004327	0.004755	0.00487	0.003914	0.004356	0.004093
0.006465	0.005178	0.004111	0.004162	0.004529	0.00473	0.003751	0.004159	0.003915
0.008487	0.007393	0.004485	0.003819	0.003884	0.004694	0.004525	0.004065	0.003799

0.019698	0.017256	0.007888	0.004242	0.00375	0.004217	0.00449	0.004326	0.004098
0.015715	0.013367	0.00611	0.003922	0.003791	0.004647	0.004548	0.004251	0.004017
0.011347	0.009857	0.004741	0.003739	0.003637	0.004386	0.004615	0.003928	0.003816
0.012303	0.010699	0.005082	0.003819	0.003626	0.004351	0.004653	0.00398	0.003862
0.012027	0.010306	0.004872	0.003768	0.003644	0.004401	0.004639	0.003996	0.003874
0.005694	0.005398	0.003355	0.002714	0.002574	0.002673	0.002561	0.002562	0.002579
0.005445	0.004977	0.003218	0.00269	0.002586	0.002642	0.002555	0.002569	0.002571
0.004862	0.004392	0.003092	0.00272	0.002632	0.002688	0.002597	0.002589	0.002591

ppm	ppm	ppm	ppm	ppm	ppm	ppm	ppm	ppm
**1.66**	**1.7**	**1.74**	**1.78**	**1.82**	**1.86**	**1.9**	**1.94**	**1.98**
0.003349	0.003387	0.003564	0.003451	0.003399	0.004063	0.006236	0.004487	0.004252
0.003412	0.003455	0.003638	0.003528	0.003504	0.004179	0.006216	0.004535	0.004293
0.00335	0.003387	0.003578	0.003455	0.003397	0.004086	0.006255	0.004489	0.004254
0.004564	0.004717	0.005118	0.004759	0.0042	0.005516	0.005977	0.004799	0.004555
0.004522	0.004705	0.005093	0.004735	0.004203	0.005537	0.005923	0.004744	0.004551
0.004512	0.004681	0.005084	0.004739	0.004185	0.005516	0.005972	0.004763	0.00453
0.003757	0.003615	0.003593	0.003766	0.003992	0.004459	0.005782	0.00465	0.004965
0.003823	0.003426	0.003321	0.003553	0.003681	0.004031	0.005373	0.004732	0.006088
0.003724	0.003561	0.003544	0.003694	0.003892	0.004343	0.005607	0.004556	0.00494
0.003173	0.003289	0.00349	0.003502	0.003575	0.004097	0.006204	0.004875	0.00391
0.003163	0.003297	0.003507	0.003521	0.003592	0.004068	0.006369	0.004989	0.003921
0.003201	0.003339	0.003544	0.003565	0.003639	0.004124	0.006416	0.004977	0.003957
0.003695	0.00373	0.00367	0.003762	0.003744	0.004499	0.007448	0.005373	0.004295
0.003673	0.003624	0.003567	0.003668	0.003664	0.004415	0.007314	0.005378	0.004483
0.003714	0.003644	0.003559	0.003644	0.003617	0.004339	0.007137	0.005368	0.004425
0.004096	0.003592	0.003566	0.003674	0.003717	0.003811	0.004269	0.005516	0.005821
0.004048	0.003497	0.003475	0.003617	0.003658	0.003726	0.004204	0.005593	0.006082
0.003867	0.003358	0.003338	0.003475	0.003505	0.003557	0.003976	0.0053	0.005655
0.003959	0.003867	0.00395	0.003984	0.004023	0.004781	0.006469	0.004806	0.004526
0.003898	0.003588	0.003611	0.003669	0.003699	0.004381	0.006026	0.004737	0.00485
0.003898	0.003678	0.003722	0.003753	0.003785	0.00447	0.006196	0.004757	0.004679
0.002231	0.002264	0.002257	0.002322	0.002286	0.002761	0.003288	0.003162	0.002905
0.002269	0.002301	0.002288	0.002352	0.002329	0.002797	0.003291	0.003167	0.002924
0.002245	0.002289	0.002269	0.002334	0.0023	0.002759	0.003307	0.003162	0.002912
0.003235	0.003408	0.003177	0.003201	0.003155	0.003529	0.004425	0.003823	0.003337
0.00327	0.00347	0.003202	0.003236	0.003174	0.003511	0.004488	0.003844	0.003367
0.003249	0.003417	0.003187	0.003211	0.00318	0.003556	0.004435	0.003817	0.00337
0.003819	0.003873	0.003896	0.003973	0.003959	0.004896	0.006551	0.005059	0.006731
0.004015	0.004083	0.004138	0.004219	0.004253	0.005246	0.006734	0.00519	0.006752
0.003792	0.003835	0.00386	0.003917	0.003898	0.004789	0.006332	0.004895	0.006515
0.003865	0.004011	0.004497	0.004035	0.003807	0.004629	0.007147	0.005324	0.003975

0.003811	0.003801	0.004172	0.00389	0.003712	0.004352	0.0064	0.005314	0.004812
0.003936	0.004033	0.004555	0.004092	0.003937	0.00487	0.007444	0.005616	0.004671
0.003925	0.003863	0.004414	0.003933	0.003623	0.004531	0.007381	0.005366	0.003791
0.00393	0.003852	0.004397	0.003941	0.003625	0.004512	0.007388	0.005359	0.003848
0.003952	0.00388	0.004443	0.003957	0.003652	0.00457	0.007404	0.005362	0.003843
0.002375	0.002322	0.00232	0.002345	0.002343	0.002631	0.003746	0.00337	0.003103
0.002376	0.002332	0.002321	0.002346	0.00236	0.00267	0.003778	0.003379	0.003047
0.002424	0.002389	0.002385	0.002408	0.002426	0.00275	0.003849	0.003413	0.003072

ppm 2.02	ppm 2.06	ppm 2.1	ppm 2.14	ppm 2.18	ppm 2.22	ppm 2.26	ppm 2.3	ppm 2.34
0.003848	0.006033	0.004384	0.003944	0.005052	0.003916	0.00377	0.004921	0.00604
0.003949	0.006024	0.004449	0.004028	0.005062	0.003999	0.003873	0.004964	0.006108
0.003852	0.00604	0.004399	0.00394	0.005047	0.003915	0.003769	0.004905	0.006078
0.004248	0.005531	0.004384	0.004515	0.005397	0.004329	0.004413	0.004515	0.004529
0.004257	0.00556	0.00435	0.004558	0.005378	0.004356	0.004435	0.004539	0.004564
0.004226	0.005523	0.004366	0.004498	0.005393	0.004304	0.004402	0.004516	0.004546
0.006313	0.009339	0.005469	0.004386	0.005367	0.004788	0.004972	0.005257	0.005373
0.006741	0.012853	0.006965	0.004168	0.005067	0.004987	0.005663	0.006013	0.00523
0.006108	0.009225	0.005447	0.004282	0.005214	0.004743	0.004913	0.005199	0.005255
0.004058	0.006128	0.004489	0.003974	0.004916	0.00374	0.003651	0.004968	0.005832
0.004073	0.006222	0.004595	0.003959	0.004971	0.003809	0.003648	0.004965	0.006063
0.004122	0.006214	0.004581	0.003995	0.004996	0.003853	0.003704	0.004998	0.006128
0.005157	0.008821	0.005269	0.004318	0.005727	0.004491	0.004578	0.006227	0.007459
0.005531	0.009669	0.005577	0.00425	0.005632	0.004508	0.00483	0.006488	0.007247
0.005559	0.009413	0.005626	0.004267	0.005601	0.004501	0.004814	0.006382	0.00707
0.00744	0.009816	0.006696	0.005055	0.005009	0.004417	0.005015	0.005004	0.004186
0.007735	0.010641	0.006943	0.004919	0.004898	0.004433	0.005197	0.005174	0.004162
0.007123	0.01024	0.006764	0.004764	0.004812	0.004244	0.004953	0.005016	0.004024
0.005127	0.007007	0.005428	0.004818	0.006145	0.004931	0.004897	0.004887	0.005994
0.00588	0.008292	0.00595	0.004578	0.005745	0.004857	0.005086	0.004948	0.005607
0.005564	0.007737	0.005747	0.00467	0.005901	0.004899	0.00496	0.004852	0.005747
0.00275	0.003702	0.003002	0.002835	0.003203	0.00243	0.00264	0.003554	0.00324
0.002793	0.003726	0.003014	0.002863	0.003197	0.002456	0.002684	0.003561	0.003238
0.002773	0.003718	0.003021	0.002835	0.003214	0.002449	0.002668	0.003545	0.003262
0.003388	0.004587	0.003744	0.003487	0.00415	0.003078	0.00339	0.004172	0.004991
0.003427	0.00465	0.003792	0.003487	0.004172	0.003108	0.00346	0.004134	0.005026
0.003429	0.004576	0.003752	0.003512	0.004156	0.003125	0.003446	0.004194	0.004986
0.004347	0.006322	0.004995	0.004599	0.005877	0.004414	0.004515	0.005381	0.006311
0.004545	0.006553	0.005058	0.004821	0.006038	0.004596	0.00479	0.005738	0.006558
0.004245	0.006214	0.00486	0.004503	0.005726	0.004294	0.004433	0.005207	0.006014
0.004597	0.007017	0.00502	0.004838	0.005527	0.004532	0.004173	0.005046	0.006476

0.005725	0.007805	0.005081	0.004536	0.00521	0.004608	0.00443	0.004824	0.00569
0.005613	0.008091	0.005248	0.004967	0.005667	0.004783	0.004545	0.005351	0.006853
0.004358	0.006822	0.004895	0.005076	0.005865	0.004464	0.003921	0.004958	0.006527
0.004434	0.006946	0.004952	0.005064	0.005904	0.004509	0.00396	0.004951	0.006518
0.004449	0.006929	0.004936	0.005102	0.00588	0.004515	0.003964	0.00497	0.006581
0.003004	0.004104	0.003095	0.002698	0.003099	0.002632	0.002687	0.003395	0.003396
0.002984	0.004064	0.003084	0.002714	0.003098	0.002609	0.002706	0.003428	0.003364
0.003011	0.004003	0.003098	0.002786	0.003167	0.00266	0.002763	0.003524	0.003405

**ppm**	**ppm**	**ppm**	**ppm**	**ppm**	**ppm**	**ppm**	**ppm**	**ppm**
**2.38**	**2.42**	**2.46**	**2.5**	**2.54**	**2.58**	**2.62**	**2.66**	**2.7**
0.006683	0.00912	0.008834	0.003873	0.003497	0.003547	0.006179	0.014839	0.004376
0.006728	0.008968	0.008756	0.003952	0.003567	0.003689	0.006306	0.014371	0.004471
0.006723	0.009288	0.008791	0.003843	0.003492	0.003547	0.006228	0.014949	0.004344
0.004958	0.009434	0.008611	0.003569	0.003649	0.003481	0.00447	0.013623	0.003534
0.00503	0.009457	0.008372	0.003489	0.003658	0.003545	0.004593	0.013433	0.003366
0.005012	0.00953	0.008583	0.003485	0.003619	0.003488	0.004539	0.01369	0.003492
0.004858	0.00399	0.003545	0.003621	0.004137	0.004812	0.004041	0.003881	0.004926
0.00458	0.00365	0.003295	0.00329	0.00368	0.004389	0.003706	0.003499	0.004397
0.004789	0.003935	0.003526	0.003554	0.004046	0.004695	0.00396	0.003784	0.004745
0.0067	0.006569	0.005735	0.003409	0.003335	0.003469	0.005753	0.008971	0.004032
0.00694	0.006665	0.005888	0.003445	0.003334	0.003482	0.00587	0.009245	0.004144
0.006936	0.006613	0.005825	0.003479	0.003357	0.003557	0.005961	0.009127	0.004177
0.007898	0.008093	0.006703	0.005611	0.003834	0.003967	0.007724	0.012204	0.005198
0.007723	0.007916	0.006588	0.005437	0.003708	0.003857	0.00753	0.012016	0.005044
0.007561	0.007765	0.006421	0.005457	0.003717	0.003817	0.007315	0.011604	0.005037
0.003915	0.004068	0.003661	0.00351	0.003676	0.003434	0.003465	0.003552	0.003803
0.003815	0.003951	0.00357	0.003428	0.003607	0.003347	0.003382	0.003486	0.003724
0.00371	0.003866	0.003469	0.003322	0.003454	0.003219	0.003234	0.003292	0.003529
0.006358	0.008962	0.007843	0.006117	0.003826	0.003963	0.006366	0.014465	0.005679
0.005824	0.008147	0.007191	0.00566	0.003433	0.003503	0.005628	0.013289	0.005292
0.006011	0.008501	0.007474	0.005884	0.003569	0.003637	0.005856	0.013854	0.00553
0.003258	0.008624	0.004367	0.002445	0.002842	0.003507	0.003561	0.009395	0.003655
0.003261	0.008426	0.004404	0.00247	0.002877	0.0035	0.00354	0.009205	0.003659
0.00329	0.008563	0.004427	0.002466	0.002876	0.00351	0.003555	0.009413	0.003657
0.004395	0.011674	0.005725	0.002918	0.00372	0.003391	0.004245	0.013542	0.003954
0.004518	0.011663	0.006038	0.002932	0.003723	0.003362	0.004236	0.013782	0.004044
0.004539	0.011518	0.005642	0.002932	0.003735	0.003432	0.004362	0.013318	0.003934
0.006717	0.013924	0.009919	0.003706	0.00392	0.004166	0.006485	0.019789	0.004976
0.007107	0.013131	0.01001	0.003703	0.004108	0.004516	0.007058	0.019287	0.004433
0.006417	0.012404	0.010275	0.003733	0.003868	0.004042	0.006136	0.018825	0.004851
0.007019	0.007532	0.00591	0.006478	0.004186	0.004081	0.006829	0.011001	0.005811

0.00599	0.006256	0.004443	0.00587	0.004189	0.003855	0.005891	0.008369	0.005315
0.007124	0.007541	0.005831	0.006458	0.004138	0.00425	0.007178	0.010984	0.0057
0.007016	0.007847	0.005312	0.006857	0.003963	0.003956	0.006865	0.011373	0.005861
0.007032	0.007795	0.005175	0.006873	0.004001	0.003941	0.006848	0.011297	0.005869
0.007059	0.00781	0.005268	0.006855	0.003967	0.003978	0.006915	0.011352	0.005806
0.003707	0.006598	0.00505	0.002512	0.003493	0.00312	0.003522	0.008701	0.003171
0.00367	0.007231	0.004545	0.002458	0.00348	0.003158	0.003529	0.00887	0.003021
0.003802	0.007173	0.00467	0.002551	0.003601	0.003194	0.003655	0.008854	0.003117

ppm 2.74	ppm 2.78	ppm 2.82	ppm 2.86	ppm 2.9	ppm 2.94	ppm 2.98	ppm 3.02	ppm 3.06
0.005368	0.004796	0.007592	0.003003	0.003069	0.003136	0.00644	0.005241	0.003131
0.005287	0.004617	0.007642	0.003059	0.003127	0.003259	0.006441	0.005193	0.003212
0.005331	0.004654	0.007738	0.002971	0.00304	0.003122	0.006447	0.005288	0.00311
0.00356	0.004178	0.007882	0.002919	0.002998	0.003125	0.006151	0.005325	0.003175
0.003544	0.004119	0.007891	0.002882	0.003031	0.0032	0.006177	0.0053	0.003225
0.003511	0.004131	0.00792	0.002885	0.002959	0.003105	0.006162	0.005342	0.003157
0.004715	0.004633	0.004026	0.003453	0.003483	0.003488	0.00354	0.003767	0.003772
0.004368	0.005018	0.004214	0.00312	0.003092	0.00306	0.003097	0.003263	0.003247
0.004617	0.004533	0.003976	0.003386	0.003384	0.003379	0.00341	0.003597	0.003595
0.005547	0.004183	0.005542	0.003127	0.003074	0.003109	0.004427	0.00427	0.003096
0.00563	0.004254	0.005619	0.003095	0.003065	0.003099	0.004495	0.0043	0.003083
0.005603	0.004301	0.005538	0.003097	0.003104	0.003146	0.004542	0.004311	0.003142
0.00619	0.005859	0.00566	0.003029	0.003081	0.00553	0.005106	0.003187	0.003246
0.006128	0.006	0.005667	0.002936	0.002981	0.005296	0.005068	0.003067	0.003124
0.006099	0.005907	0.005648	0.002983	0.002992	0.005257	0.004938	0.003109	0.003142
0.003995	0.004757	0.004272	0.003606	0.003455	0.003468	0.003514	0.003716	0.003726
0.003939	0.004853	0.004328	0.003532	0.003381	0.00339	0.003438	0.003641	0.00365
0.003673	0.004604	0.004151	0.003339	0.003187	0.00317	0.003186	0.003359	0.003351
0.004731	0.006664	0.006654	0.00345	0.00356	0.006353	0.006218	0.003669	0.003649
0.004423	0.006394	0.006422	0.003179	0.003186	0.005654	0.005718	0.003289	0.003229
0.004539	0.006497	0.00654	0.00327	0.003296	0.005943	0.005899	0.003421	0.003358
0.003276	0.004352	0.003587	0.00516	0.00225	0.002133	0.002195	0.004282	0.003754
0.003281	0.004322	0.00356	0.005097	0.002285	0.002151	0.00221	0.004237	0.003727
0.003321	0.004346	0.003852	0.0049	0.00226	0.002148	0.002213	0.004313	0.003743
0.004087	0.004014	0.005262	0.005664	0.002633	0.002568	0.002689	0.005938	0.004749
0.004141	0.003993	0.005366	0.005682	0.002631	0.002557	0.002693	0.006203	0.004594
0.00409	0.003998	0.005289	0.005553	0.002634	0.002598	0.002764	0.005981	0.004621
0.005379	0.004574	0.009672	0.005053	0.003503	0.003556	0.007483	0.007176	0.00351
0.005305	0.004658	0.009995	0.004291	0.003574	0.003788	0.007975	0.00671	0.00358
0.005311	0.004475	0.009156	0.005165	0.003504	0.003533	0.007346	0.006799	0.003504
0.005463	0.0058	0.005685	0.003254	0.003392	0.005721	0.005057	0.003902	0.003604

0.004895	0.00527	0.00498	0.003247	0.003356	0.00518	0.00423	0.003729	0.003496
0.005318	0.005903	0.005449	0.003197	0.003474	0.005914	0.004986	0.003981	0.003721
0.005328	0.006087	0.005215	0.003169	0.003327	0.006146	0.005531	0.003754	0.003345
0.0053	0.00606	0.00522	0.003178	0.003336	0.006121	0.005522	0.003754	0.003351
0.005274	0.006054	0.005224	0.003183	0.003351	0.006167	0.0055	0.003772	0.003362
0.003991	0.004034	0.004049	0.003871	0.002245	0.002221	0.003236	0.00428	0.002246
0.003923	0.004128	0.003972	0.003949	0.002225	0.002217	0.002531	0.005018	0.002253
0.004047	0.004146	0.004053	0.003978	0.002312	0.002321	0.002799	0.004962	0.00235

ppm 3.1	ppm 3.14	ppm 3.18	ppm 3.22	ppm 3.26	ppm 3.42	ppm 3.46	ppm 3.5	ppm 3.54
0.003138	0.003248	0.003782	0.004066	0.004627	0.003599	0.004475	0.005647	0.005531
0.003239	0.003401	0.003945	0.004326	0.005028	0.003522	0.004448	0.005571	0.00553
0.003121	0.003247	0.003782	0.004104	0.004728	0.003564	0.004467	0.005639	0.005545
0.003225	0.003285	0.003387	0.003883	0.004887	0.003589	0.004716	0.005619	0.0057
0.003323	0.003408	0.003551	0.004118	0.005185	0.003444	0.004677	0.005582	0.005686
0.003223	0.003306	0.003418	0.003958	0.004981	0.003502	0.004686	0.005621	0.005734
0.003775	0.003986	0.004077	0.004921	0.006555	0.005718	0.004607	0.004404	0.00502
0.003226	0.003346	0.00338	0.00406	0.005439	0.005508	0.004533	0.004133	0.004621
0.003566	0.003713	0.003763	0.004482	0.005942	0.005702	0.004772	0.004355	0.004886
0.003022	0.003261	0.003373	0.003974	0.005955	0.004909	0.004647	0.006146	0.005279
0.002994	0.003227	0.003336	0.003907	0.005871	0.004837	0.004601	0.006162	0.005231
0.003071	0.003319	0.003447	0.00407	0.006121	0.004901	0.004633	0.006156	0.005297
0.003248	0.004613	0.003404	0.004337	0.004392	0.004589	0.004235	0.004978	0.005228
0.003136	0.004428	0.003275	0.004124	0.004206	0.004393	0.004143	0.004844	0.00509
0.003132	0.004404	0.003275	0.004132	0.004208	0.004534	0.004176	0.004848	0.005081
0.003724	0.00384	0.00401	0.004896	0.005367	0.00408	0.003971	0.004137	0.004726
0.003647	0.003768	0.00393	0.004833	0.005276	0.00398	0.003887	0.004048	0.004645
0.00331	0.003371	0.003463	0.004209	0.004596	0.004379	0.004065	0.004051	0.004543
0.003599	0.003769	0.004012	0.004718	0.00617	0.004228	0.005161	0.006271	0.006754
0.003146	0.003258	0.003424	0.003948	0.005102	0.004031	0.004774	0.005761	0.006186
0.003269	0.003382	0.003557	0.004124	0.005413	0.004202	0.004946	0.005942	0.006374
0.002342	0.002428	0.007224	0.020298	0.002871	0.025707	0.024556	0.012687	0.019397
0.002343	0.002428	0.007038	0.019763	0.002939	0.024965	0.023999	0.01263	0.018862
0.002344	0.002429	0.007184	0.020299	0.002901	0.025655	0.024518	0.012756	0.019344
0.002658	0.002694	0.004926	0.010897	0.003667	0.013246	0.013505	0.009227	0.011974
0.002632	0.002662	0.004705	0.010588	0.003804	0.013075	0.01309	0.00908	0.011715
0.002689	0.002767	0.005043	0.01079	0.003806	0.013188	0.013337	0.009095	0.011792
0.003521	0.003725	0.003759	0.004813	0.005268	0.004341	0.004369	0.005147	0.005091
0.00378	0.004081	0.004256	0.005485	0.006184	0.00379	0.004055	0.004891	0.005002
0.00352	0.003675	0.003643	0.004622	0.00495	0.004483	0.004426	0.005116	0.005053
0.003565	0.003761	0.003669	0.00442	0.005261	0.003464	0.0041	0.004745	0.006786

0.003429	0.003555	0.00351	0.004083	0.004662	0.003291	0.003787	0.004178	0.005641
0.003776	0.004034	0.004097	0.005057	0.006171	0.002984	0.003922	0.004782	0.007015
0.003571	0.003598	0.003308	0.004186	0.005161	0.004035	0.004261	0.004882	0.005449
0.003598	0.003625	0.003336	0.004187	0.005034	0.004006	0.004247	0.004852	0.005384
0.003619	0.003647	0.003372	0.004289	0.005234	0.003949	0.004237	0.004869	0.005457
0.00222	0.002309	0.002988	0.006347	0.002754	0.009356	0.016979	0.017547	0.022831
0.002224	0.002331	0.003061	0.00637	0.002791	0.009311	0.017094	0.018286	0.022278
0.002352	0.002472	0.003234	0.006527	0.002995	0.009607	0.017141	0.01821	0.022387

ppm	ppm	ppm	ppm	ppm	ppm	ppm	ppm	ppm
**3.58**	**3.62**	**3.66**	**3.7**	**3.74**	**3.78**	**3.82**	**3.86**	**3.9**
0.007184	0.008481	0.007666	0.008691	0.011022	0.008537	0.010221	0.010644	0.035628
0.007163	0.008393	0.007706	0.008671	0.010876	0.008525	0.010157	0.011174	0.034256
0.007223	0.008514	0.007745	0.00877	0.011124	0.008574	0.010257	0.010943	0.036022
0.006418	0.008219	0.009457	0.011381	0.014402	0.010597	0.011242	0.012278	0.012271
0.00644	0.008192	0.009573	0.011307	0.014348	0.01039	0.011168	0.01218	0.011817
0.006454	0.008228	0.009465	0.01129	0.014192	0.010533	0.01123	0.012154	0.012318
0.006404	0.009689	0.007417	0.010724	0.013832	0.009442	0.013009	0.014807	0.00824
0.005709	0.008782	0.006792	0.009606	0.012351	0.008911	0.011805	0.013701	0.008073
0.006006	0.009282	0.007088	0.010132	0.012994	0.009399	0.012403	0.014508	0.008544
0.007386	0.009121	0.010098	0.010419	0.0144	0.009075	0.013782	0.014228	0.043966
0.007283	0.008598	0.009858	0.009773	0.013077	0.008635	0.012978	0.013766	0.046099
0.007387	0.008635	0.010011	0.00983	0.013176	0.008717	0.013185	0.014466	0.045247
0.007425	0.007593	0.014047	0.015766	0.02077	0.010607	0.019568	0.017627	0.008276
0.007237	0.007371	0.013579	0.015049	0.019739	0.01035	0.018454	0.016559	0.008065
0.007125	0.007547	0.01344	0.015567	0.020457	0.010708	0.01923	0.017147	0.008254
0.00546	0.007384	0.007619	0.011036	0.012602	0.009426	0.012942	0.011907	0.008619
0.00536	0.007309	0.007506	0.010861	0.012442	0.009266	0.012776	0.011709	0.008518
0.00514	0.00712	0.007215	0.010829	0.012297	0.009718	0.013098	0.012225	0.009366
0.007978	0.009266	0.009443	0.011256	0.014862	0.010871	0.018418	0.012064	0.015218
0.007211	0.008459	0.008422	0.010022	0.013248	0.009827	0.016747	0.011078	0.014881
0.007476	0.008713	0.008852	0.010621	0.014045	0.010381	0.017664	0.011719	0.015032
0.006577	0.006515	0.011334	0.0481	0.032741	0.01239	0.042369	0.024117	0.02512
0.006541	0.006391	0.01113	0.046623	0.032099	0.012417	0.0411	0.023786	0.02448
0.006558	0.006445	0.011239	0.047765	0.032675	0.012393	0.042078	0.024116	0.024962
0.006332	0.007071	0.012159	0.029046	0.02482	0.014155	0.026996	0.021145	0.030763
0.006442	0.007143	0.012208	0.028424	0.025014	0.014326	0.026707	0.021197	0.031327
0.006303	0.007094	0.0122	0.028675	0.0243	0.014071	0.026632	0.02095	0.030179
0.006246	0.00731	0.007705	0.008251	0.010942	0.007471	0.010114	0.009588	0.006857
0.006254	0.00722	0.00774	0.008087	0.010804	0.0072	0.009894	0.009132	0.006277
0.006147	0.007183	0.007544	0.008058	0.010771	0.007323	0.009901	0.009544	0.006777
0.007779	0.00685	0.013548	0.014085	0.016931	0.008661	0.019597	0.023708	0.008898

0.00625	0.00572	0.009591	0.011017	0.012725	0.00695	0.014799	0.017628	0.007785
0.007832	0.00719	0.014274	0.01372	0.016877	0.008473	0.01937	0.022978	0.007205

0.012002	0.007219	0.020555	0.017981	0.018672	0.009393	0.015787	0.015829	0.008445
0.011986	0.0071	0.020197	0.01777	0.01825	0.009128	0.015399	0.015516	0.008228
0.011965	0.007133	0.020521	0.017638	0.018383	0.009035	0.01551	0.015509	0.008182

0.012537	0.02	0.034109	0.012448	0.022542	0.024783	0.052785	0.026778	0.014674
0.012376	0.020434	0.033766	0.012278	0.022929	0.025127	0.052663	0.026209	0.014807
0.01259	0.020439	0.033976	0.012254	0.023098	0.025189	0.052628	0.025754	0.01469

ppm 3.94	ppm 3.98	ppm 4.02	ppm 4.06	ppm 4.1	ppm 4.14	ppm 4.18	ppm 4.22	ppm 4.26
0.013484	0.006302	0.004872	0.00484	0.006333	0.004113	0.003761	0.00445	0.00371
0.012859	0.006121	0.004671	0.004727	0.006137	0.004005	0.003673	0.004319	0.00363
0.013442	0.006258	0.004822	0.004832	0.006352	0.004079	0.00373	0.004418	0.003664
0.009034	0.00655	0.00547	0.005337	0.006437	0.00434	0.004256	0.004639	0.003752
0.008745	0.006314	0.005266	0.005186	0.006255	0.004173	0.004146	0.004504	0.003621
0.008998	0.006505	0.005359	0.005242	0.006279	0.004272	0.004214	0.004543	0.003688
0.006013	0.005032	0.004542	0.005156	0.006874	0.004529	0.004247	0.004663	0.004083
0.005881	0.004922	0.004434	0.004897	0.006559	0.00468	0.004119	0.004427	0.003961
0.006279	0.005269	0.004735	0.005221	0.006833	0.004789	0.004424	0.004783	0.004289
0.00959	0.005601	0.004798	0.004792	0.006482	0.003751	0.003865	0.004616	0.003699
0.00971	0.005523	0.00465	0.004454	0.005577	0.003677	0.003771	0.004405	0.003611
0.009493	0.005374	0.004552	0.004357	0.005395	0.003607	0.003732	0.00435	0.003572
0.008537	0.006828	0.005771	0.00623	0.008155	0.005361	0.004205	0.00461	0.003725
0.008458	0.00673	0.005677	0.006142	0.008148	0.005348	0.004147	0.004561	0.003695
0.008346	0.006743	0.005724	0.006265	0.00848	0.005441	0.004223	0.004641	0.003753
0.005767	0.00552	0.004667	0.005448	0.006979	0.004973	0.004232	0.004408	0.004095
0.005673	0.005423	0.004597	0.005377	0.006964	0.004942	0.004181	0.004332	0.004024
0.006257	0.005908	0.00494	0.005589	0.007248	0.005457	0.004517	0.00464	0.004347
0.009022	0.007574	0.005519	0.004817	0.005597	0.004095	0.00428	0.004611	0.003961
0.008782	0.007441	0.005416	0.004768	0.005496	0.004152	0.004174	0.004445	0.003886
0.008986	0.007592	0.005599	0.004864	0.005574	0.004193	0.004267	0.004568	0.003978
0.038525	0.00631	0.009572	0.009265	0.012095	0.003005	0.002834	0.002974	0.00254
0.037395	0.006722	0.009447	0.009247	0.011944	0.003116	0.002909	0.003036	0.002613
0.037701	0.006468	0.009503	0.009262	0.012056	0.003013	0.002826	0.002964	0.002545
0.01895	0.00634	0.007991	0.007902	0.009416	0.004483	0.003691	0.00423	0.003515
0.018599	0.006703	0.007939	0.007879	0.00935	0.004562	0.003742	0.004264	0.003564
0.01855	0.006309	0.007901	0.007804	0.009227	0.004423	0.00366	0.004167	0.003485
0.006649	0.005576	0.004899	0.005173	0.006009	0.00435	0.004352	0.00563	0.004041
0.006212	0.00515	0.004591	0.004939	0.005712	0.004075	0.004161	0.005387	0.003783
0.006619	0.005597	0.004945	0.005206	0.006075	0.004417	0.004397	0.005647	0.004113
0.007891	0.004886	0.004598	0.005205	0.006988	0.005605	0.004097	0.004501	0.003842

0.006525	0.004567	0.004334	0.004756	0.006039	0.005138	0.004011	0.004226	0.00379
0.00677	0.003969	0.003931	0.004653	0.006478	0.00485	0.003505	0.003982	0.003356
0.012005	0.005618	0.005118	0.005747	0.00675	0.005339	0.004685	0.004457	0.0039
0.011914	0.005524	0.005046	0.005661	0.00665	0.005277	0.004644	0.004405	0.003848
0.011833	0.005427	0.004977	0.005618	0.00658	0.005214	0.004582	0.004359	0.003813
0.01659	0.025828	0.007667	0.01216	0.004783	0.002791	0.013873	0.003469	0.002826
0.016392	0.025617	0.007728	0.011962	0.004751	0.002769	0.013984	0.003288	0.002773
0.016311	0.025239	0.007685	0.011616	0.004579	0.002704	0.013875	0.003122	0.002678

ppm 4.3	ppm 4.34	ppm 4.38	ppm 4.42	ppm 4.46	ppm 4.5	ppm 4.54	ppm 4.58	ppm 4.62
0.00353	0.004349	0.005652	0.00388	0.004379	0.004449	0.035487	0.007343	0.003647
0.003476	0.004391	0.005467	0.003861	0.00439	0.004771	0.034214	0.007237	0.003648
0.003488	0.004345	0.005598	0.003838	0.004339	0.004499	0.035942	0.007371	0.003613
0.003447	0.004716	0.005481	0.0034	0.003689	0.004099	0.016939	0.004936	0.003501
0.003372	0.004678	0.005277	0.003328	0.003638	0.004104	0.016587	0.004731	0.003356
0.003383	0.004634	0.00546	0.003344	0.003634	0.004032	0.016926	0.004935	0.00344
0.00374	0.004597	0.00412	0.003584	0.003601	0.003812	0.006118	0.004417	0.003471
0.003858	0.004758	0.003998	0.003439	0.003421	0.003568	0.005478	0.004212	0.003324
0.003911	0.004724	0.004293	0.003746	0.003727	0.003844	0.005921	0.004652	0.003642
0.003666	0.004289	0.00573	0.003964	0.003943	0.004504	0.03581	0.003947	0.002958
0.003643	0.004223	0.005739	0.003944	0.003882	0.004258	0.036339	0.003813	0.002859
0.00363	0.004225	0.005712	0.003969	0.003938	0.004458	0.035988	0.003552	0.002844
0.00359	0.004883	0.005054	0.003338	0.003454	0.003594	0.005757	0.003743	0.003397
0.003621	0.004855	0.005048	0.003288	0.003392	0.003531	0.005692	0.003696	0.003334
0.003656	0.004871	0.005005	0.003313	0.003411	0.00358	0.005984	0.003783	0.00337
0.003948	0.00377	0.003694	0.003398	0.003289	0.003403	0.004891	0.003856	0.003293
0.003978	0.003792	0.003621	0.003337	0.003233	0.003341	0.004851	0.003794	0.003249
0.004211	0.004044	0.003868	0.003569	0.003401	0.003455	0.004895	0.004131	0.00346
0.003926	0.005401	0.00557	0.003972	0.004396	0.004211	0.0097	0.004895	0.003347
0.003892	0.005167	0.005439	0.003838	0.004175	0.003947	0.009217	0.004854	0.003358
0.003952	0.005237	0.005596	0.003941	0.004309	0.004075	0.009251	0.004949	0.003391
0.002904	0.003605	0.003783	0.002421	0.00244	0.002641	0.010629	0.01425	0.009899
0.002963	0.003629	0.003806	0.002475	0.002477	0.002673	0.010415	0.014378	0.009426
0.002917	0.003592	0.003771	0.002442	0.002455	0.002644	0.010555	0.01435	0.009788
0.003589	0.003729	0.004749	0.003404	0.003111	0.003241	0.01872	0.010822	0.005022
0.003629	0.003787	0.004796	0.003434	0.00316	0.003273	0.01855	0.010102	0.005576
0.003567	0.003717	0.004682	0.003394	0.003124	0.003376	0.018368	0.010391	0.00545
0.003933	0.004754	0.006561	0.004001	0.003988	0.004004	0.005771	0.004588	0.003765
0.003782	0.00475	0.006203	0.003725	0.003837	0.003893	0.005632	0.004334	0.00357
0.003988	0.004782	0.00653	0.004048	0.004058	0.00405	0.005786	0.004622	0.003837
0.004185	0.005376	0.00516	0.003887	0.005708	0.003885	0.005198	0.00395	0.003508

0.004061	0.005097	0.004746	0.003782	0.005114	0.003791	0.004761	0.003876	0.003532
0.003847	0.005159	0.004607	0.003506	0.005281	0.003451	0.004778	0.003452	0.00315
0.004318	0.005761	0.005467	0.003867	0.003914	0.003945	0.005227	0.003916	0.003614
0.0043	0.005748	0.005436	0.003838	0.003881	0.003915	0.005182	0.003874	0.003535
0.004297	0.005691	0.005351	0.003802	0.003863	0.003902	0.005131	0.003832	0.003519
0.003157	0.003558	0.003495	0.002881	0.002661	0.002456	0.004163	0.007283	0.00385
0.003156	0.003528	0.00346	0.002854	0.002611	0.002442	0.004185	0.007488	0.003628
0.00308	0.00346	0.003404	0.002783	0.002582	0.00242	0.004172	0.007378	0.003606

ppm 4.66	ppm 5.02	ppm 5.06	ppm 5.1	ppm 5.14	ppm 5.18	ppm 5.22	ppm 5.26	ppm 5.3
0.003966	0.002785	0.002777	0.002795	0.003135	0.003892	0.003086	0.002951	0.003038
0.003995	0.002719	0.002729	0.00276	0.00309	0.003821	0.003038	0.002901	0.002975
0.003944	0.002744	0.002745	0.00277	0.003101	0.003875	0.00305	0.002905	0.002988
0.004159	0.00257	0.002673	0.002727	0.003161	0.003614	0.002986	0.003511	0.002814
0.004052	0.00253	0.002627	0.002692	0.003132	0.003565	0.002951	0.003465	0.002776
0.004039	0.002532	0.002626	0.002676	0.003102	0.003563	0.002958	0.003475	0.002788
0.00407	0.003118	0.003287	0.003177	0.003313	0.006852	0.003545	0.003484	0.003619
0.003859	0.002893	0.003044	0.002963	0.003101	0.006177	0.003467	0.003502	0.003811
0.004132	0.00322	0.00336	0.003253	0.003316	0.006578	0.003849	0.00362	0.003715
0.004035	0.002559	0.002633	0.002694	0.003185	0.005348	0.003179	0.002945	0.002868
0.003588	0.00255	0.002623	0.002669	0.003047	0.004994	0.00316	0.002929	0.002867
0.003651	0.002525	0.002607	0.002666	0.003037	0.00499	0.003119	0.002903	0.002841
0.004006	0.003054	0.003025	0.002993	0.003339	0.005191	0.003753	0.003398	0.003429
0.003946	0.002971	0.002955	0.002923	0.003277	0.005006	0.003696	0.003404	0.003506
0.004056	0.002978	0.002963	0.002946	0.003309	0.005184	0.00374	0.003423	0.003563
0.003683	0.003144	0.003193	0.003183	0.003381	0.005109	0.003703	0.003938	0.004484
0.003657	0.003094	0.003133	0.003115	0.003313	0.005045	0.003644	0.003925	0.004553
0.003787	0.003158	0.003194	0.003163	0.003276	0.004995	0.00382	0.003927	0.004309
0.003819	0.003066	0.003215	0.003449	0.003904	0.004903	0.0037	0.003272	0.003571
0.003576	0.002993	0.003107	0.003311	0.003681	0.004688	0.003745	0.003401	0.003976
0.003643	0.003032	0.003166	0.003375	0.003792	0.004818	0.003755	0.003381	0.003834
0.002635	0.001951	0.002014	0.002005	0.002243	0.00984	0.007995	0.002213	0.002024
0.002675	0.002017	0.00207	0.002064	0.002281	0.009718	0.007875	0.00231	0.002106
0.002561	0.001974	0.002026	0.002026	0.002249	0.009859	0.00798	0.002234	0.002039
0.00324	0.002449	0.002499	0.002624	0.002855	0.007364	0.00503	0.002914	0.002998
0.003283	0.002455	0.002505	0.002634	0.002861	0.006935	0.005229	0.002943	0.003018
0.003488	0.002443	0.002499	0.002624	0.002856	0.007115	0.00516	0.002931	0.002994
0.004111	0.003319	0.003301	0.003292	0.00365	0.004927	0.003632	0.003607	0.003572
0.003982	0.003228	0.003218	0.00324	0.003634	0.004816	0.003465	0.003489	0.00344
0.004161	0.00343	0.003399	0.003385	0.003695	0.00501	0.003714	0.003691	0.003656
0.005231	0.003131	0.003247	0.00312	0.003339	0.003947	0.003342	0.0033	0.003266

0.004727	0.003246	0.003323	0.003221	0.003366	0.003861	0.003418	0.003451	0.003671
0.005043	0.002826	0.002945	0.00283	0.003116	0.003642	0.00307	0.003099	0.003269
0.004426	0.003194	0.003417	0.003112	0.003356	0.00404	0.003564	0.003349	0.003471
0.00422	0.003182	0.003408	0.0031	0.003335	0.004031	0.003544	0.003337	0.003489
0.004246	0.00318	0.003392	0.003091	0.003329	0.00398	0.003538	0.00332	0.003469
0.002764	0.003195	0.003362	0.002711	0.002569	0.004903	0.009237	0.002749	0.002318
0.002829	0.003308	0.003197	0.002718	0.002512	0.006351	0.007795	0.002728	0.002293
0.002769	0.003232	0.00326	0.002686	0.002558	0.005579	0.008515	0.002682	0.002262

ppm 5.34	ppm 5.38	ppm 5.42	ppm 5.46	ppm 5.5	ppm 5.54	ppm 5.58	ppm 5.62	ppm 5.66
0.002807	0.002924	0.002758	0.002779	0.002723	0.00262	0.002594	0.002594	0.00262
0.002776	0.002895	0.002737	0.002744	0.002695	0.002596	0.002572	0.002576	0.002601
0.002771	0.002892	0.002714	0.002741	0.002684	0.002581	0.002559	0.002557	0.00258
0.002707	0.002776	0.002762	0.002818	0.002814	0.002625	0.002696	0.0026	0.002669
0.002682	0.002765	0.002747	0.002781	0.002782	0.002602	0.002682	0.00258	0.002646
0.002673	0.002739	0.002745	0.002779	0.002786	0.002583	0.00266	0.002557	0.002627
0.004875	0.004673	0.003844	0.003228	0.003147	0.003009	0.00297	0.00295	0.002954
0.007111	0.005684	0.003643	0.003083	0.002957	0.002817	0.002777	0.002769	0.00277
0.005034	0.004833	0.004029	0.003451	0.003336	0.003195	0.00314	0.003121	0.003107
0.002721	0.00271	0.00262	0.002644	0.002572	0.002478	0.002474	0.002434	0.002441
0.002714	0.002711	0.002625	0.002619	0.00255	0.002482	0.002467	0.002431	0.002438
0.002685	0.002687	0.0026	0.002606	0.002535	0.002459	0.002446	0.002421	0.002435
0.004054	0.003357	0.003042	0.003011	0.002953	0.002887	0.002849	0.002847	0.002858
0.004639	0.003655	0.003034	0.002979	0.002907	0.002826	0.002794	0.002788	0.002798
0.004573	0.003712	0.00306	0.002985	0.002921	0.002836	0.002798	0.002789	0.002801
0.005725	0.005392	0.004009	0.003143	0.003074	0.002882	0.002848	0.002853	0.002862
0.006221	0.005618	0.003891	0.003064	0.002988	0.002807	0.002768	0.002781	0.002793
0.006112	0.005859	0.00431	0.003405	0.003308	0.003082	0.002999	0.002994	0.002983
0.003533	0.003387	0.00307	0.002902	0.002824	0.002763	0.002697	0.002702	0.002728
0.00458	0.004031	0.003213	0.002917	0.002822	0.002747	0.002688	0.00268	0.002694
0.004157	0.003754	0.003159	0.002927	0.002846	0.00277	0.002706	0.002696	0.002715
0.002092	0.002113	0.002044	0.002033	0.001942	0.001915	0.001888	0.001879	0.001874
0.00217	0.002189	0.002131	0.002107	0.002017	0.001991	0.001958	0.001943	0.001941
0.00211	0.002135	0.002072	0.00205	0.001957	0.001938	0.001908	0.001895	0.001899
0.002564	0.002683	0.00256	0.002524	0.002442	0.002384	0.002352	0.002337	0.002354
0.00259	0.002702	0.002574	0.002534	0.002452	0.002381	0.002361	0.002348	0.002363
0.00257	0.002683	0.002575	0.00253	0.002453	0.002394	0.002367	0.002352	0.002362
0.003399	0.003588	0.003531	0.00342	0.003315	0.003226	0.003198	0.003209	0.003204
0.003298	0.003517	0.003445	0.003318	0.003236	0.003141	0.003122	0.003147	0.003142
0.003481	0.003679	0.003643	0.003519	0.00342	0.003332	0.003306	0.003304	0.003297
0.003343	0.003793	0.005109	0.003261	0.003863	0.005564	0.003699	0.00325	0.003047

0.004265	0.004272	0.004852	0.003418	0.003751	0.005043	0.003756	0.003389	0.003253
0.003697	0.003851	0.004825	0.002939	0.003681	0.005218	0.003266	0.002884	0.00271
0.003336	0.003514	0.003644	0.003199	0.003305	0.003404	0.003077	0.002988	0.00297
0.003382	0.003552	0.003641	0.003173	0.003298	0.003395	0.003081	0.00297	0.002964
0.00334	0.003546	0.003651	0.00316	0.003282	0.003381	0.003064	0.002982	0.002949
0.002474	0.002454	0.013482	0.002386	0.002121	0.002099	0.002195	0.002044	0.002041
0.002395	0.002432	0.013516	0.002342	0.002126	0.002103	0.002191	0.002028	0.002032
0.00233	0.002461	0.01345	0.002238	0.002075	0.002069	0.00216	0.002009	0.002013

ppm 5.7	ppm 5.74	ppm 5.78	ppm 5.82	ppm 5.86	ppm 5.9	ppm 5.94	ppm 5.98	ppm 6.02
0.002608	0.002592	0.002618	0.002754	0.002612	0.002634	0.002631	0.00275	0.00266
0.00259	0.002586	0.0026	0.002729	0.002606	0.00262	0.002622	0.002743	0.002653
0.002568	0.002566	0.002581	0.002715	0.002575	0.002589	0.002594	0.002715	0.002624
0.00263	0.002601	0.002645	0.002603	0.002564	0.002575	0.002561	0.002603	0.002591
0.00261	0.002583	0.002631	0.002586	0.002556	0.002568	0.002557	0.002603	0.002581
0.002595	0.002562	0.002607	0.002561	0.002537	0.002536	0.00253	0.002569	0.002553
0.002971	0.002986	0.003004	0.003059	0.002981	0.002973	0.00298	0.002994	0.002997
0.002768	0.002752	0.002833	0.002878	0.002787	0.002759	0.002775	0.002791	0.002789
0.003105	0.003124	0.003124	0.003184	0.003113	0.003091	0.003106	0.003115	0.003106
0.002445	0.002448	0.002447	0.002626	0.002476	0.002469	0.002454	0.002476	0.002451
0.002436	0.002456	0.002439	0.002628	0.002474	0.002463	0.002442	0.002468	0.002444
0.002432	0.00244	0.002436	0.002618	0.002464	0.002459	0.002445	0.00247	0.002443
0.002854	0.002844	0.002839	0.00291	0.002858	0.002871	0.002862	0.002911	0.002975
0.002796	0.002783	0.002786	0.002852	0.0028	0.002804	0.002808	0.002857	0.00293
0.002795	0.002779	0.002793	0.002846	0.002808	0.0028	0.002807	0.002856	0.00291
0.002873	0.00286	0.00291	0.002923	0.002887	0.00291	0.002913	0.002918	0.002903
0.002798	0.002794	0.002845	0.002861	0.002836	0.002833	0.002844	0.002854	0.002848
0.002971	0.002952	0.002985	0.003015	0.002968	0.002963	0.002977	0.002979	0.002962
0.002735	0.00275	0.002856	0.002841	0.002733	0.002744	0.002753	0.002773	0.002778
0.002701	0.002696	0.002794	0.00281	0.002699	0.002698	0.002701	0.002704	0.002709
0.002725	0.002729	0.002818	0.002828	0.002714	0.002728	0.002716	0.002724	0.002736
0.001924	0.001873	0.001879	0.001958	0.001899	0.001925	0.001856	0.001854	0.001881
0.001989	0.001944	0.001944	0.002023	0.001968	0.001985	0.001923	0.001917	0.00194
0.001943	0.0019	0.001902	0.001983	0.00192	0.001948	0.001885	0.001873	0.001904
0.002363	0.002341	0.002345	0.002545	0.002341	0.002361	0.002332	0.002336	0.002343
0.002377	0.002334	0.002359	0.002539	0.002348	0.002362	0.002332	0.002345	0.002342
0.002382	0.002349	0.002361	0.002549	0.00236	0.002361	0.002334	0.00234	0.002345
0.003177	0.003176	0.003166	0.003177	0.003169	0.003177	0.003177	0.003179	0.003199
0.003133	0.003121	0.003119	0.003135	0.003125	0.003154	0.003141	0.003159	0.003156
0.003288	0.003282	0.003272	0.003267	0.003253	0.003258	0.00326	0.003255	0.003264
0.003017	0.003094	0.00304	0.003089	0.00304	0.003022	0.003021	0.003072	0.003039

0.00321	0.003254	0.003212	0.003259	0.003222	0.003189	0.003182	0.003218	0.003202
0.002706	0.002799	0.002751	0.002826	0.002775	0.002761	0.002762	0.002816	0.002789
0.002957	0.002947	0.002929	0.003022	0.002983	0.002981	0.002964	0.003027	0.003043
0.002957	0.00294	0.002929	0.003018	0.002988	0.002981	0.002967	0.003018	0.003031
0.002942	0.002935	0.002919	0.003022	0.002982	0.002979	0.002961	0.003017	0.003033
0.002066	0.002021	0.002023	0.002044	0.002043	0.002059	0.002036	0.002033	0.002046
0.002057	0.002014	0.00201	0.002027	0.002028	0.002037	0.002022	0.002019	0.00202
0.002033	0.001991	0.001995	0.00201	0.002014	0.002028	0.002012	0.002011	0.002016

ppm	ppm	ppm	ppm	ppm	ppm	ppm	ppm	ppm
**6.06**	**6.1**	**6.14**	**6.18**	**6.22**	**6.26**	**6.3**	**6.34**	**6.38**
0.002696	0.002735	0.002686	0.002666	0.002675	0.002694	0.002741	0.002788	0.00275
0.002657	0.002743	0.002675	0.002675	0.002675	0.00269	0.002739	0.002779	0.00275
0.002635	0.002713	0.002648	0.002635	0.002639	0.002661	0.002706	0.00276	0.002711
0.002703	0.002682	0.002622	0.002634	0.002614	0.002607	0.002633	0.002765	0.002656
0.002677	0.002687	0.002623	0.002625	0.002609	0.002613	0.002635	0.002771	0.002659
0.002648	0.002658	0.002586	0.002584	0.002573	0.002572	0.002597	0.00273	0.002621
0.00299	0.002995	0.002987	0.002995	0.003014	0.003035	0.003004	0.003069	0.003071
0.002788	0.002786	0.002772	0.002777	0.002792	0.002811	0.002781	0.002829	0.002835
0.003098	0.003104	0.003084	0.00309	0.0031	0.003123	0.003086	0.003134	0.003133
0.002458	0.002724	0.002464	0.002503	0.002542	0.002495	0.002499	0.002627	0.002506
0.002464	0.002732	0.002456	0.002497	0.002542	0.002489	0.002503	0.002631	0.002502
0.002464	0.002721	0.002458	0.002494	0.00254	0.002492	0.002502	0.002636	0.002507
0.002963	0.00287	0.002877	0.002871	0.002874	0.002898	0.002955	0.002924	0.00292
0.00289	0.002811	0.002829	0.002815	0.002811	0.00284	0.002889	0.002848	0.002854
0.002892	0.002812	0.002826	0.002813	0.002805	0.002835	0.002892	0.002856	0.00285
0.002909	0.002897	0.002916	0.002945	0.002968	0.002941	0.002938	0.002974	0.002999
0.002839	0.002844	0.002853	0.002865	0.002895	0.002868	0.002869	0.002918	0.002945
0.002944	0.002948	0.002954	0.002963	0.002995	0.002952	0.002952	0.00298	0.003008
0.002896	0.003194	0.002773	0.002781	0.00281	0.002828	0.002826	0.002922	0.002879
0.002794	0.003092	0.002715	0.002714	0.002738	0.002737	0.002734	0.002819	0.002779
0.002838	0.003159	0.002737	0.002739	0.002762	0.002767	0.002756	0.002858	0.002815
0.001862	0.001916	0.001863	0.001865	0.001879	0.001881	0.001875	0.002009	0.001963
0.001919	0.001972	0.001922	0.001923	0.001946	0.001932	0.001944	0.002065	0.002017
0.001884	0.001939	0.001878	0.00189	0.001905	0.001899	0.001902	0.002035	0.001984
0.00232	0.002386	0.002329	0.002337	0.002339	0.002359	0.002367	0.002452	0.002371
0.002328	0.002388	0.002334	0.002335	0.002344	0.002373	0.002373	0.00246	0.00238
0.002322	0.002393	0.002332	0.002329	0.00234	0.002363	0.002372	0.002447	0.002369
0.003219	0.004196	0.003392	0.003251	0.003226	0.003247	0.003228	0.003276	0.003244
0.003209	0.004182	0.003273	0.003219	0.003185	0.003215	0.0032	0.003258	0.003224
0.003276	0.004193	0.00348	0.003331	0.003298	0.003328	0.0033	0.003353	0.003307
0.003089	0.003022	0.003022	0.003039	0.003031	0.003032	0.00303	0.003044	0.003025

0.00323	0.003179	0.003182	0.003178	0.00319	0.003187	0.003174	0.003182	0.003175
0.002831	0.002774	0.00278	0.002794	0.002797	0.002798	0.002805	0.002813	0.002824
0.003079	0.002965	0.002934	0.003107	0.003057	0.002953	0.002948	0.002969	0.00298
0.003068	0.002963	0.002934	0.00312	0.003065	0.002959	0.002952	0.002978	0.002971
0.00306	0.002953	0.002923	0.003111	0.003059	0.00295	0.002943	0.002962	0.002976
0.002023	0.002107	0.002026	0.002043	0.002037	0.002093	0.002144	0.00209	0.002052
0.002001	0.002084	0.002004	0.002027	0.002016	0.002073	0.002121	0.002062	0.002024
0.001999	0.00209	0.002001	0.002024	0.002022	0.002071	0.002124	0.002071	0.002034

ppm 6.42	ppm 6.46	ppm 6.5	ppm 6.54	ppm 6.58	ppm 6.62	ppm 6.66	ppm 6.7	ppm 6.74
0.002863	0.002786	0.002798	0.002879	0.00297	0.002923	0.003022	0.003246	0.003659
0.002848	0.002791	0.002798	0.002884	0.00297	0.002935	0.003045	0.003276	0.003658
0.002816	0.002749	0.002762	0.002838	0.00293	0.002886	0.002983	0.003216	0.003605
0.002686	0.002671	0.002713	0.002731	0.002817	0.002783	0.002806	0.002883	0.002966
0.002694	0.002683	0.002715	0.002739	0.002813	0.002785	0.002816	0.002893	0.002988
0.002655	0.002632	0.002668	0.002689	0.002771	0.002727	0.002762	0.00283	0.002911
0.003078	0.003143	0.003098	0.003155	0.003145	0.003209	0.003311	0.003375	0.003544
0.002851	0.002899	0.00286	0.002915	0.002914	0.002963	0.003061	0.003126	0.003281
0.003148	0.003196	0.00315	0.003207	0.003194	0.003231	0.003325	0.003378	0.003537
0.002495	0.002509	0.002618	0.002633	0.002698	0.002824	0.003167	0.002872	0.003651
0.002481	0.002499	0.002618	0.002622	0.002684	0.002815	0.00319	0.002844	0.003673
0.0025	0.002512	0.002635	0.002642	0.002712	0.002833	0.003228	0.002903	0.003735
0.002913	0.002924	0.002925	0.00303	0.002995	0.002972	0.003	0.003053	0.00309
0.002842	0.002858	0.002848	0.002958	0.002927	0.002902	0.00293	0.002983	0.003013
0.002847	0.002861	0.002861	0.002956	0.002921	0.002908	0.002933	0.00299	0.003017
0.003005	0.003056	0.00302	0.003425	0.003058	0.003106	0.003164	0.003269	0.003393
0.002936	0.003003	0.002956	0.003375	0.002988	0.003044	0.003107	0.003218	0.003341
0.002996	0.00307	0.002993	0.0034	0.003085	0.003085	0.003138	0.003231	0.00336
0.002913	0.002929	0.002945	0.003081	0.003042	0.003099	0.003166	0.003339	0.003582
0.002803	0.002815	0.002822	0.002939	0.002912	0.002928	0.002984	0.003118	0.003336
0.002843	0.002857	0.002867	0.002991	0.002947	0.002991	0.003055	0.003204	0.003421
0.001879	0.001887	0.001896	0.001922	0.001921	0.001984	0.001982	0.002101	0.002215
0.001941	0.001936	0.001946	0.001974	0.001968	0.002035	0.002026	0.002137	0.002237
0.001904	0.001909	0.001916	0.001949	0.001942	0.002011	0.002001	0.002113	0.00223
0.002413	0.002367	0.002362	0.002488	0.002463	0.002471	0.002465	0.002581	0.002643
0.002419	0.00238	0.002377	0.002515	0.002485	0.002479	0.002474	0.002598	0.002664
0.002407	0.00236	0.002356	0.002482	0.002446	0.002457	0.002441	0.002552	0.002615
0.003246	0.003658	0.003748	0.003284	0.003334	0.003313	0.003344	0.003432	0.003517
0.003247	0.003659	0.003676	0.003249	0.003319	0.003308	0.003338	0.003435	0.003546
0.003306	0.003688	0.003791	0.003356	0.003411	0.003376	0.003395	0.003478	0.003564
0.003042	0.003039	0.003024	0.003105	0.003047	0.003046	0.003072	0.00309	0.003158

0.003178	0.003175	0.003175	0.003225	0.003185	0.003186	0.003202	0.003212	0.003254
0.002817	0.002823	0.002823	0.002908	0.002834	0.002851	0.002876	0.002909	0.002976
0.002963	0.002926	0.002928	0.003033	0.00294	0.002944	0.002972	0.002988	0.003073
0.002963	0.002935	0.002935	0.003021	0.002937	0.002952	0.002974	0.002986	0.003067
0.002952	0.002926	0.002929	0.003031	0.002942	0.002945	0.002969	0.002971	0.003068
0.002031	0.002032	0.002044	0.002248	0.002116	0.002105	0.0021	0.002205	0.002302
0.002002	0.001999	0.002006	0.002217	0.002074	0.002071	0.002063	0.002166	0.002268
0.002008	0.002016	0.002016	0.002233	0.002096	0.002086	0.002081	0.002181	0.002287

ppm 6.78	ppm 6.82	ppm 6.86	ppm 6.9	ppm 6.94	ppm 6.98	ppm 7.02	ppm 7.06	ppm 7.1
0.003707	0.004118	0.036173	0.003558	0.003646	0.003707	0.012724	0.005235	0.003494
0.003764	0.004357	0.034955	0.003899	0.003662	0.003784	0.01246	0.00521	0.003505
0.003647	0.004087	0.036552	0.003702	0.003647	0.003719	0.0129	0.005257	0.003504
0.003126	0.003263	0.015412	0.003767	0.003634	0.003627	0.004543	0.00431	0.003771
0.003153	0.003277	0.015185	0.003755	0.003631	0.003627	0.004512	0.004296	0.003776
0.003058	0.003137	0.015502	0.003892	0.003641	0.003625	0.004548	0.004324	0.003767
0.003623	0.00371	0.004015	0.003874	0.003764	0.003616	0.003511	0.003645	0.003525
0.003355	0.003476	0.0037	0.003555	0.003445	0.00329	0.003235	0.003346	0.003222
0.00362	0.003731	0.004002	0.003914	0.003817	0.003679	0.003577	0.003708	0.003579
0.003304	0.003481	0.035499	0.005166	0.003476	0.003436	0.015878	0.005008	0.003306
0.003274	0.00336	0.037104	0.005348	0.003499	0.003412	0.016532	0.005149	0.003325
0.003371	0.003643	0.036904	0.005071	0.003466	0.003482	0.016403	0.005007	0.00328
0.003135	0.00318	0.003383	0.00322	0.003223	0.003257	0.00324	0.003269	0.003275
0.003069	0.003121	0.003289	0.003132	0.003139	0.003179	0.003154	0.003192	0.003204
0.003072	0.003114	0.0033	0.00314	0.003141	0.003172	0.003156	0.003186	0.003195
0.003478	0.00355	0.003737	0.003642	0.003525	0.003341	0.003337	0.003322	0.003341
0.003421	0.003492	0.003684	0.00359	0.00346	0.003276	0.003276	0.003262	0.003288
0.003441	0.003551	0.003735	0.003715	0.003614	0.003431	0.003401	0.003414	0.003405
0.003827	0.003849	0.008481	0.003358	0.00335	0.003585	0.005096	0.003583	0.003427
0.00357	0.003509	0.008195	0.003468	0.00331	0.003469	0.005018	0.003587	0.003422
0.003662	0.00363	0.008165	0.003463	0.003339	0.003515	0.005	0.003595	0.003431
0.002223	0.002313	0.009613	0.004113	0.002446	0.00222	0.003932	0.010923	0.00227
0.002241	0.002312	0.009423	0.004149	0.002506	0.002251	0.003887	0.010738	0.002409
0.002239	0.002334	0.009611	0.00412	0.00245	0.00223	0.003907	0.010761	0.002292
0.002738	0.002732	0.017785	0.00462	0.003048	0.002849	0.008301	0.006036	0.00291
0.002783	0.002817	0.017675	0.004713	0.00306	0.002891	0.008324	0.00594	0.002927
0.002707	0.002744	0.017417	0.00466	0.003088	0.002896	0.008197	0.006003	0.002953
0.003635	0.003718	0.004635	0.004343	0.003787	0.003619	0.003652	0.00452	0.003561
0.003652	0.003725	0.004593	0.00423	0.00371	0.003558	0.003623	0.004384	0.003486
0.003678	0.003737	0.004591	0.004361	0.003839	0.003673	0.003675	0.004524	0.003627
0.003209	0.003242	0.003314	0.0033	0.003287	0.00322	0.0032	0.003245	0.003259

0.003305	0.003344	0.003376	0.003364	0.003354	0.003303	0.003282	0.003336	0.003323
0.003031	0.00306	0.003133	0.003097	0.003072	0.003012	0.003	0.00304	0.003063
0.003129	0.003163	0.003202	0.003185	0.00314	0.00311	0.003066	0.003096	0.00316
0.003125	0.003161	0.003194	0.003176	0.003125	0.003114	0.003067	0.003093	0.003153
0.003123	0.003155	0.0032	0.003173	0.003132	0.003113	0.003065	0.0031	0.003154
0.002337	0.002646	0.003863	0.002781	0.00279	0.002729	0.003345	0.003942	0.00398
0.002287	0.002607	0.00383	0.002746	0.002744	0.002671	0.003299	0.003882	0.004134
0.002305	0.002627	0.003836	0.00275	0.002769	0.002695	0.003338	0.003897	0.004095

ppm 7.14	ppm 7.18	ppm 7.22	ppm 7.26	ppm 7.3	ppm 7.34	ppm 7.38	ppm 7.42	ppm 7.46
0.003711	0.003386	0.003529	0.015491	0.003095	0.003438	0.002981	0.002829	0.002853
0.003686	0.003397	0.003615	0.014949	0.003227	0.003429	0.003004	0.002842	0.002876
0.003699	0.003377	0.003509	0.015589	0.003146	0.00345	0.002999	0.002825	0.002857
0.00349	0.003359	0.003163	0.012503	0.003498	0.003387	0.00317	0.003193	0.002998
0.003487	0.003361	0.003156	0.012339	0.003493	0.003403	0.003192	0.003216	0.003017
0.003489	0.00333	0.003068	0.012575	0.003623	0.003428	0.003217	0.003217	0.003013
0.003513	0.003464	0.003393	0.003429	0.003506	0.003414	0.003399	0.003315	0.003292
0.003222	0.003179	0.003104	0.003141	0.003205	0.003126	0.003104	0.003017	0.002974
0.00358	0.003544	0.003467	0.003499	0.003585	0.003498	0.003482	0.003418	0.003364
0.003282	0.003048	0.003015	0.010286	0.003179	0.00327	0.002999	0.00313	0.003065
0.003307	0.003066	0.002995	0.010679	0.003248	0.003242	0.003011	0.003157	0.003079
0.003265	0.003045	0.003028	0.010567	0.003168	0.003198	0.002969	0.00313	0.003048
0.003383	0.003387	0.003485	0.003508	0.003364	0.003249	0.003206	0.00332	0.003153
0.003305	0.003319	0.003396	0.003436	0.003275	0.003178	0.003134	0.003238	0.003081
0.00329	0.00331	0.003382	0.00341	0.003267	0.00316	0.003121	0.00322	0.003069
0.003291	0.003256	0.003221	0.003231	0.003294	0.003229	0.003238	0.003146	0.003062
0.003226	0.003194	0.003147	0.003162	0.003231	0.003159	0.00317	0.003083	0.003001
0.003371	0.00334	0.003291	0.003297	0.003376	0.003323	0.00332	0.003247	0.00317
0.003413	0.003749	0.003218	0.003974	0.003057	0.003241	0.003068	0.002911	0.002893
0.003354	0.00374	0.003214	0.003985	0.003096	0.003253	0.003131	0.002958	0.002928
0.003399	0.003752	0.003228	0.00396	0.003101	0.003256	0.003106	0.002944	0.002924
0.002198	0.002174	0.002183	0.00864	0.002222	0.002197	0.002194	0.0021	0.002081
0.002265	0.002224	0.00221	0.008506	0.002335	0.002266	0.002244	0.002144	0.002089
0.002207	0.002189	0.002191	0.008628	0.002242	0.002218	0.002204	0.002123	0.002097
0.00281	0.002778	0.00269	0.007042	0.002807	0.00274	0.002706	0.002638	0.002575
0.002814	0.002797	0.002717	0.006834	0.002821	0.002749	0.002696	0.00264	0.002596
0.002851	0.002809	0.002729	0.00696	0.002854	0.002786	0.002737	0.00267	0.002611
0.003598	0.003594	0.003532	0.003607	0.003559	0.003552	0.003458	0.003427	0.003385
0.003535	0.003525	0.00349	0.003549	0.003505	0.003494	0.003388	0.003383	0.00334
0.003665	0.00367	0.00361	0.003679	0.00364	0.003636	0.003543	0.003511	0.003479
0.003295	0.003239	0.003211	0.003261	0.003256	0.003244	0.003211	0.003177	0.003151

0.003359	0.003324	0.003302	0.003343	0.003337	0.003326	0.003308	0.003282	0.003272
0.003092	0.003028	0.003009	0.003052	0.003049	0.003043	0.002987	0.002958	0.002943
0.003282	0.003152	0.0031	0.003125	0.003135	0.003209	0.003188	0.003078	0.003054
0.003281	0.003158	0.003101	0.003135	0.003137	0.003208	0.00319	0.003078	0.003048
0.003277	0.003141	0.003106	0.003133	0.00313	0.003214	0.003191	0.003084	0.003055
0.007339	0.003275	0.002858	0.003539	0.003238	0.007901	0.00751	0.003262	0.003014
0.007146	0.003259	0.002843	0.003513	0.003198	0.007972	0.007444	0.003325	0.002979
0.007183	0.003236	0.002849	0.003533	0.003233	0.00804	0.007322	0.003272	0.002988

ppm 7.5	ppm 7.54	ppm 7.58	ppm 7.62	ppm 7.66	ppm 7.7	ppm 7.74	ppm 7.78	ppm 7.82
0.002939	0.00291	0.002796	0.002679	0.002662	0.002603	0.002591	0.002582	0.002584
0.002918	0.002901	0.002816	0.00269	0.002675	0.002613	0.002607	0.002591	0.002592
0.002927	0.002901	0.002801	0.002669	0.002652	0.002594	0.002584	0.002562	0.002566
0.002999	0.002972	0.002909	0.002909	0.002812	0.002753	0.002721	0.00269	0.002674
0.00302	0.002989	0.002932	0.002933	0.002834	0.002769	0.002742	0.002699	0.002693
0.003009	0.002984	0.002937	0.002926	0.002827	0.002755	0.002723	0.002693	0.002676
0.003272	0.003298	0.003182	0.003137	0.003132	0.003114	0.003099	0.003106	0.003085
0.002997	0.002995	0.002903	0.002863	0.002855	0.002834	0.002836	0.002834	0.00282
0.003365	0.003384	0.003281	0.003241	0.003223	0.003206	0.003188	0.003189	0.003178
0.004477	0.004026	0.003422	0.002756	0.002756	0.002599	0.002571	0.002541	0.002538
0.00457	0.004087	0.003486	0.002755	0.002764	0.002604	0.002577	0.002537	0.002531
0.004548	0.00404	0.003421	0.002726	0.002734	0.002581	0.002554	0.002514	0.002532
0.003117	0.003122	0.003244	0.003088	0.003052	0.003036	0.003026	0.00296	0.002942
0.003049	0.003048	0.003173	0.003024	0.002986	0.002968	0.00296	0.002889	0.002881
0.003029	0.003036	0.003151	0.003011	0.002971	0.002957	0.002944	0.002884	0.002869
0.003099	0.00308	0.003041	0.003	0.002979	0.00296	0.002968	0.002971	0.002942
0.003028	0.003013	0.002971	0.002932	0.00291	0.002893	0.002898	0.002887	0.002873
0.003178	0.003171	0.003128	0.003097	0.003066	0.003048	0.003041	0.003034	0.00301
0.002909	0.003038	0.003287	0.003115	0.002863	0.002656	0.002646	0.002629	0.002635
0.002916	0.003022	0.003319	0.003143	0.002975	0.002725	0.002703	0.002688	0.002676
0.002917	0.003027	0.00329	0.003136	0.00293	0.002706	0.002686	0.002669	0.002664
0.011119	0.011227	0.002757	0.002075	0.002058	0.001965	0.001929	0.001898	0.001885
0.010775	0.011161	0.002976	0.002202	0.002162	0.002063	0.002029	0.001982	0.001967
0.010933	0.010997	0.002788	0.002084	0.00207	0.001975	0.001947	0.001908	0.001906
0.005416	0.005527	0.003318	0.00256	0.002544	0.002452	0.002448	0.002442	0.002391
0.005286	0.005383	0.00332	0.002542	0.002531	0.002444	0.002436	0.002436	0.002375
0.005348	0.005521	0.003379	0.002606	0.002596	0.002496	0.002496	0.002486	0.00243
0.003509	0.003416	0.003431	0.003306	0.003281	0.003241	0.003232	0.003216	0.003212
0.003456	0.003362	0.003369	0.003266	0.003231	0.003202	0.003184	0.003178	0.003177
0.003578	0.003498	0.003498	0.003414	0.003381	0.003343	0.003322	0.003311	0.003299
0.003148	0.003168	0.003176	0.00313	0.003116	0.003116	0.003084	0.003077	0.003067

0.003257	0.003272	0.003269	0.003249	0.003231	0.003224	0.003225	0.003199	0.003203
0.00294	0.002973	0.002973	0.002914	0.002915	0.002912	0.002882	0.00288	0.002878
0.003037	0.003051	0.003071	0.003033	0.002993	0.002991	0.002983	0.00297	0.00297
0.003037	0.003052	0.003073	0.003034	0.003007	0.002988	0.002996	0.002986	0.002975
0.003029	0.003056	0.003076	0.003036	0.002995	0.002984	0.002997	0.002981	0.002976
0.003466	0.003233	0.009673	0.00612	0.006227	0.0023	0.002206	0.002151	0.002089
0.003442	0.003202	0.009715	0.006203	0.006273	0.00239	0.002274	0.002211	0.00213
0.003452	0.003225	0.009707	0.006155	0.006126	0.002325	0.002231	0.002172	0.002109

ppm 7.86	ppm 7.9	ppm 7.94	ppm 7.98	ppm 8.02	ppm 8.06	ppm 8.1	ppm 8.14	ppm 8.18
0.002611	0.002622	0.002611	0.002601	0.002588	0.002573	0.002582	0.002566	0.002568
0.002626	0.002631	0.002633	0.002617	0.002597	0.002586	0.002589	0.002583	0.002584
0.002604	0.002602	0.002605	0.002596	0.00256	0.00256	0.002564	0.002555	0.002553
0.00271	0.002665	0.002678	0.002673	0.002678	0.002643	0.002637	0.00263	0.002622
0.00274	0.002701	0.002709	0.002701	0.002701	0.002674	0.00266	0.00266	0.002644
0.002718	0.002676	0.00269	0.002678	0.002677	0.002655	0.002643	0.002633	0.002624
0.003077	0.003078	0.003083	0.003071	0.003078	0.003058	0.003049	0.003036	0.003048
0.002813	0.002807	0.002816	0.002792	0.002807	0.002787	0.002774	0.002773	0.002773
0.003166	0.003163	0.003168	0.003146	0.003165	0.003134	0.003134	0.003119	0.003131
0.003763	0.002764	0.002662	0.002757	0.00271	0.002682	0.002581	0.002541	0.002516
0.003835	0.002795	0.00267	0.002763	0.002716	0.002689	0.002591	0.002536	0.002526
0.003775	0.002784	0.002643	0.002741	0.002697	0.002659	0.002571	0.002516	0.002507
0.00298	0.002951	0.002916	0.002901	0.002915	0.002877	0.002874	0.002886	0.002878
0.002918	0.002885	0.002864	0.00283	0.002844	0.002811	0.002813	0.00281	0.002815
0.002907	0.002875	0.002852	0.002821	0.002841	0.002798	0.002807	0.002809	0.002798
0.002927	0.002953	0.002953	0.002925	0.002935	0.002916	0.002917	0.002921	0.002928
0.002859	0.002891	0.00289	0.002864	0.002868	0.002857	0.002855	0.002863	0.00285
0.002996	0.003009	0.003022	0.002986	0.002996	0.002973	0.002977	0.002976	0.002978
0.002665	0.002675	0.002658	0.00264	0.002646	0.002621	0.002623	0.002616	0.002619
0.002696	0.00271	0.002692	0.002681	0.002679	0.002655	0.002665	0.002656	0.002658
0.002681	0.002699	0.002679	0.002667	0.002671	0.002653	0.002641	0.002645	0.002641
0.003636	0.002288	0.002019	0.001918	0.001915	0.001883	0.001876	0.001876	0.001868
0.003659	0.002399	0.002103	0.002001	0.001993	0.001968	0.00196	0.001956	0.001954
0.003688	0.002251	0.002033	0.001938	0.001925	0.001895	0.001894	0.00189	0.001886
0.003197	0.0025	0.002452	0.002384	0.002375	0.002378	0.002364	0.002353	0.002346
0.003163	0.002472	0.002432	0.002368	0.002358	0.002355	0.002351	0.00234	0.002338
0.003203	0.002549	0.002489	0.002425	0.002408	0.002419	0.0024	0.002387	0.002384
0.003209	0.003223	0.003241	0.003202	0.003196	0.0032	0.003195	0.003203	0.00319
0.003193	0.003193	0.003204	0.00318	0.003177	0.003169	0.003172	0.003164	0.003173
0.003299	0.003309	0.003328	0.003294	0.003296	0.003289	0.003287	0.00329	0.003276
0.003075	0.003076	0.00306	0.003069	0.003077	0.003059	0.003048	0.003043	0.00304

0.003223	0.003189	0.003208	0.003182	0.003195	0.00318	0.00317	0.003164	0.003168
0.002881	0.002899	0.00287	0.002879	0.002891	0.002864	0.002857	0.00286	0.002859
0.00297	0.002998	0.00296	0.002959	0.002971	0.002946	0.002933	0.002936	0.002942
0.002977	0.002981	0.002975	0.002962	0.002973	0.002953	0.002941	0.002945	0.002938
0.00297	0.002987	0.002976	0.00296	0.002964	0.002944	0.002938	0.002931	0.00294
0.002888	0.002239	0.002433	0.002556	0.002111	0.002078	0.00207	0.00205	0.00204
0.002956	0.002252	0.0025	0.002582	0.002152	0.002117	0.002097	0.002083	0.002074
0.002911	0.002238	0.002468	0.002556	0.002123	0.002094	0.00208	0.002056	0.00206

**ppm**	**ppm**	**ppm**	**ppm**	**ppm**	**ppm**	**ppm**	**ppm**	**ppm**
**8.22**	**8.26**	**8.3**	**8.34**	**8.38**	**8.42**	**8.46**	**8.5**	**8.54**
0.002567	0.002568	0.002595	0.002555	0.002558	0.002556	0.00258	0.002742	0.002582
0.002584	0.002585	0.002616	0.002582	0.002571	0.002575	0.002593	0.002764	0.002603
0.002558	0.002552	0.002581	0.002549	0.002538	0.00254	0.002567	0.002734	0.002565
0.00263	0.002613	0.002612	0.002603	0.002604	0.002597	0.002606	0.002636	0.002597
0.002653	0.002631	0.002638	0.002624	0.002618	0.002621	0.002627	0.002653	0.002625
0.002638	0.002625	0.002611	0.002603	0.002599	0.002591	0.002597	0.002633	0.002599
0.003043	0.00305	0.003044	0.003039	0.003033	0.003032	0.003029	0.003111	0.00304
0.002764	0.002778	0.002773	0.002768	0.002766	0.002766	0.002769	0.002836	0.002772
0.003113	0.003113	0.003109	0.003106	0.003096	0.003096	0.003083	0.003179	0.003106
0.00252	0.002523	0.00252	0.002512	0.002517	0.002521	0.002577	0.002645	0.002566
0.002524	0.002518	0.002522	0.002515	0.002515	0.002525	0.002579	0.002637	0.002564
0.002505	0.002508	0.0025	0.002503	0.002494	0.002507	0.002573	0.002619	0.002544
0.002879	0.002867	0.002874	0.002863	0.002859	0.002846	0.002855	0.002878	0.002855
0.002812	0.002796	0.002799	0.002794	0.002788	0.002793	0.002791	0.002812	0.002791
0.002802	0.002794	0.002789	0.002783	0.002783	0.002783	0.002777	0.002812	0.002775
0.002919	0.002912	0.00291	0.002909	0.002909	0.002905	0.002906	0.002975	0.002918
0.002847	0.00285	0.00285	0.002851	0.002846	0.00284	0.002838	0.002903	0.002841
0.002964	0.002972	0.002963	0.002957	0.002956	0.002948	0.002946	0.003016	0.002963
0.002624	0.00263	0.002646	0.00261	0.002607	0.00262	0.002619	0.00266	0.00262
0.002657	0.002653	0.002687	0.00265	0.002644	0.002641	0.002649	0.002686	0.002661
0.002649	0.00264	0.002667	0.002638	0.002625	0.002631	0.002632	0.002673	0.002645
0.00193	0.001867	0.001861	0.001862	0.001863	0.001856	0.001941	0.00186	0.001863
0.002005	0.001949	0.001942	0.001942	0.001936	0.001935	0.002006	0.00194	0.001939
0.001952	0.001889	0.00188	0.001881	0.001885	0.001874	0.001952	0.001878	0.001886
0.002371	0.002349	0.002344	0.002332	0.002329	0.002324	0.002379	0.00234	0.002345
0.002353	0.002329	0.002332	0.002321	0.002311	0.002309	0.002365	0.002324	0.00233
0.002406	0.002381	0.002379	0.002371	0.002367	0.002359	0.002413	0.002371	0.002378
0.003199	0.003192	0.003176	0.003183	0.003189	0.00318	0.003196	0.00318	0.003195
0.003171	0.003167	0.003174	0.003164	0.003176	0.003164	0.003187	0.003167	0.003174
0.00328	0.003278	0.003275	0.003269	0.003275	0.003266	0.003289	0.00328	0.003277
0.003033	0.003036	0.003034	0.00303	0.003035	0.003033	0.00302	0.003068	0.003033

0.003161	0.003174	0.003163	0.003158	0.003161	0.003158	0.003158	0.003181	0.003158
0.002866	0.002865	0.002854	0.002856	0.002856	0.002854	0.002853	0.00289	0.002859
0.002936	0.002937	0.002929	0.00293	0.002924	0.002915	0.002916	0.002961	0.002931
0.002939	0.002938	0.002934	0.002928	0.002937	0.002923	0.002919	0.002976	0.002935
0.002935	0.002946	0.002933	0.002928	0.002933	0.002926	0.002921	0.002964	0.002933
0.00205	0.00206	0.002049	0.002039	0.002025	0.00202	0.002094	0.002048	0.002025
0.002069	0.002085	0.00207	0.002067	0.002053	0.002042	0.002117	0.002069	0.002049
0.002058	0.002073	0.002057	0.002052	0.00204	0.002029	0.00211	0.002056	0.002038

ppm 8.58	ppm 8.62	ppm 8.66	ppm 8.7	ppm 8.74	ppm 8.78	ppm 8.82	ppm 8.86	ppm 8.9
0.002567	0.002579	0.002548	0.002547	0.00256	0.00255	0.002548	0.002553	0.002569
0.002579	0.002604	0.002569	0.002565	0.002571	0.002565	0.00257	0.002577	0.002575
0.002543	0.002567	0.00254	0.002533	0.002536	0.002533	0.002532	0.002537	0.002544
0.002593	0.002585	0.002588	0.002582	0.002584	0.002584	0.002574	0.002583	0.002584
0.002616	0.002613	0.002603	0.002606	0.002605	0.002609	0.002608	0.002618	0.002621
0.002603	0.00259	0.002586	0.002584	0.002594	0.002585	0.002584	0.002582	0.002587
0.003042	0.003039	0.003032	0.003035	0.003032	0.003029	0.003049	0.003034	0.003033
0.00277	0.002774	0.002767	0.002761	0.00277	0.002765	0.002773	0.002766	0.002763
0.003098	0.003106	0.003092	0.003097	0.003088	0.003088	0.003089	0.003087	0.003086
0.002524	0.002507	0.002501	0.002501	0.002497	0.0025	0.002493	0.00251	0.002503
0.002524	0.002504	0.002498	0.002496	0.002496	0.00249	0.002492	0.002503	0.002504
0.002508	0.002492	0.002481	0.002476	0.002482	0.002472	0.002474	0.002497	0.002494
0.002858	0.002852	0.002842	0.002841	0.002844	0.002838	0.002831	0.002845	0.002842
0.002783	0.002786	0.002775	0.002783	0.002781	0.002779	0.002772	0.002776	0.002773
0.002783	0.002778	0.002771	0.002778	0.002764	0.00276	0.00277	0.002764	0.002772
0.002901	0.002914	0.002915	0.002912	0.002912	0.002912	0.00291	0.002908	0.002907
0.002852	0.002844	0.002843	0.002836	0.00284	0.002839	0.002845	0.002842	0.002846
0.002954	0.002952	0.002964	0.002944	0.002955	0.00295	0.002948	0.002938	0.002948
0.002622	0.002629	0.002598	0.002598	0.002599	0.002604	0.002606	0.002599	0.002607
0.002651	0.002668	0.002638	0.002639	0.002638	0.002644	0.002636	0.002646	0.00264
0.002645	0.002655	0.002625	0.002627	0.002627	0.00262	0.002624	0.002629	0.002633
0.001862	0.001853	0.00185	0.001849	0.001846	0.001839	0.001842	0.001852	0.00186
0.001934	0.001932	0.001924	0.001922	0.001925	0.001916	0.001914	0.001922	0.001928
0.001874	0.001878	0.001867	0.001865	0.001867	0.001861	0.001865	0.001868	0.001882
0.002325	0.002333	0.002319	0.002315	0.002323	0.002316	0.002316	0.002312	0.002333
0.002321	0.002311	0.002304	0.002293	0.002301	0.002303	0.002295	0.0023	0.002311
0.002362	0.002363	0.002351	0.002352	0.002353	0.002358	0.002344	0.002354	0.002355
0.00318	0.003182	0.003171	0.003179	0.003174	0.003176	0.003167	0.003181	0.003179
0.003163	0.003159	0.003167	0.003167	0.003157	0.003163	0.003164	0.003162	0.003179
0.003273	0.003266	0.003263	0.003267	0.003261	0.003274	0.003265	0.003264	0.003262
0.00303	0.003031	0.003025	0.003028	0.003031	0.00303	0.003029	0.003025	0.003027

0.003146	0.003157	0.003146	0.003149	0.003146	0.003144	0.003145	0.003148	0.003143
0.002863	0.00286	0.002857	0.00286	0.002866	0.002869	0.002867	0.002869	0.002859
0.002931	0.002923	0.002916	0.002919	0.002922	0.002914	0.002914	0.002922	0.002915
0.002934	0.002935	0.002923	0.002926	0.00293	0.002921	0.002924	0.002929	0.002929
0.002936	0.002931	0.002925	0.002926	0.002927	0.002928	0.002921	0.002918	0.002918
0.002026	0.002031	0.002006	0.002005	0.00201	0.002002	0.002003	0.001998	0.002008
0.002042	0.002063	0.002029	0.002024	0.002026	0.00203	0.002025	0.002023	0.002024
0.002034	0.002051	0.002023	0.002021	0.002022	0.002018	0.002017	0.002016	0.00202

ppm 8.94	ppm 8.98	ppm 9.02	ppm 9.06	ppm 9.1	ppm 9.14	ppm 9.18	ppm 9.22	ppm 9.26
0.002551	0.002584	0.002555	0.002546	0.002553	0.002549	0.002549	0.002545	0.002558
0.002578	0.002605	0.002575	0.00258	0.002576	0.002575	0.002576	0.002575	0.002591
0.002545	0.002565	0.002537	0.002533	0.002533	0.002529	0.002532	0.002531	0.002544
0.002583	0.002587	0.002576	0.002569	0.002579	0.002579	0.002579	0.002579	0.002577
0.002609	0.002607	0.002613	0.002605	0.002606	0.002606	0.002604	0.002597	0.002606
0.002589	0.002574	0.002576	0.002576	0.002583	0.002577	0.002573	0.002576	0.002577
0.003035	0.003033	0.003032	0.00304	0.003023	0.003024	0.003033	0.003031	0.003044
0.002767	0.002772	0.002762	0.002768	0.002763	0.002761	0.00276	0.00276	0.002775
0.003088	0.003084	0.003082	0.003078	0.003072	0.003075	0.003072	0.00308	0.003069
0.002502	0.00249	0.002495	0.002492	0.002482	0.002482	0.002486	0.002486	0.002489
0.002492	0.002492	0.002485	0.002487	0.002485	0.002479	0.002483	0.00248	0.002487
0.002476	0.002479	0.002473	0.002476	0.002468	0.002474	0.002462	0.002469	0.002485
0.00284	0.00284	0.002845	0.002841	0.00284	0.002842	0.002841	0.002852	0.002852
0.002786	0.002778	0.002771	0.002776	0.002775	0.002775	0.002778	0.002789	0.002778
0.002762	0.002766	0.002764	0.002766	0.002771	0.002766	0.00277	0.002773	0.002772
0.002921	0.002914	0.002914	0.002914	0.002911	0.002903	0.002912	0.00292	0.002921
0.002847	0.002851	0.002845	0.002847	0.002845	0.002846	0.002843	0.002838	0.002858
0.002947	0.002945	0.00295	0.002945	0.002954	0.002946	0.002942	0.002944	0.002956
0.002599	0.002635	0.002594	0.002591	0.002593	0.002583	0.002592	0.002587	0.002596
0.002636	0.002672	0.002638	0.002643	0.002632	0.002636	0.00264	0.002635	0.002635
0.002625	0.00266	0.00262	0.00261	0.002622	0.002616	0.002609	0.002613	0.002615
0.00185	0.001851	0.00184	0.001839	0.001834	0.001826	0.001833	0.001831	0.00184
0.001928	0.001921	0.001913	0.001911	0.001904	0.001901	0.001905	0.001898	0.001917
0.001866	0.001874	0.001858	0.001851	0.001853	0.001856	0.001851	0.001845	0.001863
0.002318	0.002312	0.00231	0.002307	0.002305	0.002298	0.002301	0.002297	0.002312
0.002304	0.0023	0.002289	0.002283	0.002289	0.002282	0.002292	0.002286	0.002292
0.002344	0.002346	0.002345	0.002338	0.002334	0.002335	0.002328	0.002331	0.002337
0.003185	0.00318	0.003185	0.003175	0.003175	0.003185	0.003184	0.003179	0.003187
0.003166	0.003171	0.003166	0.003163	0.003154	0.003163	0.003168	0.003171	0.003175
0.003272	0.003259	0.003261	0.003261	0.003259	0.003259	0.003262	0.003269	0.003273
0.003027	0.003029	0.003031	0.003031	0.00303	0.003025	0.003024	0.003032	0.003025

0.00315	0.003156	0.003143	0.003147	0.003151	0.003138	0.003143	0.00314	0.003144
0.002871	0.002869	0.00287	0.002871	0.002869	0.002861	0.002869	0.002866	0.002879
0.002918	0.002915	0.002918	0.00292	0.002921	0.002912	0.002914	0.002918	0.00292
0.002934	0.002926	0.002926	0.002919	0.00293	0.002925	0.002918	0.002926	0.002924
0.002926	0.002916	0.002918	0.002923	0.002914	0.002909	0.002915	0.002925	0.00292
0.002003	0.002019	0.002004	0.001999	0.001993	0.001994	0.001988	0.001988	0.001991
0.002017	0.00203	0.002012	0.002017	0.002011	0.002003	0.002001	0.001996	0.002005
0.002013	0.002027	0.002017	0.002007	0.002005	0.002006	0.001999	0.002005	0.002003

ppm<br>9.3	ppm<br>9.34	ppm<br>9.38	ppm<br>9.42	ppm<br>9.46	ppm<br>9.5	ppm<br>9.54	ppm<br>9.58	ppm<br>9.62
0.002559	0.00256	0.002556	0.002555	0.002553	0.002575	0.002567	0.002563	0.002567
0.002576	0.002581	0.002578	0.002585	0.002585	0.002592	0.002593	0.002594	0.002607
0.002536	0.002534	0.002546	0.002542	0.002538	0.002559	0.002556	0.002548	0.002556
0.00258	0.002588	0.002579	0.002581	0.002586	0.0026	0.002647	0.002621	0.002618
0.002607	0.002609	0.002613	0.002616	0.002624	0.00263	0.002667	0.002652	0.002642
0.002579	0.00258	0.002586	0.002583	0.002594	0.002601	0.002653	0.002623	0.002613
0.003039	0.003042	0.003043	0.003042	0.00304	0.003048	0.00304	0.003059	0.003046
0.00277	0.002774	0.00277	0.002785	0.002772	0.002772	0.002776	0.002792	0.002781
0.003077	0.003081	0.003076	0.00307	0.003074	0.003068	0.003077	0.003087	0.003081
0.002486	0.00248	0.002487	0.002485	0.002479	0.002482	0.002516	0.002494	0.002485
0.00248	0.002478	0.002472	0.002481	0.002482	0.002475	0.00251	0.00249	0.002472
0.00247	0.002479	0.002477	0.002477	0.002465	0.002467	0.002497	0.00248	0.002463
0.002857	0.002856	0.002862	0.002864	0.002873	0.002877	0.002875	0.002894	0.002887
0.002789	0.00279	0.002798	0.002795	0.002796	0.002821	0.002819	0.002818	0.00282
0.002786	0.002779	0.002788	0.002787	0.002801	0.002803	0.002812	0.002816	0.002808
0.002924	0.002919	0.002927	0.002919	0.002914	0.002923	0.002927	0.002934	0.00295
0.002859	0.002852	0.002851	0.002855	0.002853	0.002858	0.00285	0.002872	0.002889
0.002953	0.002947	0.002946	0.002943	0.00294	0.002951	0.002946	0.002963	0.002975
0.002587	0.002583	0.002585	0.002585	0.00258	0.002584	0.002592	0.002602	0.002581
0.002636	0.002637	0.002632	0.002637	0.002626	0.002643	0.002641	0.002659	0.002639
0.002614	0.002611	0.002615	0.002615	0.00261	0.002616	0.002624	0.002633	0.002616
0.001828	0.001829	0.001824	0.001826	0.001819	0.001818	0.001834	0.001865	0.001805
0.001901	0.001895	0.001897	0.001891	0.00188	0.001881	0.001892	0.001924	0.001867
0.00185	0.00185	0.001843	0.001842	0.001838	0.001836	0.001857	0.001883	0.001829
0.002306	0.0023	0.002305	0.002301	0.002296	0.002306	0.002332	0.002311	0.002299
0.002285	0.002288	0.002286	0.002292	0.002284	0.002291	0.002322	0.002296	0.002286
0.002335	0.002332	0.002337	0.002324	0.002326	0.002335	0.002361	0.002345	0.002325
0.003179	0.003177	0.003178	0.003187	0.003184	0.003209	0.003187	0.003191	0.003195
0.003174	0.003163	0.003175	0.003165	0.003172	0.003189	0.003173	0.003176	0.003187
0.003266	0.003272	0.003263	0.003265	0.003266	0.003271	0.003272	0.003274	0.003285
0.003032	0.003031	0.003033	0.003045	0.003028	0.003033	0.003037	0.003048	0.003039

0.003146	0.003138	0.003143	0.003136	0.003141	0.003146	0.003134	0.003152	0.003139
0.00288	0.002879	0.002876	0.002873	0.002881	0.002875	0.002889	0.002895	0.002881
0.002911	0.002926	0.002911	0.00291	0.00292	0.002923	0.00292	0.002925	0.002925
0.002931	0.00292	0.002927	0.002926	0.002922	0.002919	0.00292	0.002934	0.002935
0.002923	0.002928	0.002922	0.002917	0.00292	0.002926	0.002928	0.002935	0.002935
0.001988	0.001986	0.001988	0.001983	0.001983	0.001985	0.002008	0.001994	0.001968
0.001998	0.001994	0.001992	0.00199	0.001977	0.001991	0.002004	0.002002	0.001961
0.001994	0.001998	0.001994	0.001994	0.001986	0.001995	0.002014	0.002	0.001968

ppm	ppm	ppm	ppm	ppm	ppm	ppm	ppm	ppm
**9.66**	**9.7**	**9.74**	**9.78**	**9.82**	**9.86**	**9.9**	**9.94**	**9.98**
0.002565	0.002693	0.002637	0.002599	0.002598	0.002593	0.002535	0.002547	0.002541
0.002593	0.002709	0.002671	0.002629	0.00261	0.002648	0.002579	0.002577	0.002566
0.002551	0.002672	0.002634	0.00259	0.002577	0.002592	0.002528	0.002532	0.002524
0.002608	0.002611	0.002666	0.002617	0.0026	0.002639	0.002592	0.002587	0.002641
0.002634	0.002647	0.002698	0.002655	0.002627	0.002679	0.002625	0.002616	0.002666
0.002608	0.002611	0.002672	0.002615	0.002592	0.002647	0.002589	0.002593	0.002643
0.003033	0.003128	0.003054	0.00305	0.003041	0.003065	0.003038	0.00304	0.00305
0.002772	0.002832	0.002783	0.002771	0.002773	0.002785	0.002771	0.002768	0.002769
0.003053	0.003151	0.00308	0.00307	0.003066	0.003087	0.003061	0.003067	0.003068
0.002478	0.002466	0.003653	0.002905	0.002526	0.002658	0.002507	0.002497	0.002499
0.00248	0.002464	0.003702	0.00293	0.002514	0.002649	0.002505	0.002496	0.00249
0.002469	0.002467	0.003703	0.002898	0.002505	0.002636	0.002496	0.002483	0.002485
0.002895	0.002899	0.00295	0.003061	0.002944	0.002916	0.002906	0.002914	0.002924
0.002832	0.002835	0.002882	0.002994	0.002875	0.00285	0.002828	0.002841	0.002857
0.002821	0.00283	0.002875	0.002974	0.002857	0.002842	0.002818	0.002833	0.002845
0.002912	0.002919	0.002914	0.002922	0.002906	0.00294	0.002913	0.002912	0.002909
0.002853	0.002854	0.002849	0.002846	0.002851	0.002871	0.002851	0.002847	0.002832
0.002949	0.002954	0.002945	0.002945	0.002946	0.002957	0.002946	0.002947	0.002945
0.002592	0.002617	0.002615	0.002584	0.002606	0.002834	0.002525	0.002543	0.002535
0.002635	0.002667	0.002671	0.002637	0.00264	0.002936	0.002626	0.002617	0.002619
0.002619	0.00264	0.002648	0.002621	0.002623	0.002875	0.002594	0.002587	0.00259
0.001793	0.001804	0.010323	0.002705	0.001922	0.001909	0.001917	0.001857	0.001856
0.00184	0.00182	0.010052	0.00292	0.002034	0.00201	0.002009	0.001952	0.001941
0.001822	0.001831	0.010129	0.002722	0.001928	0.001918	0.001934	0.001869	0.001868
0.002277	0.002263	0.004805	0.002676	0.002366	0.002346	0.002352	0.002324	0.002318
0.002266	0.002268	0.004651	0.00264	0.002331	0.00233	0.002321	0.0023	0.002301
0.002296	0.002282	0.004754	0.002741	0.002419	0.002399	0.002391	0.002368	0.00236
0.003178	0.003181	0.003284	0.00319	0.003177	0.003205	0.00318	0.003173	0.003185
0.003185	0.003179	0.003287	0.003183	0.003173	0.00318	0.003162	0.003168	0.003164
0.003272	0.003263	0.003367	0.003292	0.003264	0.003285	0.00326	0.003261	0.003254
0.003039	0.003043	0.003069	0.003058	0.003047	0.00305	0.003035	0.003037	0.003037

0.003139	0.00315	0.003148	0.003157	0.003137	0.003139	0.003136	0.003138	0.003134
0.00289	0.002899	0.002924	0.002901	0.002892	0.002897	0.002883	0.00289	0.002887
0.002916	0.002922	0.002942	0.002935	0.002921	0.002935	0.002925	0.002911	0.002906
0.002932	0.002937	0.002949	0.00294	0.002927	0.002939	0.002931	0.002923	0.002919
0.002924	0.002933	0.002953	0.002938	0.002922	0.002936	0.002926	0.002918	0.00292
0.001953	0.001988	0.002935	0.002321	0.002193	0.009523	0.002161	0.002066	0.00209
0.001937	0.00196	0.002884	0.002282	0.001949	0.009781	0.002271	0.002145	0.002141
0.001949	0.001984	0.002916	0.0023	0.0022	0.009493	0.002207	0.00211	0.002124

ppm
**10.02**
0.002541
0.002573
0.00253
0.00261
0.002635
0.00261
0.003038
0.002773
0.003054
0.002497
0.002495
0.002485
0.002975
0.002904
0.002892
0.002916
0.002842
0.002944
0.002539
0.00262
0.002592
0.00184
0.001933
0.001866
0.002322
0.002297
0.002359
0.003176
0.003164
0.003263
0.003049

0.003136
0.002895

0.002915
0.002922
0.002915

0.002037
0.002087
0.002074
